# Targeting iron-associated protein Ftl1 in the brain of old mice improves age-related cognitive impairment

**DOI:** 10.1038/s43587-025-00940-z

**Published:** 2025-08-19

**Authors:** Laura Remesal, Juliana Sucharov-Costa, Yuting Wu, Karishma J. B. Pratt, Gregor Bieri, Amber Philp, Mason Phan, Turan Aghayev, Charles W. White, Elizabeth G. Wheatley, Bende Zou, Brandon R. Desousa, Julien Couthouis, Isha H. Jian, Xinmin S. Xie, Yi Lu, Jason C. Maynard, Alma L. Burlingame, Saul A. Villeda

**Affiliations:** 1 Department of Anatomy, University of California, San Francisco, San Francisco, CA USA; 2 Biomedical Sciences Graduate Program, University of California, San Francisco, San Francisco, CA USA; 3 Department of Chemistry, University of Illinois at Urbana-Champaign, Urbana, IL USA; 4 Department of Chemistry, The University of Texas at Austin, Austin, TX USA; 5 Developmental and Stem Cell Biology Graduate Program, University of California, San Francisco, San Francisco, CA USA; 6 AfaSci Research Laboratory, Redwood City, CA USA; 7 Department of Biochemistry, University of California, San Francisco, San Francisco, CA USA; 8 Gladstone Institute of Cardiovascular Disease, Gladstone Institutes, San Francisco, CA USA; 9 Department of Genetics, Stanford University School of Medicine, Stanford, CA USA; 10 Mass Spectrometry Facility, Department of Pharmaceutical Chemistry, University of California, San Francisco, San Francisco, CA USA; 11 Department of Physical Therapy and Rehabilitation Science, University of California, San Francisco, San Francisco, CA USA; 12 Bakar Aging Research Institute, San Francisco, CA USA

**Keywords:** Cognitive ageing, Hippocampus, Neural ageing, Molecular neuroscience, Ageing

## Abstract

Understanding cellular and molecular drivers of age-related cognitive decline is necessary to identify targets to restore cognition at old age. Here we identify ferritin light chain 1 (FTL1), an iron-associated protein, as a pro-aging neuronal factor that impairs cognition. Using transcriptomic and mass spectrometry approaches, we detect an increase in neuronal FTL1 in the hippocampus of aged mice, the levels of which correlate with cognitive decline. Mimicking an age-related increase in neuronal FTL1 in young mice alters labile iron oxidation states and promotes synaptic and cognitive features of hippocampal aging. Targeting neuronal FTL1 in the hippocampi of aged mice improves synaptic-related molecular changes and cognitive impairments. Using neuronal nuclei RNA sequencing, we detect changes in metabolic processes, such as ATP synthesis, and boosting these metabolic functions through NADH supplementation mitigated pro-aging effects of neuronal FTL1 on cognition. Our data identify neuronal FTL1 as a key molecular mediator of cognitive rejuvenation.

## Abstract (web summary)

The authors identify ferritin light chain 1 (FTL1), an iron-associated protein, as a pro-aging neuronal factor that increases with age and promotes cognitive decline. Targeting FTL1 in the brain improved cognition in old mice.

## Main

Investigating the interplay between aging in the brain and the molecular and cellular changes that lead to cognitive decline is key to understand susceptibility to age-related neurodegenerative diseases^1,2^. We and others have begun to challenge longstanding views of brain aging as a rigid process by showing that rejuvenation is possible in animal models at old age^3–9^. Notwithstanding, to identify potential therapeutic targets to restore cognitive function in older people, we first need to gain mechanistic insight into the molecular drivers of cognitive decline in the aging brain. It has become clear that cognitive dysfunction in the aged brain in the absence of neurodegenerative disease is not paralleled by cell death but, instead, by a decline in neuronal function at the synaptic level^1,10^. Although various neuronal factors have been shown to change with age in the brain^1,11^, to date, only a small number have been demonstrated as functional drivers of age-related cognitive dysfunction^12–15^. Thus, we sought to identify pro-aging neuronal factors as molecular targets for cognitive rejuvenation.

**Fig. 1:**
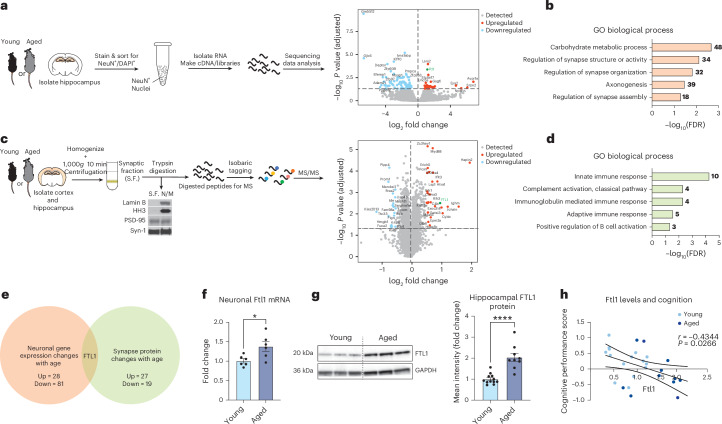
Neuronal FTL1 increases in the hippocampus with age and negatively correlates with cognitive function. **a**, Schematic of transcriptomics workflow for RNA-seq of hippocampal neuronal nuclei (*N* = 3 young (3 months) and *N* = 2 aged (18 months) mice). Volcano plot illustrating differential gene expression in hippocampal neurons of young compared to aged mice. Red dots represent significantly increased genes, and light blue dots represent significantly decreased genes. **b**, Gene Ontology (GO) terms of biological processes associated with upregulated and downregulated genes in hippocampal neurons during aging. Numerals indicate number of genes in each process. **c**, Schematic of proteomics workflow for mass spectrometry of cortical and hippocampal synaptosomes (*N* = 3 young (3 months) biological replicates (20 mice per replicate) and *N* = 3 aged (18–20 months) biological replicates (20 mice per replicate)). Volcano plot illustrating differential protein levels in synaptosomes of young compared to aged mice. Red dots represent significantly increased proteins, and light blue dots represent significantly decreased proteins. **d**, GO terms of biological processes associated with upregulated and downregulated proteins in synaptosomes during aging. Numerals indicate number of genes in each process. **e**, Venn diagram illustrating the two datasets used for candidate selection (*P* *=* 0.05 and FC ≥1.4). **f**, Quantification of neuronal *Ftl1* mRNA levels from young (3 months) and aged (18–20 months) mice by qPCR (*N* = 6 young and *N* = 6 aged mice (*P* = 0.0219)). **g**, Quantification of hippocampal FTL1 protein levels from young (3 months) and aged (24 months) mice by western blot (*N* = 12 young and *N* = 9 aged mice (*P* < 0.0001)). **h**, Pearson’s correlation between hippocampal FTL1 protein levels and overall cognition performance. Hippocampus-dependent learning and memory was assessed by Y maze, NOR and RAWM. A *z*-score was calculated for each behavioral test, and the average *z*-score across behavioral tests was used as a readout for overall cognitive performance (*N* = 15 young (3 months) and *N* = 11 aged (20 months) mice). Data are shown as mean ± s.e.m.; *P < 0.05, ****P < 0.0001, two-tailed *t*-test (**f**,**g**). FDR, false discovery rate; MS, mass spectrometry; FC, fold change; HH3, histone H3; S.F., synaptic fraction; N/M, nuclear/mitochondrial. Source data

We first investigated molecular changes in neurons that occur during aging in the hippocampus—a brain region regulating learning and memory and highly vulnerable to the effects of aging^11,13^—using neuronal nuclei RNA sequencing (RNA-seq). NeuN^+^ nuclei were isolated by fluorescence-activated cell sorting (FACS) from hippocampi of young (3 months) and aged (18 months) mice (Fig. 1a), and transcriptional changes were assessed by RNA-seq. Transcriptomic analysis detected 28 genes with increased expression and 81 genes with decreased expression with age (Fig. 1e and Supplementary Table 1). Gene Ontology analysis of differentially expressed genes predominantly identified synapse-related processes, such as regulation of synapse structure or activity and regulation of synapse assembly (Fig. 1b).

To gain insight into age-related molecular changes at the synaptic level, we performed comparative proteomics analysis of the synaptic fraction (synaptosome) in both young and aged mouse brains using isobaric tagging coupled with mass spectrometry (Fig. 1c). Due to technical limitations in the number of animals necessary to obtain sufficient amounts of isolated synaptosomes to conduct mass spectrometry analysis, we opted to sub-dissect and pool hippocampal and cortical tissue from mice. Proteomic analysis detected 27 proteins with increased abundance and 19 proteins with decreased abundance in aging (Fig. 1e and Supplementary Table 2). Gene Ontology analysis highlighted biological processes related to immune responses and complement activation, which are known to be associated with synaptic remodeling in neurons^16^ (Fig. 1d).

**Extended Data Fig. 1:**
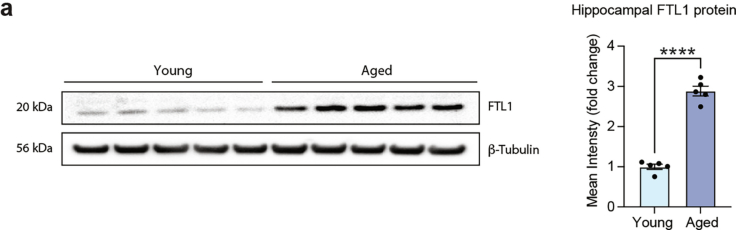
FTL1 increases with age in the mouse hippocampus. (**a**) Quantification of hippocampal FTL1 protein levels from young (3 months) and aged (18 months) mice by Western blot. N = 12 young and N = 9 aged mice (p-value < 0.0001). Data shown as mean±s.e.m.; ****P < 0.001, two-tailed t-test. Source data

Comparison of our neuronal transcriptomic and synaptic proteomic datasets identified one factor with conserved increased expression: the iron-associated protein ferritin light chain 1 (FTL1)^17–19^ (Fig. 1e–g and Extended Data Fig. 1a). To investigate the relationship between increased FTL1 and cognition, we correlated hippocampal FTL1 expression with cognitive performance on a battery of hippocampal-dependent learning and memory tasks in an independent cohort of young and aged mice. We observed a significant negative association between increased hippocampal FTL1 expression by western blot and impaired overall cognitive performance (Fig. 1g,h and Supplementary Table 3). These data identify FTL1 as a potential pro-aging neuronal factor with implications for cognitive dysfunction.

**Fig. 2:**
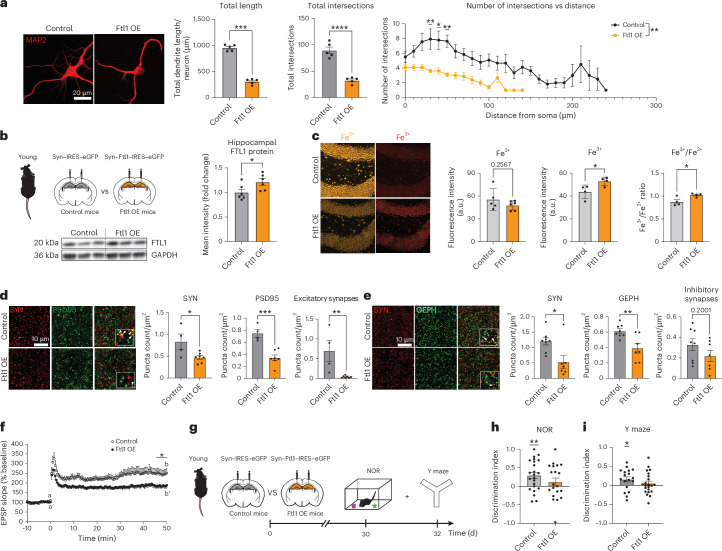
Increasing neuronal FTL1 in the young hippocampus promotes aging-associated synaptic molecular changes and cognitive impairments. **a**, Representative images and quantification of total neurite length and Sholl analysis of primary mouse neurons after viral-mediated *Ftl1* overexpression (Ftl1 OE) or control. Neurons were infected at DIV8 and analyzed at DIV18. Five images per coverslip were taken, and 5–9 neurons were analyzed per coverslip, with the average per coverslip serving as a replicate (*N* = 5 control and *N* = 5 Ftl1 OE replicates per group) (total length *P* < 0.0001; total intersections *P* < 0.0001; intersections versus distance *P* = 0.0054). **b**, Schematic of hippocampal stereotaxic injections of young mice after neuronal-specific viral-mediated overexpression of *Ftl1* (Ftl1 OE) or GFP control and representative western blot and quantification of hippocampal FTL1 protein (*N* = 6 control and *N* = 6 Ftl1 OE mice per group (*P* = 0.0434)). **c**, Representative images and quantification of labile Fe^3+^, Fe^2+^ and Fe^3+^/Fe^2+^ ratio in young mice hippocampus after neuronal-specific viral-mediated overexpression of (Ftl1 OE) or GFP (control) (*N* = 5 control and *N* = 6 Ftl1 OE for Fe^2+^ (*P* = 0.2567); *N* = 4 per group for Fe^3+^ (*P* = 0.0382) and Fe^3+^/Fe^2+^ ratio (*P* = 0.0279)). **d**, Representative images and quantification of excitatory synapses in the hippocampus of mice after neuron-specific, viral-mediated overexpression of *Ftl1* (Ftl1 OE) or GFP (control) (*N* = 4 control and *N* = 7 Ftl1 OE mice per group (SYN *P* = 0.0265, PSD95 *P* = 0.0006; excitatory *P* = 0.0089)). **e**, Representative images and quantification of inhibitory synapses in the hippocampus of mice after neuronal-specific viral-mediated overexpression of *Ftl1* (Ftl1 OE) or GFP (control) (*N* = 8 control and *N* = 6 Ftl1 OE mice per group (SYN *P* = 0.0129, GEPH *P* = 0.0053; inhibitory *P* = 0.2001)). **f**, EPSP recorded from the hippocampus of young mice after neuron-specific, viral-mediated overexpression of *Ftl1* (Ftl1 OE) or GFP (control). LTP levels shown represent the average of the last 5 minutes (*N* = 16 control and *N* = 16 Ftl1 OE mice per group (*P* = 0.029)). **g**, Schematic of the timeline of hippocampal stereotaxic injections and cognitive behavioral testing for young mice overexpressing *Ftl1*. **h**, Object recognition memory assesed by NOR as the discrimination index for the novel object relative to the familiar object (*N* = 23 control (*P* = 0.0023) and *N* = 20 Ftl1 OE (*P* = 0.2764) young mice per group). **i**, Spatial working memory assessed by Y maze as the discrimination index for the novel arm relative to the trained arm (*N* = 18 control (*P* = 0.0336) and *N* = 22 Ftl1 OE (*P* = 0.4315) young mice per group). Data are shown as mean ± s.e.m; *P < 0.05, **P < 0.001, ***P < 0.0001, two-tailed *t*-test (**a**–**e**), two-way ANOVA with Sidak’s post hoc test (**a**), unpaired Studentʼs *t*-test (**g**) and one-sample *t*-test with theoretical mean 0% (**h**,**i**). d, days; GEPH, gephyrin; min, minutes; SYN, synapsin. Source data

**Extended Data Fig. 2:**
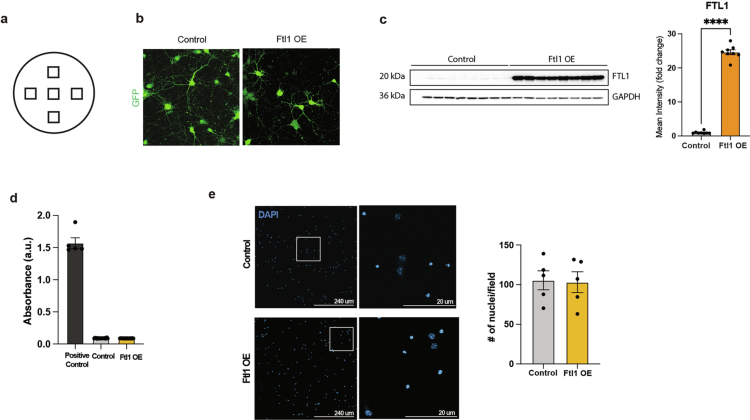
Ftl1 overexpression in primary neurons does not induce cell death in vitro. Primary neurons were infected with lentivirus encoding Ftl1 or control at 8 days in vitro (DIV8) and analyzed at DIV18. (**a**) Illustration showing the coverslip regions used for neurite analysis of primary neurons following lentiviral-mediated overexpression of Ftl1 (Ftl1 OE) or control. (**b**) Representative images of GFP-positive neurons used as a redout for lentiviral infection for both Ftl1 OE and control conditions. (**c**) Representative Western blot and quantification of FTL1 protein levels N = 8 replicates/condition (p-value < 0.0001). (**d**) Quantification of cell death in primary neurons following viral-mediated overexpression of Ftl1 by LDH assay. N = 5 positive control, N = 5 control and N = 5 Ftl1 OE replicates/condition (p-value < 0.0001). (**e**) Representative images and quantification of DAPI-positive nuclei of Ftl1 OE and control neurons at DIV18. N = 5 controls and N = 5 Ftl1 OE replicates/condition (p-value = 0.8956). Data shown as mean±s.e.m.; ****P < 0.001, two-tailed t-test (**c,d,e**). Source data

To assess the effect of mimicking an age-related increase in neuronal FTL1, we used a cell-type-specific, viral-mediated overexpression approach. Primary mouse neurons were infected with lentiviral constructs encoding either *Ftl1* or green fluorescent protein (GFP) under the control of the neuron-specific synapsin-1 promoter (Fig. 2a and Extended Data Fig. 2a–c). We assessed morphological changes by immunocytochemical analysis and detected a decrease in the total neurite length and dendritic complexity of MAP2^+^ neurons overexpressing FTL1 compared to control (Fig. 2a). No differences in neuronal cell density or toxicity were observed under FTL1 overexpression conditions (Extended Data Fig. 2d,e).

**Extended Data Fig. 3:**
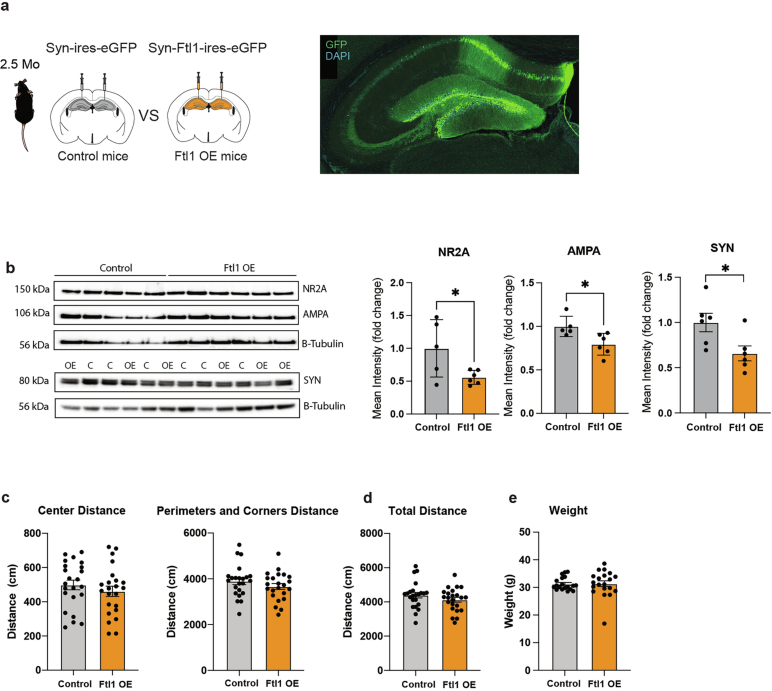
Neuronal Ftl1 overexpression decreases synaptic related proteins in the young hippocampus but does not alter health metrics. (**a**) Schematic of hippocampal stereotaxic injections of young mice following neuronal specific viral-mediated overexpression of Ftl1 (Ftl1 OE) or GFP control (left). (**a**) Representative image of infected GFP-positive neurons (right). (**b**) Representative Western blot and quantification of synaptic proteins in the hippocampus of mice following viral-mediated Ftl1 overexpression (Ftl1 OE) or GFP (control). N = 5 control and N = 6 Ftl1 OE mice/group. NR2A (p-value = 0.0390), AMPA (p-value = 0.0206), SYN (p-value = 0.0258). (**c-e**) Baseline anxiety and activity assessed by open field (**c**) Center distance p-value = 0.3739, Perimeters and corners distance p-value = 0.2415, (**d**) Total distance p-value = 0.2198 and (**e**) weight (p-value = 0.9237) of young mice overexpressing Ftl1 or control. N = 23 control and N = 24 young Ftl1 OE mice. Data shown as mean±s.e.m.; *P < 0.05; two-tailed t-test. Source data

Having observed neuronal changes in vitro, we investigated the effect of increasing neuronal FTL1 in the adult hippocampus. Young mice were given bilateral stereotaxic injections of high-titer lentivirus encoding *Ftl1* or GFP under the control of the neuron-specific synapsin-1 promoter into the CA1 and dentate gyrus regions of the hippocampus (Fig. 2b and Extended Data Fig. 3a,b). Increased FTL1 expression was validated by western blot (Fig. 2b).

To begin, we characterized changes in redox states of free iron (Fe) after increased neuronal FTL1 in the hippocampus using DNAzyme-based fluorescent turn-on sensors that are selective for either ferrous (Fe^2+^) or ferric (Fe^3+^) states^20^. Fe^2+^ and Fe^3+^ were detected simultaneously in the hippocampus, and an increase in Fe^3+^ was observed in neurons overexpressing FTL1 (Fig. 2c). To further understand changes in iron redox cycling, we compared the relative Fe^3+^/Fe^2+^ ratio and observed a concomitant increase after neuronal FTL1 overexpression (Fig. 2c). These data suggest that increased FTL1 promotes accumulation of oxidized iron in hippocampal neurons.

To examine aging-associated synaptic alterations^14^, we assessed presynaptic and postsynaptic molecular markers after increased neuronal FTL1 in the hippocampus. We assessed changes in synapses by immunocytochemical analysis and detected a decrease in PSD95^+^ excitatory and gephyrin^+^ inhibitory synapses in the hippocampus of mice overexpressing neuronal FTL1 compared to control (Fig. 2d,e). Additionally, we observed reduced expression of glutamate NMDA receptor subunit 2A (NR2A), AMPA receptor and synapsin by western blot (Extended Data Fig. 3c), consistent with previously described age-related changes associated with impairments in synaptic plasticity^14^. Our data suggest that increasing neuronal FTL1 promotes aging-associated synaptic changes in the adult hippocampus.

To investigate the functional impact of increasing neuronal FTL1 in the hippocampus, we assessed long-term potentiation (LTP) and cognition in young mice after viral-mediated neuronal FTL1 overexpression (Fig. 2f–i). We performed extracellular electrophysiological recordings on hippocampal slices and observed lower LTP levels from the CA1 of young animals overexpressing neuronal FTL1 compared to control conditions (Fig. 2f). Hippocampal-dependent memory was assessed using novel object recognition (NOR) and forced alternation Y maze behavioral paradigms (Fig. 2g). During NOR and Y maze testing, control young mice were biased toward a novel object and the novel arm relative to a familiar condition (Fig. 2h,i). However, young mice with increased expression of neuronal FTL1 were cognitively impaired, demonstrating no preference (Fig. 2h,i). As a control, we profiled general health using an open-field paradigm and observed no differences in overall activity, total distance traveled or time spent in the center of the open field, indicative of normal motor and anxiety functions (Extended Data Fig. 3d–f). Our functional data indicate that mimicking an age-related increase in neuronal FTL1 negatively regulates synaptic plasticity and hippocampal-dependent memory.

**Fig. 3:**
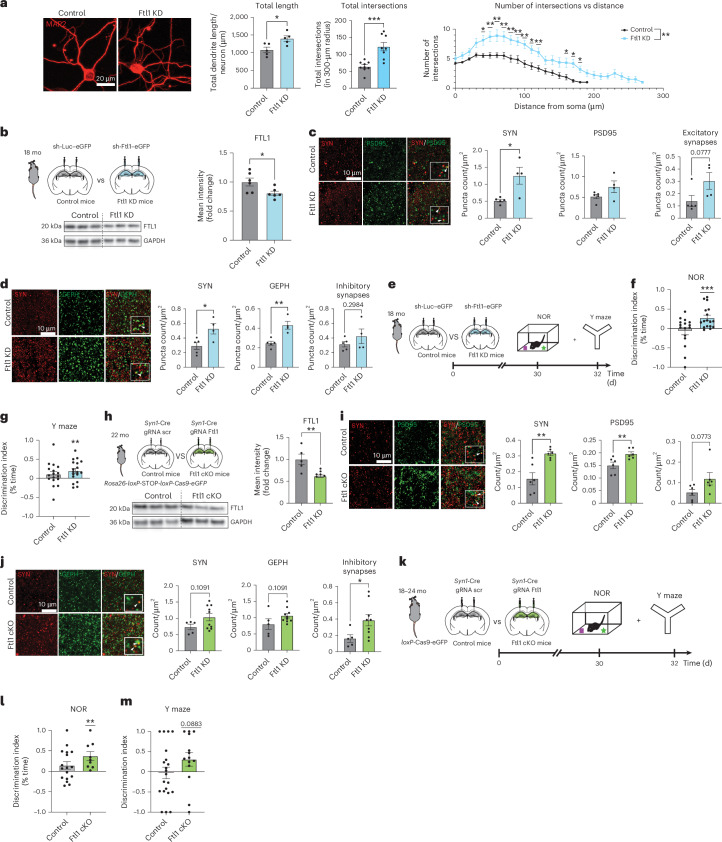
Targeting neuronal FTL1 in the aged hippocampus increases synaptic protein expression and rescues age-related cognitive impairments. **a**, Representative images and quantification of total neurite length and Sholl analysis of primary mouse neurons after viral-mediated knockdown of *Ftl1* (Ftl1 KD) or luciferase (control). Neurons were infected at DIV8 and analyzed at DIV18. Five images per coverslip were taken, and 5–9 neurons were analyzed per coverslip, with the average per coverslip serving as a replicate (*N* = 5 control and *N* = 5 Ftl1 KD replicates per group) (total length *P* = 0.0182; total intersections *P* = 0.0004; intersections versus distance *P* < 0.0001). **b**, Schematic of hippocampal stereotaxic injections of aged (18 months) mice after viral-mediated knockdown of *Ftl1* (Ftl1 KD) or control and representative western blot and quantification of hippocampal FTL1 protein (*P* = 0.0323). **c**, Representative images and quantification of excitatory synapses in the hippocampus of mice after viral-mediated *Ftl1* knockdown (Ftl1 KD) or control (*N* = 5 control and *N* = 4 Ftl1 KD mice per group (SYN *P* = 0.0132, PSD95 *P* = 0.1324; excitatory *P* = 0.0777)). **d**, Representative images and quantification of inhibitory synapses in the hippocampus of mice after viral-mediated *Ftl1* knockdown (Ftl1 KD) or control (*N* = 5 control and *N* = 4 Ftl1 KD mice per group (SYN *P* = 0.0257, GEPH *P* = 0.0031; inhibitory *P* = 0.2984)). **e**, Schematic of the timeline of hippocampal stereotaxic injections and cognitive behavioral testing for aged mice after *Ftl1* abrogation (Ftl1 KD). **f**, Object recognition memory assesed by NOR as the discrimination index for the novel object relative to the familiar object (*N* = 16 control (*P* = 0.4977) and *N* = 17 Ftl1 KD (*P* = 0.0010) aged mice per group). **g**, Spatial working memory assessed by Y maze as the discrimination index for the novel arm relative to the trained arm (*N* = 17 control (*P* = 0.1895) and *N* = 18 Ftl1 KD (*P* = 0.0072) aged mice per group). **h**, Schematic of hippocampal stereotaxic injections of aged *Rosa26-loxP-*STOP*-loxP-Cas9-eGFP* transgenic mice after neuronal-specific viral-mediated expression of guide RNAs targeting *Ftl1* (Ftl1 cKO) or control and representative western blot and quantification of hippocampal FTL1 protein (*N* = 5 control and *N* = 6 Ftl1 cKO mice per group (*P* = 0.0076)). **i**, Representative images and quantification of excitatory synapses in the hippocampus of Ftl1 cKO or control mice (*N* = 6 control and *N* = 6 Ftl1 cKO mice per group (SYN *P* = 0.0028, PSD95 *P* = 0.0140; excitatory *P* = 0.0773)). **j**, Representative images and quantification of inhibitory synapses in the hippocampus of Ftl1 cKO or control mice (*N* = 5 control and *N* = 9 Ftl1 cKO mice per group (SYN *P* = 0.1091, GEPH *P* = 0.1091, inhibitory *P* = 0.0472)). **k**, Schematic of the timeline of hippocampal stereotaxic injections and cognitive behavioral testing for aged *Rosa26-loxP-*STOP*-loxP-Cas9-eGFP* transgenic mice with loss of neuronal *Ftl1*. **i**, Object recognition memory assesed by NOR as the discrimination index for the novel object relative to the familiar object (*N* = 17 aged control (*P* = 0.1423) and *N* = 9 aged Ftl1 cKO (*P* = 0.0087) mice per group). **j**, Spatial working memory assessed by Y maze as the discrimination index for the novel arm relative to the trained arm (*N* = 21 aged control (*P* = 0.5796) and *N* = 14 aged Ftl1 cKO (*P* = 0.2676) mice per group). Data are shown as mean ± s.e.m; *P < 0.05, **P < 0.005, ***P < 0.0001, two-tailed *t*-test (**a**–**d**,**h**–**j**), two-way ANOVA with Sidak’s post hoc test (**a**) and one-sample *t*-test with theoretical mean 0% (**f**,**g**,**l**,**m**). d, days; GEPH, gephyrin; mo, months; SYN, synapsin; scr, scramble. Source data

**Extended Data Fig. 4:**
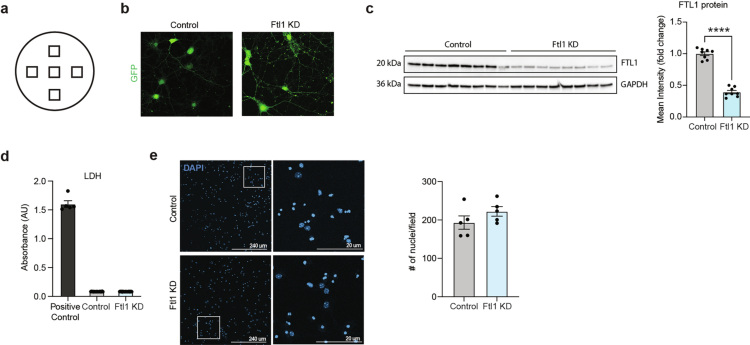
Abrogation of Ftl1 in primary neurons does not induce cell death in vitro. Primary neurons were infected with lentivirus encoding shRNA targeting Ftl1 or control at 8 days in vitro (DIV8) and analyzed at DIV18. (**a**) Illustration showing the coverslip regions used for neurite analysis of primary neurons following lentiviral-mediated Ftl1 knockdown (Ftl1 KD) or control. (**b**) Representative images of GFP-positive neurons used as a redout for lentiviral infection for both Ftl1 KD and control conditions. (**c**) Representative Western blot and quantification of FTL1 protein levels. N = 8 replicates/condition N= (p-value < 0.0001) (**d**) Quantification of cell death in primary neurons following viral-mediated overexpression of Ftl1 by LDH assay. (**d**) Representative images and quantification of DAPI-positive nuclei of Ftl1 KD and control neurons at DIV18. N = 5 positive control, N = 5 control and N = 5 Ftl1 KD replicates/condition (p-value < 0.0001). (**e**) Representative images and quantification of DAPI-positive nuclei of Ftl1 KD and control neurons at DIV18. N = 5 controls and N = 5 Ftl1 KD replicates/condition (p-value = 0.2130). Data shown as mean±s.e.m.; ****P < 0.001, two-tailed t-test (**c,d,e**). Source data

To begin to investigate the converse effect of abrogating neuronal *Ftl1*, we used a complementary viral-mediated RNA interference in vitro approach. Primary neurons were infected with lentivirus encoding short hairpin RNA (shRNA) sequences targeting *Ftl1* (sh-Ftl1) or *luciferase* control (sh-Luc) (Fig. 3a and Extended Data Fig. 4a–c). Decreased neuronal FTL1 expression (Extended Data Fig. 4c) resulted in an increase in total neurite length as well as in dendritic complexity of MAP2^+^ neurons by immunocytochemical analysis (Fig. 3a). No differences in neuronal cell density or toxicity were observed under RNA interference conditions (Extended Data Fig. 4d,e).

**Extended Data Fig. 5:**
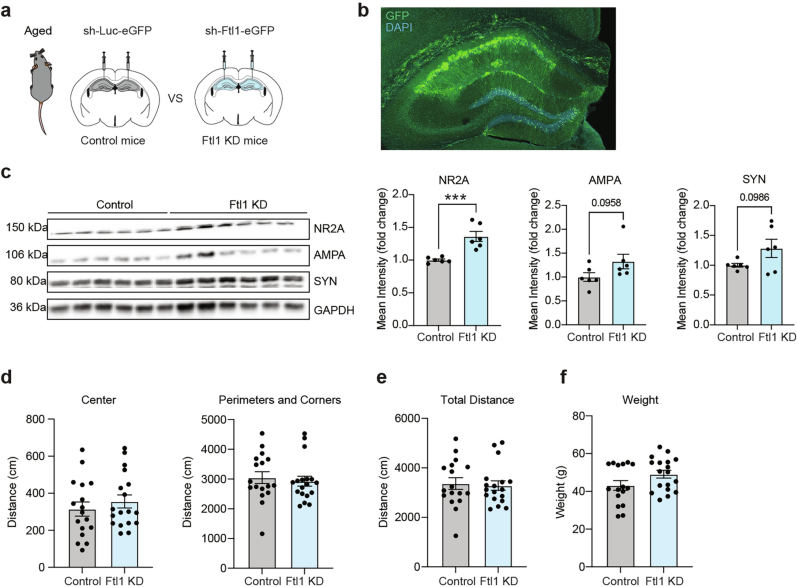
Abrogation of Ftl1 increases synaptic related proteins in the aged hippocampus but does not alter health metrics. (**a**) Schematic of hippocampal stereotaxic injections of aged mice following neuronal specific viral-mediated overexpression of Ftl1 (Ftl1 OE) or control. (**b**) Representative image of infected GFP-positive cells. (**c**) Representative Western blot and quantification of synaptic proteins in the hippocampus of Ftl1 KD and control mice following viral infection. N = 6 control and N = 6 Ftl1 OE mice/group. (**d-f**) Baseline anxiety and activity assessed by open field (**d**) Center distance p-value = 0.4282, Perimeters and corners distance p-value = 0.6428, (**e**) Total distance p-value = 0.7927 and (**f**) weight (p-value = 0.0787) of aged mice following abrogation of Ftl1 or control. N = 18 aged Ftl1 KD and N = 17 control mice. Data shown as mean±s.e.m.;*P < 0.05; ***P < 0.001; two-tailed t-test. Source data

Next, we sought to explore the potential benefit of targeting the age-related increase of FTL1 in the hippocampus of aged mice. We performed targeted stereotaxic injections of a high-titer virus encoding shRNA sequences targeting either *Ftl1* or *luciferase* control into the CA1 and dentate gyrus hippocampal regions of aged mice (Fig. 3b and Extended Data Fig. 5a,b). Abrogation of FTL1 was validated by western blot (Fig. 3b). We assessed changes in synapses by immunocytochemical analysis, and, in contrast to the effects observed with FTL1 overexpression, targeting *Ftl1* led to an increase in PSD95^+^ excitatory and gephyrin^+^ inhibitory synapses in the aged hippocampus (Fig. 3c,d). Additionally, we observed an increase in NR2A, AMPA receptor and synapsin by western blot (Extended Data Fig. 5c). Functionally, hippocampal-dependent learning and memory was assessed using NOR and forced alternation Y maze in aged mice after viral-mediated neuronal FTL1 knockdown (Fig. 3e). Consistent with age-related cognitive decline, control aged mice showed no preference for either a novel object or a novel arm relative to a familiar condition (Fig. 3f,g). Notably, abrogating FTL1 in the aged hippocampus resulted in cognitive improvements (Fig. 3f,g). No differences in overall activity were detected between experimental groups (Extended Data Fig. 5d–f). These data suggest that targeting hippocampal FTL1 restores known age-related synaptic molecular changes and cognitive function in aged mice.

**Extended Data Fig. 6:**
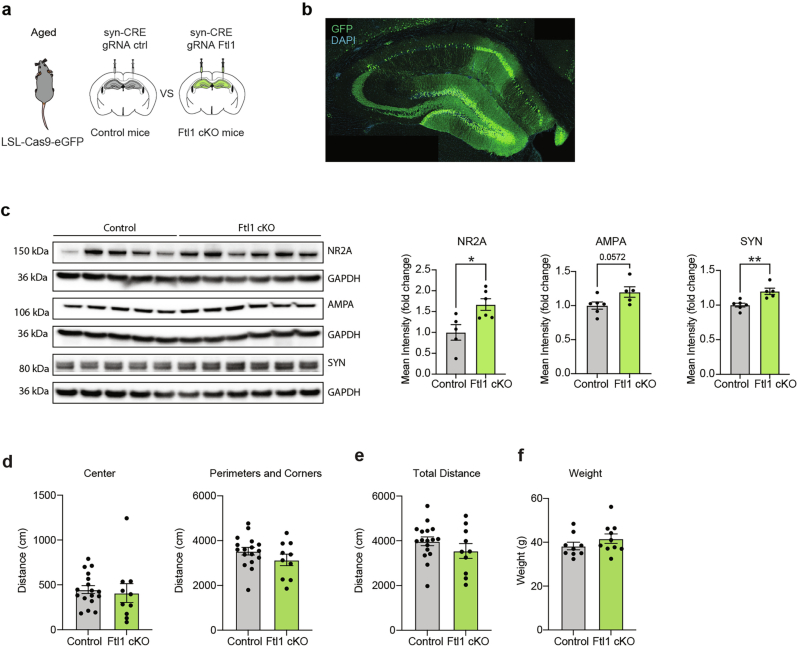
Loss of neuronal Ftl1 decreases synaptic related proteins in the aged hippocampus but does not alter health metrics. (**a**) Schematic of hippocampal stereotaxic injections of aged Rosa26-fl-stop-fl-Cas9-eGFP transgenic mice following neuronal specific viral-mediated expression of guide RNAs targeting Ftl1 (Ftl1 cKO) or control (left). (**b**) Representative image of infected GFP-positive neurons (right). (**c**) Representative Western blot and quantification of synaptic proteins in the hippocampus of Ftl1 cKO and control mice following neuronal specific viral infection. N = 6 control and N = 6 Ftl1 cKO mice/group. NR2A (p-value = 0.0166), AMPA (p-value = 0.0572), SYN (p-value = 0.0040). (**c-e**) Baseline anxiety and activity assessed by open field (**c**) Center distance p-value = 0.7044, Perimeters and corners distance p-value = 0.1993, (**d**) Total distance p-value = 0.2451 and (**f**) weight (p-value = 0.2408) of aged mice following abrogation of Ftl1 or control. N = 18 aged Ftl1 KD and N = 17 control mice. Data shown as mean±s.e.m.; ***P < 0.001; two-tailed t-test. Source data

To determine the effect of selectively targeting the age-related increase in neuronal FTL1, we generated aged, temporally controlled, neuron-specific conditional *Ftl1* genetic knockout mice (FTL1 cKO) using a viral-mediated in vivo CRISPR–Cas9 approach by delivering high-titer lentivirus encoding a synapsin-driven *Cre* and guide RNA sequences into aged inducible Cas9 transgenic mice (Fig. 3h and Extended Data Fig. 6a,b). Decreased FTL1 levels were validated by western blot (Fig. 3h). We observed an increase in PSD95^+^ excitatory and gephyrin^+^ inhibitory synapses by immunocytochemical analysis and an increase in NR2A, AMPA receptor and synapsin by western blot in the hippocampus of aged FTL1 cKO mice compared to controls (Fig. 3i,j and Extended Data Fig. 6c). Hippocampal-dependent learning and memory was assessed using NOR and forced alternation Y maze in aged FTL1 cKO mice (Fig. 3k). Consistent with abrogation of hippocampal FTL1, selectively targeting neuronal *Ftl1* resulted in cognitive improvements in memory in FTL1 cKO mice compared to control mice (Fig. 3l,m). No differences in overall activity were detected between experimental groups (Extended Data Fig. 6d–f). These behavioral data indicate that targeting neuronal FTL1 at old age mitigates age-related cognitive impairments.

**Fig. 4:**
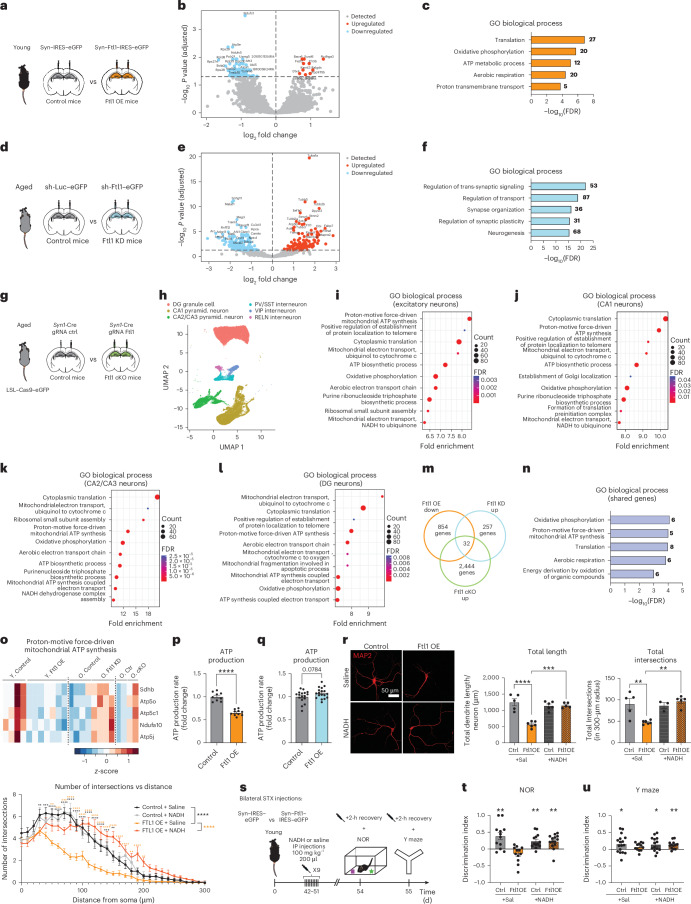
Pro-aging effects of increased neuronal FTL1 on cognitive function are mediated in part through alterations in metabolic processes. **a**, Schematic of hippocampal stereotaxic injections of young mice after neuronal-specific viral-mediated overexpression of *Ftl1* (Ftl1 OE) or GFP control. **b**, Volcano plot illustrating differential gene expression in hippocampal neurons of Ftl1 OE compared to control mice. Red dots represent significantly upregulated genes, and light blue dots represent significantly downregulated genes. **c**, Gene Ontology (GO) terms of biological processes associated with upregulated and downregulated genes. **d**, Schematic of hippocampal stereotaxic injections of aged (18 months) mice after viral-mediated knockdown of *Ftl1* (Ftl1 KD) or control. **e**, Volcano plot illustrating differential gene expression in hippocampal neurons of aged Ftl1 KD mice compared to aged control mice. Red dots represent significantly upregulated genes, and light blue dots represent significantly downregulated genes. **f**, GO terms of biological processes associated with upregulated and downregulated genes. **g**, Schematic of hippocampal stereotaxic injections of aged *Rosa26-loxP-*STOP*-loxP-Cas9-eGFP* transgenic mice after neuronal-specific viral-mediated expression of guide RNAs targeting *Ftl1* (Ftl1 cKO) or control. **h**, Combined two-dimensional visualization of single-nuclei clusters in the hippocampus from aged Ftl1 cKO and control mice (*N* = 2 pooled mice per group). **i**–**l**, GO terms of biological processes associated with upregulated and downregulated genes identified through single-nuclei RNA-seq analysis in excitatory neuron (**i**), CA1 (**j**), CA2/CA3 (**k**) and DG (**l**) clusters of aged Ftl1 cKO and control mice. **m**, Venn diagram illustrating three RNA-seq datasets used for gene expression comparison among Ftl1 OE, Ftl1 KD and Ftl1 cKO conditions. **n**, GO terms of biological processes associated with 32 shared genes identified across all conditions. **o**, Heatmap of proton-motive force-driven mitochondrial ATP synthesis. **p**, Mitochondrial ATP production rate in primary neurons after viral-mediated *Ftl1* overexpression on DIV0 quantified by Seahorse on DIV11 (*N* = 9 control and *N* = 10 Ftl1 OE replicates per condition (*P* < 0.0001)). **q**, Mitochondrial ATP production rate in primary neurons after viral-mediated Ftl1 KD on DIV8 quantified by Seahorse on DIV11 (*N* = 17 control and *N* = 18 replicates per condition (*P* = 0.0784)). **r**, Representative images of total neurite length and Sholl analysis of primary mouse neurons after viral-mediated *Ftl1* overexpression (Ftl1 OE) or control, treated with NADH at 200 µM or saline daily for 5 days. Neurons were infected at DIV8, treated with NADH from DIV14 to DIV18 and analyzed at DIV18 (*N* = 5 control+saline, *N* = 5 Ftl1 OE+saline, *N* = 6 control+NADH and *N* = 6 Ftl1 OE+NADH replicates per group) (total length (control+saline versus OE+saline *P* = < 0.0001; OE+saline versus OE+NADH *P* = 0.0002); total intersections (control+saline versus OE+saline *P* = 0.0075; OE+saline versus OE+NADH *P* = 0.0039); intersections versus distance (control+saline versus OE+saline *P* = <0.0001; OE+saline versus OE+NADH *P* = <0.0001)). **s**, Schematic of the timeline of hippocampal stereotaxic injections and cognitive testing for young Ftl1 OE with control mice treated with either NADH or saline control. **t**, Object recognition memory assesed by NOR as the discrimination index for the novel object relative to the familiar object (*N* = 12 control+saline (*P* = 0.0046), *N* = 12 Ftl1 OE+saline (*P* = 0.0616), *N* = 13 control+NADH (*P* = 0.0016), *N* = 14 Ftl1 OE+NADH (*P* = 0.0017)). **u**, Spatial working memory assessed by Y maze as the discrimination index for the novel arm relative to the trained arm (*N* = 17 control+saline (*P* = 0.0250), *N* = 12 Ftl1 OE+saline (*P* = 0.0551), *N* = 14 control+NADH (*P* = 0.0238), *N* = 13 Ftl1 OE+NADH (*P* = 0.0020)). Data are shown as mean ± s.e.m; *P < 0.05, **P < 0.005, ***P < 0.0001, ****P < 0.0001, two-tailed *t*-test (**p**,**q**), two-way ANOVA with Tukey’s post hoc test (**r**) and one-sample *t*-test with theoretical mean 0% (**t**,**u**). Ctrl, control; d, days; DG, dentate gyrus; FDR, false discovery rate; h, hour; min, minutes; IP, intraperitoneal; pyramid., pyramidal; Sal, saline; STX, stereotaxic; tr, treatment.

**Extended Data Fig. 7:**
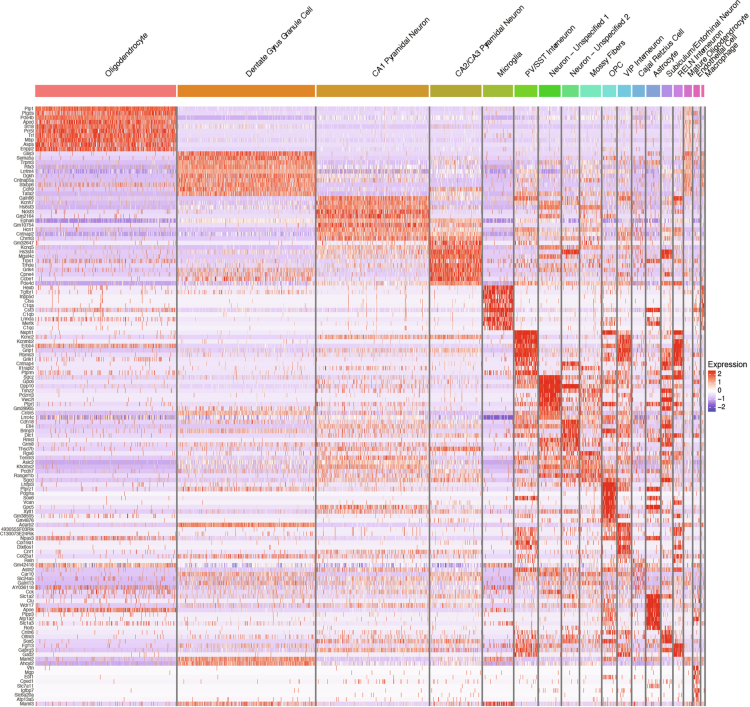
Single-nuclei RNA-seq gene signature for each cell cluster. Heatmap of the expression levels of the top marker genes for each of the 18 cell clusters.

**Extended Data Fig. 8:**
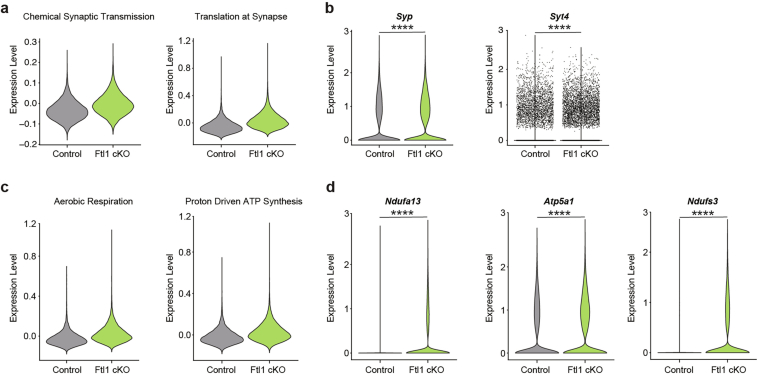
Neuronal single-nuclei expression of enriched GO biological processes. (**a**) Violin plot of module score of differentially expressed genes within significantly enriched GO biological processes relating to neuronal signaling transmission. (**b**) Violin plot of representative genes within GO biological processes relating to neuronal signaling transmission. (**c**) Violin plot of module score of differentially expressed genes within significantly enriched GO biological processes relating to mitochondrial processes. (**d**) Violin plot of representative genes within GO biological processes relating to mitochondrial processes.; ****P adj<0.001, DESeq2, Wald test.

To identify potential mechanisms underlying the pro-aging effects of increased neuronal FTL1 on cognitive function, we characterized the transcriptomes of hippocampal neurons from young and aged mice after viral-mediated FTL1 overexpression and abrogation, respectively, by neuronal nuclei RNA-seq approaches (Fig. 4a,d). We detected 100 differentially expressed genes in young mice overexpressing neuronal FTL1 and 309 differentially expressed genes in aged mice after hippocampal FTL1 knockdown compared to control conditions (Fig. 4b,e and Supplementary Tables 4 and 5). We performed Gene Ontology analysis and identified changes in energy metabolism pathways and synaptic processes (Fig. 4c,f). To gain further regional and cell-type-specific resolution, we assessed molecular changes elicited by viral-mediated *Ftl1* knockout by single-nuclei RNA-seq (Fig. 4g). Cell clusters were identified using a principal component analysis (PCA)-based approach and projected by uniform manifold approximation and projection (UMAP) onto a two-dimensional plot (Fig. 4h). Signatures of each cluster were generated by differential gene expression analysis to compare clusters. Cell types were established based on transcriptomic signatures, and populations were compared across genotypes (Extended Data Fig. 7). We performed bioinformatics analysis on excitatory hippocampal neurons, and Gene Ontology analysis of differentially expressed genes between FTL1 cKO and control mice identified predominantly metabolic processes (Fig. 4i). Concordantly, we observed increased expression of genes related to aerobic respiration and proton-driven ATP synthesis in excitatory neurons of FTL1 cKO mice (Extended Data Fig. 8a–d). Next, we analyzed clusters of CA1 pyramidal neurons, CA2/CA3 pyramidal neurons and dentate gyrus granule cell neurons separately (Fig. 4j–l). Gene Ontology analysis of differentially expressed genes identified metabolic processes associated with ATP production, oxidative phosphorylation and NADH across hippocampal neuronal cell types (Fig. 4j–l).

Leveraging our transcriptomic analysis, we next surveyed our in vivo neuronal FTL1 overexpression RNA-seq (Fig. 4a), in vivo neuronal *Ftl1* RNA interference RNA-seq (Fig. 4d) and in vivo *Ftl1* cKO single-nuclei RNA-seq (Fig. 4g) datasets for convergent molecular pathways. Of the genes that are downregulated in the young hippocampus after increased neuronal FTL1 and upregulated in the aged hippocampus after abrogation of neuronal FTL1, we detected 32 genes that were bidirectionally changed (Fig. 4m and Supplementary Table 6). Gene Ontology analysis of this gene set identified metabolic processes associated with ATP production and oxidative phosphorylation (Fig. 4n), with restored neuronal expression of genes including *Sdhb*, *Atp5o*, *Atp5c1*, *Ndufa10* and *Atp5j* in the aged hippocampus after abrogation of neuronal FTL1 (Fig. 4o). To corroborate our RNA-seq findings using an orthogonal approach, we measured neuronal ATP production rate by Seahorse assay after viral-mediated overexpression and abrogation of FTL1 in primary neurons. Increasing neuronal FTL1 significantly compromised ATP production (Fig. 4p), whereas abrogation of FTL1 resulted in increased ATP production compared to control conditions (Fig. 4q).

**Extended Data Fig. 9:**
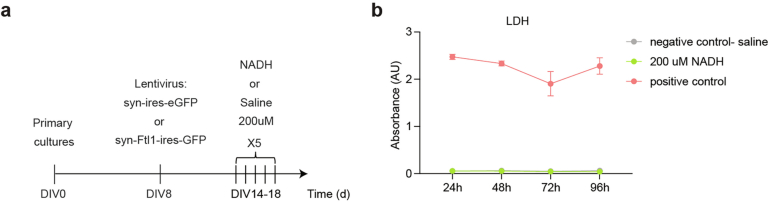
Ftl1 overexpression and NADH treatment does not induce cell death in primary neurons in vitro. Primary neurons were infected with lentivirus encoding Ftl1 or control at 8 days in vitro (DIV8) subsequently treated with NADH (200uM) for 5 consecutive days starting at DIV14 and analyzed at DIV18. (**a**) Schematic illustrating experimental timeline for lentiviral-mediated overexpression of Ftl1 (Ftl1 OE) or control and NADH treatment in primary neurons. (**b**) Quantification of cell death in primary neurons following viral-mediated overexpression of Ftl1 and NADH treatment by Lactate Dyhydrogenase (LDH) assay. Each data point represents the average of 3 replicates. Data shown as mean±s.e.m, two-way ANOVA followed by Tukey’s post hoc t-test.

**Extended Data Fig. 10:**
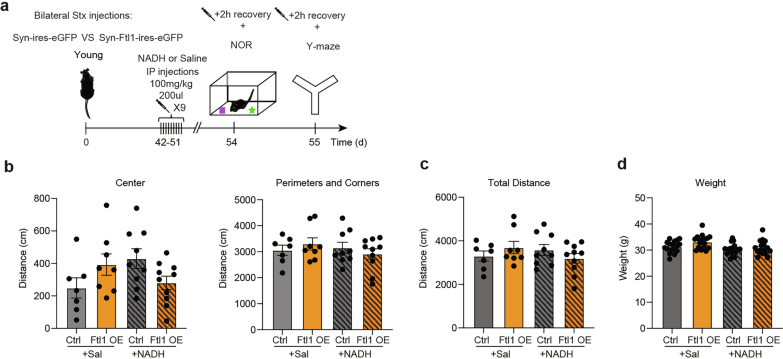
NADH administration does not alter health metrics in young mice following neuronal Ftl1 overexpression. (**a**) Schematic of the timeline of hippocampal stereotaxic injections and behavioral assessment for young mice overexpressing Ftl1 (Ftl1 OE) with control mice treated with either NADH (100 mg/kg) or saline control. (**b-d**) Baseline anxiety (**b**) and activity (**c**) assessed by open field and weight (**d**) of young Ftl1 OE or controls mice treated with NADH or saline. n = 10 young Ftl1 OE/NADH treated; n = 8 young Ftl1 OE/saline treated, N = 9 young control/NADH treated and N = 7 young control/saline treated. Data shown as mean±s.e.m.; two-way analysis of variance (ANOVA) with Tukey correction for multiple comparisons.

Consequently, we reasoned that boosting metabolic function might counter the pro-aging effects of increased neuronal FTL1. To begin to test this possibility, primary neurons were treated with NADH (a coenzyme driving ATP synthesis during oxidative phosphorylation)^21–23^ after viral-mediated FTL1 overexpression (Fig. 4r and Extended Data Fig. 9a). Consistent with our previous observation, we detected a decrease in the total neurite length and dendritic complexity of MAP2^+^ neurons overexpressing FTL1 compared to control (Fig. 4r); however, this decrease was mitigated after NADH treatment (Fig. 4r). No changes in neuronal toxicity were observed (Extended Data Fig. 9b). To investigate the effect of NADH supplementation on cognitive function, young adult mice were administered NADH after viral-mediated neuronal FTL1 overexpression, and hippocampal-dependent memory was assessed by NOR and Y maze (Fig. 4s). Although young mice with increased neuronal FTL1 expression showed no preference for either a novel object or a novel arm, these cognitive deficits were mitigated after NADH supplementation (Fig. 4t,u). No differences in overall activity were detected between experimental groups (Extended Data Fig. 10a–d). These transcriptomics and behavioral data indicate that the pro-aging effects of increased neuronal FTL1 on cognitive function are mediated, at least in part, through alterations in metabolic processes.

Cumulatively, our study elucidates a previously unrecognized role for neuronal FTL1 in the progression of aging-associated cognitive decline. Mimicking an age-related increase in neuronal FTL1 in young mice promotes synaptic changes and cognitive impairments indicative of hippocampal aging. Conversely, targeting the age-related increase in neuronal FTL1 reverses synaptic changes and restores hippocampal-dependent cognitive function in aged mice. Mechanistically, we implicate changes in metabolic processes, such as mitochondrial ATP production, as mediators of the pro-aging effects of neuronal FTL1 and demonstrate that boosting metabolic functions through NADH supplementation mitigates these pro-aging effects on cognition. Our findings provide a novel perspective on the molecular underpinnings of cognitive aging and identify neuronal FTL1 as a potential therapeutic target to counter age-related cognitive dysfunction.

FTL1 comprises the light chain of ferritin and is involved in the long-term storage of iron. Disruption in iron metabolism is known to promote mitochondrial dysfunction and subsequent alterations in metabolic processes^24^—a key hallmark of the aging process^25,26^. Age-related increased levels of neuronal FTL1 detected in the hippocampus are likely reflective of changes in brain iron metabolism during aging. This is congruent with our neuronal RNA-seq analysis pointing to altered metabolic processes, such as mitochondrial ATP synthesis, as mediators of the pro-aging effects of neuronal FTL1 on cognitive function. This link is further bolstered by recent reports that increasing metabolic function by supplementation with NAD^+^ and its precursors confers cognitive benefits in aged rodents^27–29^, similar to the effects that we observed with NADH in the context of increased neuronal FTL1.

Excess iron and its abnormal redox cycling can also lead to ferroptosis^30,31^, an iron-dependent programmed cell death pathway observed in many neurodegenerative diseases, such as Alzheimer’s disease^32,33^. Ferroptosis was previously characterized using Fe^2+^-specific and Fe^3+^-specific DNAzyme-based fluorescent turn-on sensors, triggering an increase in both Fe^2+^ or Fe^3+^ as well as a concomitant decrease in the Fe^3+^/Fe^2+^ ratio^20^. The observed decrease in the Fe^3+^/Fe^2+^ ratio resulting from ferroptosis is distinct from the increase observed after neuronal FTL1 overexpression in the hippocampus. These contrasting observations are likely reflective of key differences between normal aging in the absence of gross neuronal death and neurodegenerative disease conditions in which ferroptosis is observed.

Alterations in mitochondria dynamics are likewise widely reported in the brain during aging and age-related neurodegenerative diseases, such as Alzheimer’s disease, with structural effects to the mitochondrial inner membrane disrupting the electron transport system by modifying the available surface area necessary for ATP synthesis to occur^34,35^. Both bulk neuronal RNA-seq and single-nuclei RNA-seq after overexpression and abrogation of neuronal *Ftl1* point toward changes in the electron transport system linked to potential structural modifications in the mitochondrial inner membrane. Indeed, Gene Ontology analysis of bidirectional, differentially expressed genes indicates fluctuations in proton-motive force, implicating changes in electron transport through complexes I, II and IV that are coupled to electrogenic proton ejection across the mitochondrial inner membrane. These transcriptional analyses are further bolstered by in vitro Seahorse analysis, demonstrating altered ATP production after FTL1 manipulation in primary neurons. Collectively, this body of work posits changes in mitochondria dynamics, and structural alterations to the inner membrane, as potential downstream mechanisms regulating the pro-aging effects of increased neuronal FTL1.

From a translational perspective, aging is the most dominant risk factor for age-related neurodegenerative disorders^2,36,37^ such as Parkinson’s disease and Alzheimer’s disease. Of note, mutations in the *Ftl1* gene result in neuroferritinopathy—a rare inherited late-onset monogenic neurodegenerative disorder associated with movement disorders, dystonia and cognitive impairments^38–40^. Moreover, longitudinal studies of patients with Alzheimer’s disease showed that increased ferritin levels in cerebrospinal fluid negatively associated with cognitive performance over 7 years and predicted conversion from mild cognitive impairment to Alzheimer’s disease^41^. Thus, our data raise the exciting possibility that the beneficial effects of targeting neuronal FTL1 at old age may extend more broadly, beyond cognitive aging, to neurodegenerative disease conditions in older people.

## Methods

### Animal models

The following mouse lines were used: C57BL/6J mice (The Jackson Laboratory, line 000664), C57BL/6 aged mice (National Institutes of Aging) and B6;129-*Gt(ROSA)26Sor*^tm1(CAG-cas9*,-EGFP)Fezh^/J (The Jackson Laboratory, line 024857). All studies were done in young (2–3 months) or aged (18–22 months) male mice that were not involved in any previous procedures. The numbers of mice used to result in statistically significant differences were calculated using standard power calculations with *α* = 0.05 and a power of 0.8. We used an online tool (https://www.stat.uiowa.edu/~rlenth/Power/index.html) to calculate power and sample size based on experience with the respective tests, variability of the assays and inter-individual differences within groups. Mice were housed under specific pathogen-free conditions under a 12-hour light/dark cycle, with humidity maintained at 30–70% and temperature at 68–79 °F (20–26 °C). All animal handling and use were in accordance with institutional guidelines approved by the University of California, San Francisco (UCSF) Institutional Animal Care and Use Committee (IACUC).

### Tissue collection

Mice were anesthetized with 87.5 mg kg^−1^ ketamine and 12.5 mg kg^−1^ xylazine and transcardially perfused with ice-cold PBS. Tissues were removed and processed for subsequent analysis. To process the brains, either the hippocampus was subdissected and snap frozen or the whole brain was fixed in phosphate-buffered 4% paraformaldehyde (pH 7.4) at 4 °C for 48 hours before cryoprotection with 30% sucrose.

### Primary neuron cultures

Hippocampal and cortical neurons were isolated from E17 C57Bl/6J mouse embryos using the papain dissociation system (Worthington, cat. no. LK003153). Cells were plated at 100,000 cells per well on 12-mm poly-l-lysine-coated glass coverslips (Carolina, cat. no. 633009) in 24-well plates. Cultures were maintained at 37 °C and 5% CO_2_ in neurobasal medium (Thermo Fisher Scientific, cat. no. 21103049) supplemented with B-27 (cat. no. 17504044), GlutaMAX (cat. no. 35050061) and penicillin–streptomycin (cat. no. 15140122). Media were partially changed every 4–5 days. On day in vitro 8 (DIV8), neurons were infected with lentivirus at multiplicity of infection (MOI) = 1 and processed 10 days later (DIV18) for immunocytochemistry. Cells were fixed with 4% paraformaldehyde (10 minutes), washed and stained with MAP2 (Sigma-Aldrich, cat. no. M1406, RRID: AB_477171), anti-GFP (Aves Labs, cat. no. GFP-1020) or anti-turboGFP (Thermo Fisher Scientific, cat. no. PA5–22688, RRID: AB_2540616) antibodies to confirm infection, along with Hoechst 33342 (Thermo Fisher Scientific, cat. no. H3570) for nuclear staining.

### Viral plasmids and viruses

Lentiviral constructs for *Ftl1* overexpression, knockdown and conditional knockout were generated using standard molecular cloning techniques. For overexpression, murine *Ftl1* coding sequence (with partial untranslated regions) was polymerase chain reaction (PCR) amplified from adult mouse hippocampal cDNA and cloned into pENTR-D-TOPO (Thermo Fisher Scientific, cat. no. K240020), sequence verified and subcloned into a synapsin–IRES–eGFP lentiviral backbone using NheI and EcoRI. The synapsin–IRES–eGFP plasmid alone served as control. For knockdown, shRNAs targeting *Ftl1* (SH1–SH4) were cloned into the pGreenPuro system (System Biosciences), with a luciferase-targeting shRNA as control^15,42^. For CRISPR-based knockout vectors, six gRNAs targeting *Ftl1* exons 2–4 were designed using CHOPCHOP^43^ and cloned into LentiCRISPR v2 (ref. ^44^). gRNA sequences included CGTATTTGTAACCTCCCGCA and CGCAGACTGGCGCGCCCCAG, among others. A non-targeting gRNA (GGCGAGGGCGATGCCACCTA) served as control. Validated gRNA sequences were subcloned into a *Syn1*-Cre lentiviral vector (Addgene, no. 68841; ref. ^45^) using PacI and NheI/XbaI. All constructs were verified by Sanger sequencing. Lentivirus production followed previously described protocols^46^. *Ftl1* expression changes were confirmed by qRT–PCR and western blot in mouse cells.

### Lentivirus production

HEK293T cells (American Type Culture Collection, cat. no. CRL-11268) were transfected using a 4:3:1-µg ratio of lentiviral construct, psPAX2 (Addgene, no. 12260, gift from D. Trono) and pCMV-VSV-G (Addgene, no. 8454, RRID: Addgene_8454)^47^. After 48 hours, supernatants were cleared by centrifugation (5 minutes, 1,000*g*) and filtration (0.45 μm) and then concentrated by ultracentrifugation (24,000 r.p.m, 1.5 hours). Pellets were resuspended in PBS. Primary neurons were infected at MOI = 1. For in vivo injections, viral solutions were diluted to 1.0 × 10^8^ viral particles per milliliter (vp/ml).

### Confocal imaging and quantification

For hippocampal synaptic proteins, confocal images were acquired on an LSM 900 microscope (Zeiss) with a ×63 objective as *z* stacks (16 optical sections, 0.33-µm step size) per sample. Maximum intensity projections were generated for individual puncta analysis. Synapsin (555 nm) and gephyrin or PSD95 (647 nm) were quantified using the Analysis module. For co-localization, *z* stacks were split into four approximately 1-µm sub-stacks, each projected to two dimensions. Co-localization was assessed using the ZEN Toolkit 3D. For primary cell cultures, confocal *z* stacks (12 sections, 0.80-µm step) were acquired on an LSM 900 microscope (Zeiss) using a ×20 objective. Neurite length was quantified with NeuronJ (version 1.4.3) and imagescience.jar in FIJI (ImageJ version 1.54f). Sholl analysis was performed using the Simple Neurite Tracer in the Neuroanatomy plugin. Concentric rings (10-µm spacing, up to 300 µm) centered on the soma were used to quantify dendrite complexity by counting neurite intersections, which were summed to calculate total arborization and plotted as a function of distance.

### Real-time ATP rate using Seahorse

Mitochondrial ATP production was assessed in primary neurons (75,000 cells per well) using the Seahorse XFe24 Analyzer (Agilent Technologies). Cells were plated on Seahorse 24-well plates pre-coated with 50 μl of poly-l-lysine (10 µg ml^−1^; Millipore, cat. no. A-005-C) after *Ftl1* overexpression or knockdown. On the day of the assay, cells were equilibrated for 1 hour in Seahorse assay medium (DMEM supplemented with 10 mM glucose, 1 mM pyruvate and 2 mM glutamine; Agilent Technologies). ATP production was measured using sequential injections of 1.5 µM oligomycin and 0.5 µM rotenone/antimycin (Agilent Technologies, cat. no. 103592-100).

### Stereotaxic injections

Procedures were adapted from Lin et al.^46^. Mice were anesthetized with 2% isoflurane in oxygen (2 l min^−1^) and secured in a stereotaxic apparatus. Ophthalmic ointment was applied to prevent corneal drying, and fur over the skull was trimmed. Adeno-associated virus solutions (1.0 × 10^8^ vp/ml) were bilaterally injected into the hippocampal CA1 and dentate gyrus at the following coordinates (from bregma: AP: −2.0 mm, ML: ±1.5 mm; from skull surface: DV: −1.8 mm and −2.1 mm) using a 5-μl 26s-gauge Hamilton syringe. A volume of 2 μl per site was infused over 10 minutes (0.2 μl min^−1^). To minimize backflow, the needle was left in place for 8 minutes after injection and then partially retracted and held for another 2 minutes. Incisions were closed with silk sutures and VetBond. Mice received subcutaneous saline, enrofloxacin, carprofen and buprenorphine postoperatively and were monitored during recovery.

### Neuronal nuclei isolation

Neuronal nuclei were isolated from flash-frozen hippocampi following a modified version of Krishnaswami et al.^48^. Tissue was homogenized in 750 μl of ice-cold homogenization buffer (250 mM sucrose, 25 mM KCl, 5 mM MgCl_2_, 10 mM Tris (pH 8.0), 0.1% Triton X-100, 1 μM DTT, RNase and protease inhibitors) using a dounce homogenizer (Wheaton, cat. no. 357538; 12 loose and 20 tight pestle strokes). Homogenates were passed through a 40-μm filter and centrifuged (500 relative centrifugal force (RCF), 6 minutes, 4 °C) and then washed and pelleted again under the same conditions. Pellets were resuspended in staining buffer (0.5% BSA in PBS) and incubated on ice for 15 minutes. Alexa Fluor 488-conjugated anti-NeuN antibody (Millipore, cat. no. MAB377X, RRID: AB_2149209) was added at 1:250, and samples were rotated at 4 °C for 1 hour. After two washes in staining buffer, Hoechst 33342 was added (0.01 μg ml^−1^), and samples were filtered through 35-μm mesh FACS tubes prior to sorting.

### Nuclei isolation for single-nucleus RNA-seq

Neuronal nuclei were isolated from flash-frozen hippocampi using a modified version of the 10x Genomics protocol. Tissue was homogenized in 500 μl of NP-40 lysis buffer (10 mM Tris-HCl (pH 7.4), 10 mM NaCl, 3 mM MgCl_2_, 0.1% NP-40 substitute, 1 mM DTT and RNase inhibitor) with 20 loose and 25 tight pestle strokes (Wheaton, cat. no. 357538), followed by the addition of another 500 μl of buffer and 7-minute incubation on ice. Homogenates were filtered (40 μm) and centrifuged (500 RCF, 5 minutes, 4 °C) and then washed in PBS with 1% BSA and RNase inhibitor. After another spin, samples were resuspended in 400 μl of wash buffer containing Hoechst 33342 (1:10,000), incubated for 5 minutes on ice, filtered through 35-μm mesh and sorted using a BD FACSAria II.

### FACS

Nuclei were sorted on a BD FACSAria Fusion with a 70-μm nozzle and a flow rate of 1–2.5 ml min^−1^. Nuclei were first gated by forward and side scatter and then gated for doublets with height and width. Nuclei that were both Hoechst^+^ and NeuN^+^ (neuronal nuclei isolation) or only Hoechst^+^ (single-nucleus isolation) were sorted into TRI Reagent (Sigma-Aldrich, T9424) for RNA analysis.

### Library generation and RNA-seq

RNA-seq libraries were generated using a modified Smart-seq2 protocol (Picelli et al.^49^). After RNA isolation with TRI Reagent, 8 ng of high-quality RNA was reverse transcribed using SuperScript II (Thermo Fisher Scientific, cat. no. 18064-014) with anchored poly-dT and TSO primers. cDNA was amplified (10 PCR cycles) using KAPA HiFi HotStart polymerase (cat. no. KK2601) and purified with AMPure XP beads (Beckman Coulter, cat. no. A63881). Quality was assessed by Qubit fluorometry. For library construction, 2 ng of cDNA was fragmented (approximately 500 bp) with Nextera Tn5 transposase (Illumina, cat. no. FC-131-1096) and PCR amplified (12 cycles) with indexed Nextera primers (Illumina, cat. no. FC-131-1002). Final libraries were bead purified, analyzed with an Agilent Bioanalyzer and sequenced on an Illumina NovaSeq (paired-end 2 × 150 bp, SP flow cell).

### RNA-seq analysis

Reads were aligned to the *mm10* mouse transcriptome using STAR version 2.7.3a^50^ with ENCODE-recommended settings. Gene-level quantification was performed with RSEM version 1.3.1 (ref. ^51^), and differential expression analysis was carried out in R version 4.0.2 using DESeq2 version 1.28.1 (ref. ^52^). Full pipeline details (version 2.1.2) are available at https://github.com/emc2cube/Bioinformatics/. Gene Ontology enrichment was performed using Panther.

### Single-nuclei RNA-seq analysis

Nuclei were submitted to the UCSF IHG Genomics Core for processing with the 10x Genomics Chromium Single Cell 3′ kit. Approximately 10,000 nuclei per sample were captured, and cDNA libraries were prepared following manufacturer protocols (10x Genomics). Libraries were sequenced on an Illumina NovaSeq 6000 S2. Base calls were demultiplexed using Cell Ranger version 7.1, and raw FASTQ files were processed to generate expression matrices, including intronic reads. Each sample yielded approximately 13,100–15,950 nuclei, with a mean depth of 27,000 reads per nucleus and approximately 44% sequencing saturation. Data were analyzed in R version 4.2.2. Ambient RNA contamination was removed using SoupX, and samples were integrated with Seurat (2,000 variable genes, 20 dimensions). Nuclei expressing fewer than 300 or more than 5,000 genes, or more than 2% mitochondrial transcripts, were excluded. Genes detected in fewer than three nuclei were filtered out. Doublets and technical artifacts were removed, and expression was log normalized. Dimensionality reduction was performed using PCA, and clustering was conducted with the Louvain algorithm on the first 20 principal components. UMAP was applied for two-dimensional visualization. Differential expression was calculated using Seurat (min.pct = 0.05, log fold change > 0.15, pseudocount = 0.1). Genes inconsistent across replicates were excluded. Visualizations included violin plots, UMAPs and heatmaps from average expression matrices. Volcano plots were generated using EnhancedVolcano.

### Synaptosomes protein sample preparation

To obtain sufficient synaptosomes for proteomic analysis, hippocampus and cortex from multiple mice were pooled. Synaptosomes were isolated following a modified protocol from Trinidad et al.^53^. Tissue was homogenized in sucrose buffer with phosphatase inhibitors (Na_3_VO_4_, NaF, Na_2_MoO_4_, sodium tartrate, fenvalerate and okadaic acid) and PUGNAc, followed by differential centrifugation. Synaptic membranes were collected at the 1.0–1.2 M sucrose interface and pelleted. Frozen pellets were resuspended in 50 mM ammonium bicarbonate with 6 M guanidine hydrochloride, phosphatase inhibitor cocktails II/III and PUGNAc. Protein concentration was measured by BCA assay (Thermo Fisher Scientific). Each sample (1.5 mg) was reduced with 2.5 mM TCEP at 56 °C for 1 hour, alkylated with 5 mM iodoacetamide (45 minutes, room temperature, dark), diluted to 1 M guanidine and digested overnight at 37 °C with sequencing-grade trypsin (1:50 enzyme:substrate). Peptides were acidified with formic acid, desalted (C18 Sep-Pak) and dried by SpeedVac. Tryptic peptides were labeled with TMT-6plex reagents (Thermo Fisher Scientific) according to the manufacturer’s protocol: TMT126–131 for three young and three aged samples. Labeling efficiency was confirmed on a Q Exactive Plus Orbitrap. Labeled peptides were quenched with 5% hydroxylamine, pooled, desalted and dried. Peptides were separated using high-pH reverse-phase chromatography on a Gemini 5-μm C18 column (4.6 × 150 mm; Phenomenex). A 9–49% acetonitrile gradient in 20 mM ammonium formate (pH 10) was run over 20 ml at 550 μl min^−1^, collecting 60 fractions. Fractions were combined (two per sample) into 12 final samples and dried for downstream analysis.

### Mass spectrometry analysis

Tandem mass tag (TMT)-labeled peptides were analyzed on an Orbitrap Fusion Lumos mass spectrometer (Thermo Fisher Scientific) coupled to a NanoAcquity UPLC system (Waters). Peptides were separated on a 50-cm × 75-μm ID, 2-μm C18 EASY-Spray column using a linear gradient from 3.5% to 30% solvent B over 185 minutes. MS1 scans were acquired from 375 *m*/*z* to 1,500 *m*/*z* at 120,000 resolution (AGC target: 4.0 × 10^5^). Ions with charge states 2–7 were selected using a 1.0-*m*/*z* window, with dynamic exclusion (30 seconds) and MIPS filtering enabled. Higher-energy collisional dissociation MS2 spectra were acquired using stepped collision energies (30%/35%/40%) and detected in the Orbitrap at 30,000 resolution (AGC: 5.0 × 10^4^; maximum injection time: 100 ms). The scan cycle was set to 3 seconds. Peaklists were generated using Proteome Discoverer version 2.2 and searched against the SwissProt *Mus musculus* database (downloaded 6 September 2016, with decoy sequences) using Protein Prospector version 5.21.1. Searches assumed trypsin specificity with up to two missed cleavages. Fixed modifications included carbamidomethylation (Cys) and TMT-6plex labels (Lys and N termini); variable modifications included N-terminal acetylation, oxidation (Met), pyro-glutamate formation (Gln) and N-terminal Met loss (with or without acetylation). A maximum of two variable modifications per peptide was allowed. Identifications were filtered to a 1% false discovery rate at both peptide and protein levels.

### TMT mass spectrometry data analysis

Data were filtered to only include peptides unique to a single protein. Quantitation of TMT data was performed by calculating ratios of reporter ion peak intensities between conditions along with variance for each ratio and median normalized. Protein abundances were normalized by the median of ratio distributions. The age-dependent changes were determined using a normalized median log_2_ Aged/Young ratio of at least 1.0, corresponding to a 2.0 fold change with age and a −log_10_ *P* value greater than 1.3.

### RT–qPCR

To quantify mRNA expression levels, equal amounts of cDNA were synthesized using a High-Capacity cDNA Reverse Transcription Kit (Thermo Fisher Scientific, 4368813) and then mixed with SYBR Fast mix (Kapa Biosystems) and primers. β-actin was amplified as an internal control. RT–qPCR was performed in the CFX384 Real Time System (Bio-Rad). Each sample and primer set was run in triplicate, and relative expression levels were calculated using the 2^−ΔΔ*C*t^ method.

### Western blot analysis

Western blotting was performed on both mouse hippocampal tissue and primary hippocampal neurons. For tissue studies, hippocampi were dissected, flash frozen and pulverized prior to lysis in RIPA buffer (Abcam, ab156034) with protease inhibitors (Sigma-Aldrich, cat. no. 4693116001). Lysates were mixed with 4× LDS sample buffer (Invitrogen, NP0008), resolved by SDS-PAGE (Invitrogen) and transferred to nitrocellulose membranes. Membranes were blocked in 5% milk in TBST and probed with the following primary antibodies: anti-GAPDH (Abcam, ab8245, 1:5,000), β-tubulin (Covance, MMS-435P, 1:1,000), NR2A (Sigma-Aldrich, 07-632, 1:1,000), synapsin (Abcam, ab18814, 1:1,000) and AMPA receptor (Abcam, ab109450, 1:3,000). HRP-conjugated donkey anti-mouse (Invitrogen, A15999, 1:2,000) and anti-rabbit (GE Healthcare, NA934V, 1:2,000) secondaries were used for detection with ECL reagents (GE Healthcare). Signals were imaged with a ChemiDoc system (Bio-Rad) and quantified in FIJI/ImageJ (version 2.0.0).

For cell-based experiments, primary neurons were cultured from E17 mouse embryos, treated as indicated, lysed under the same conditions and processed in parallel. Quantification was normalized to loading controls (GAPDH, β-tubulin or β-actin) and to the control group. Each tissue datapoint represents an individual animal; each cell culture datapoint represents a biological replicate. Statistical comparisons were made using two-tailed unpaired Student’s *t*-tests.

### Iron quantification

Iron imaging was performed using a modified protocol from Wu et al.^20^. Mouse brains were fixed in 4% paraformaldehyde (24 hours, 4 °C), cryoprotected in 30% sucrose (3 days) and sectioned coronally at 30-μm thickness using a cryostat. Slices were stored at −20 °C in cryoprotectant. For each group, three sections from six mice per cohort were stained with technical replicates. Sections were rinsed in TBS (3×, 5 minutes), blocked in 2% BSA and incubated overnight at 4 °C with anti-GFP antibody (Aves Labs, cat. no. GFP-1020, 1:250). After washing, samples were incubated with Alexa Fluor 488-conjugated donkey anti-chicken secondary antibody (Jackson ImmunoResearch, cat. no. 703-545-155, 1:250) for 2 hours at room temperature. Excess antibodies were removed with TBS washes. To detect Fe^2+^ and Fe^3+^ simultaneously, DNAzyme-based sensors were used. Active and inactive enzyme (E/iE) strands (2 μM) and substrate strands (2.2 μM) were annealed in Bis-Tris buffer (5 mM Bis-Tris (pH 6), 40 mM sodium acetate, 200 mM NaCl) by heating to 95 °C and cooling to room temperature. Fe(II)-H5 and Fe(III)-B12 sensors were combined 1:1 with Hoechst. Brain slices were rinsed in Bis-Tris-acetate buffer, incubated in the sensor mix for 30 minutes and then washed and treated with 0.5× TrueBlack Lipofuscin Quencher (Biotium, PSF006) in 70% ethanol for 30 seconds. Slides were mounted with Fluoromount-G (SouthernBiotech) and imaged using a Nikon spinning disk or Zeiss LSM 710 confocal microscope with a ×20 objective. Channels used included 405 nm (Hoechst), 488 nm (GFP), 546 nm (Fe^2+^) and 647 nm (Fe^3+^). *z* stacks (four steps, 5-μm intervals) covering the hippocampus were acquired via tiled scan. Images were analyzed in ImageJ; maximum intensity projections were used. Background correction was done by subtracting the average signal from inactive sensor (iErS) controls in the same region (CA1, CA2/CA3 or dentate gyrus).

### DNAzyme sequences

**Fe(II)-H5**

- aE:/5IAbRQ/TGGATATCTCCTAGCCAGACTGTTATGTGTGATACGGCAAACTTCGTGATGCCTCTACGGGTCCG
- iE:/5IAbRQ/TGGATATCTCCTAGTCAGACTGTTATGTGTGATACGGCAAACTTCGTGATGCCTCTACGGGTCCG-3′
- rS:/5IAbRQ/CGGACCCGTATCAATCTCACGTATrAGGATATCCA/3AlexF546N/

**Fe(III)-B12**

- E: GCGGCATGCGCGTTTGCGGCACCTAAACGCTCCTAATAGAG/3IAbRQSp/
- iE:GCGGCATGCGCGTTTGCGGCACCTAAACGCCCCCTAATAGAG/3IAbRQSp/
- rS:/5Alex647N/CTCTATTArGGGAGACTCGCATGCCGC/3IAbRQSp/

### Electrophysiology

Acute hippocampal slices were prepared from 16–18-week-old male mice, 1 month after stereotaxic injection of *Ftl1*-overexpressing (Ftl1 OE) or control virus. All procedures were approved by the IACUC of AfaSci (protocol no. 0223). Mice (28–42 g) were deeply anesthetized with halothane and decapitated. Brains were rapidly removed and placed in ice-cold, oxygenated artificial cerebrospinal fluid (ACSF; continuously bubbled with 95% O_2_/5% CO_2_). ACSF composition (in mM) was as follows: 130 NaCl, 2.5 KCl, 1.2 KH_2_PO_4_, 2.4 CaCl_2_, 1.3 MgSO_4_, 26 NaHCO_3_ and 10 glucose (pH 7.4). Transverse hippocampal slices (400 μm) were prepared using a tissue slicer (Stoelting) and recovered for ≥1 hour at room temperature in oxygenated ACSF prior to recording. Field excitatory postsynaptic potentials (fEPSPs) were recorded in submerged slices perfused with oxygenated ACSF (flow rate: 1.75 ml min^−1^; DynaMAX pump) at room temperature. Glass microelectrodes (1–3 MΩ, ACSF-filled) were positioned in the stratum radiatum of CA1 to record responses evoked by stimulation of Schaffer collaterals via a concentric bipolar electrode (FHC, Inc.), placed approximately 100 μm apart. Stimuli were 0.2-ms biphasic pulses (0.4-ms total duration) delivered every 10 seconds. Input/output (I–O) curves were generated using 23 increasing current steps (20-µA increments) from threshold (20–100 µA). Test pulse intensity was set to 30–50% of the maximal response. LTP was induced using two trains of 100-Hz stimulation (100 pulses, 1-second duration, 5 seconds apart). fEPSP slope was measured from the initial negative phase using Clampfit 10.4 (Molecular Devices). Data points represent the average of three consecutive sweeps. Paired-pulse ratio (PPR) was calculated using a 50-ms interpulse interval as the ratio of the second to the first EPSP. Traces were low-pass filtered with a Gaussian filter (−3-dB cutoff: 1,260 Hz). Representative LTP traces are averages of six consecutive responses (1 minute, unfiltered). Recordings were completed within 8 hours of dissection. Data were analyzed using Clampfit 10.4, Excel and StatView 5.0. LTP was expressed as percent of baseline; the final 5 minutes were averaged for group comparisons. Results are shown as mean ± s.e.m. Statistical significance was defined as *P* < 0.05.

### Open field

Mice were placed in the center of an open 40-cm × 40-cm square chamber (Kinder Scientific) with no cues or stimuli and allowed to move freely for 10 minutes. Infrared photobeam breaks were recorded and movement metrics were analyzed by Motor Monitor software (Kinder Scientific).

### NOR

The NOR task was performed following White et al.^54^. Mice were habituated to an empty arena for 10 minutes (day 1) and then allowed to explore two identical objects for 5 minutes (day 2). On day 3, one object was replaced with a novel one, and exploration time over 5 minutes was recorded using Smart Video Tracking Software (Panlab, Harvard Apparatus). To control for object and spatial bias, object identity and novel object location were counterbalanced across animals. Objects were selected to elicit exploration regardless of genotype or age. The preference index was calculated as follows: (Time_novel / (Time_novel + Time_familiar)) × 100, where 100% indicates full novel object preference. Mice failing to explore both objects during training were excluded.

### Y maze

Spatial recognition was evaluated in a Y maze as described previously. During training, mice explored the start and trained arms for 5 minutes, with the third (novel) arm blocked. Maze arms were alternated and cleaned between trials. After a 45-minute delay, mice were reintroduced and allowed to explore all three arms for 5 minutes. Entries and time in each arm were tracked (Smart Video Tracking Software). Percent arm entries were computed from the first minute of exploration. The discrimination index was calculated as follows: (Time_novel − Time_trained) / (Time_novel + Time_trained). Mice making fewer than three entries in the first minute were excluded.

### Radial arm water maze

Spatial learning and memory was tested using an eight-arm radial arm water maze (RAWM) protocol^55^. Mice were trained to locate a constant goal arm, with the start arm varied between trials. Each incorrect entry was scored as an error. On day 1, mice underwent 12 training trials (blocks 1–4; alternating visible/hidden platforms), followed by three hidden-platform trials after a 1-hour break (block 5). On day 2, animals completed 15 hidden-platform trials (blocks 6–10). Errors were averaged per three-trial block. Experimenters were blinded to treatment and genotype during scoring.

### Mice NADH treatment

NADH (300 mg kg^−1^; Roche, cat. no. 10128023001) as well as vehicle (sodium chloride solution, Sigma-Aldrich, cat. no. S8776) were administrated intraperitoneally with an injection volume of 200 µl per mouse. All animals were treated with vehicle or NADH once daily for 9 days and for 2 hours pretrial.

### Primary cell culture NADH treatment

NADH (200 uM; Roche, cat. no. 10128023001) as well as vehicle (sodium chloride solution, Sigma-Aldrich, cat. no. S8776) were administrated for five consecutive days starting DIV14 until DIV18 included.

### Data, statistical analyses and reproducibility

All experiments were randomized and blinded by an independent researcher. Researchers remained blinded throughout histological, biochemical and behavioral assessments. Groups were unblinded at the end of each experiment on statistical analysis. Data are expressed as mean ± s.e.m. The distribution of data in each set of experiments was tested for normality using the D’Agostino–Pearson omnibus test or the Shapiro–Wilk test. Statistical analysis was performed using GraphPad Prism version 8.0, version 9.0 or version 10 (GraphPad Software). Means between two groups were compared using two-tailed unpaired Student’s *t*-tests. Additional statistical details are indicated in the respective figure legends. All data generated or analyzed in this study are included in this article. The main experimental findings are representative of two independently performed experiments. All replication attempts were successful. RNA-seq and proteomics data were not replicated due to resource limitations but were orthogonally validated.

Experimental replication was not attempted for negative data.

### Reporting summary

Further information on research design is available in the Nature Portfolio Reporting Summary linked to this article.

## Supplementary information

Supplementary Tables 1–6.

Reporting Summary

## Source data

Source Data Figs. 1–3 Unprocessed western blots for Figs. 1–3.

Source Data Extended Data Figs. 1–6 Unprocessed western blots for Extended Data Figs. 1–6.

## Data Availability

All data needed to understand and assess the conclusions of this study are included in the text, figures and supplementary materials. TMT mass spectrometry raw data files are available in the MassIVE repository (ID MSV000093450). RNA-seq and single-nucleus RNA-seq data are available in the Gene Expression Omnibus repository (GSE249105 and GSE249504).
