# Supplementary material for: Targeting iron-associated protein Ftl1 in the brain of old mice improves age-related cognitive impairment
Source: Nat Aging. 2025 Aug 19;5(10):1957–69. doi: 10.1038/s43587-025-00940-z (PMC12532579; doi:10.1038/s43587-025-00940-z)
Supplement: Supplementary file 1 — Supplementary Tables 1–6. [file 43587_2025_940_MOESM1_ESM.pdf]

nature aging

Supplementary information https://doi.org/10.1038/s43587-025-00940-z

Targeting iron-associated protein Ftl1 in the brain of old mice improves age-related cognitive impairment

In the format provided by the authors and unedited

Supplementary Table 1.

Gene	BaseMean	log2FoldChange	p-value	p-adj	Young1	Young2	Young3	Old1	Old2
Igsf8	4275.548942	0.826408907	0.00015667	0.02304699	2894.43139	3586.72426	3315.41218	5546.46649	6034.710394
Oaz1-ps	1668.060378	0.843554228	0.00053449	0.04807678	1115.87537	1392.06886	1289.42556	2361.10272	2181.829378
Rn45s	391067.8357	0.859409192	0.00051939	0.04758538	323735.565	340113.275	221108.53	552135.321	518246.4872
Kbtbd11	1027.943887	0.922683183	0.00023624	0.02952316	793.350057	737.913121	739.223326	1424.17288	1445.060047
Unc5a	1875.780523	0.965881655	5.2318E-05	0.01136658	1436.32657	1407.97216	1229.96822	2441.15685	2863.478809
Scap	1810.729618	0.970831954	0.00025331	0.03083878	1054.6889	1383.5871	1486.43346	2737.65363	2391.284999
Maneal	809.5752048	1.017677223	0.00015914	0.02316824	552.752393	601.144741	568.838117	1107.90965	1217.231127
Trim28	872.0390233	1.112568301	0.00013633	0.02092719	582.827101	635.071781	567.950695	1489.40217	1084.943367
Nlgn2	868.8714686	1.126846639	0.00028001	0.03295208	529.937097	504.66472	733.898788	1393.53488	1182.321857
Ftl1	2854.10927	1.151390478	4.3494E-07	0.00040948	1685.22071	1925.35952	2141.3516	4246.82226	4271.792257
Gdf1	930.7476912	1.173767568	0.00048037	0.04666658	463.565328	657.336401	737.44848	1145.46591	1649.922343
Aldh4a1	473.0271657	1.230537962	0.00019245	0.02586324	340.155319	260.81412	321.247112	712.580602	730.3386761
Lars2	1771.337406	1.230994829	9.4898E-08	0.00010579	1092.02302	1234.09608	1127.02716	2814.74279	2588.797974
Slc6a17	1435.465863	1.277310087	2.2871E-06	0.0015679	808.905941	825.911381	1108.39128	2277.09529	2157.025423
Rplp1	324.5080679	1.345452231	0.00048695	0.04666658	168.003542	224.76664	209.431819	569.273824	451.0645157
Rps19	323.2975932	1.422103547	0.00020338	0.02659433	214.671192	172.81586	192.570782	589.040276	447.3898557
Ercc2	437.2989165	1.444085318	0.00023086	0.02910934	302.821198	180.2374	293.737	788.681443	621.017541
Pcdhb9	401.7331184	1.543126483	0.00020025	0.02642929	246.820018	150.55124	284.86277	741.241958	585.1896059
Gsg1l	563.1653249	1.58785498	3.25E-05	0.00871014	332.895906	284.13896	320.359689	648.339632	1230.092437
Klhdc8b	199.3622268	1.65068954	0.00029364	0.0339904	118.224714	109.20266	94.9542567	288.590202	385.8393006
H6pd	286.4870108	1.709237161	0.00032814	0.03517001	194.967073	91.1789201	164.173248	561.367243	420.7485706
Abca7	253.3722411	1.80909926	0.00028317	0.0330492	96.4464776	142.06948	141.100251	596.946857	290.2981404
Dennd3	216.8142628	1.886655261	0.00030494	0.03445065	135.854716	61.4927601	115.364985	451.663433	319.6954205
Efcc1	218.8719926	1.967890165	0.00029679	0.03407519	159.70707	76.3358401	67.444145	441.780207	349.0927005
Zfp658	142.2396853	2.07850049	0.00021731	0.02789915	81.927653	47.7099	56.7950695	211.501039	313.2647655
Eya2	52.39590323	4.683212453	0.00015163	0.02278055	7.25941229	0	7.09938368	202.606135	45.01458507
Nynrin	21.86999631	5.334983375	0.00044655	0.0447248	0	2.12044	1.77484592	49.4161305	56.03856509
Srpx2	35.90459036	6.083608302	0.000366	0.03803503	2.0741178	0	1.77484592	2.96496783	172.7090203

Gene	BaseMean	log2FoldChange	p-value	p-adj	Young1	Young2	Young3	Old1	Old2
Ppp3r2	32.43763456	-3.963173947	0.00047562	0.04664403	70.5200051	37.1077	47.9208398	2.96496783	3.67466001
2310002F09Rik	37.65885355	-4.675811981	0.00030891	0.03462192	13.4817657	84.8176001	85.1926042	2.96496783	1.83733
Hkdc1	41.62372228	-4.121312748	0.00013051	0.0205222	84.0017708	82.6971601	33.7220725	4.94161305	2.755995
Zfp747	45.90180476	-3.960973915	0.00016309	0.02350173	58.0752983	41.34858	120.689523	2.96496783	6.43065501
Ccdc155	46.11087552	-4.510297718	0.00012526	0.02033259	30.0747081	122.98552	70.9938368	0.98832261	5.51199001
Adam21	49.27703389	-4.195203727	3.11E-05	0.008609	120.298832	61.4927601	55.9076465	5.92993566	2.755995
Rasa4	56.74466865	-3.996421381	0.00011269	0.01964741	91.2611831	27.56572	153.524172	4.94161305	6.43065501
Efemp1	59.1591551	-4.330950505	6.03E-06	0.00284067	47.7047093	141.00926	97.6165256	3.95329044	5.51199001
Gbe1	60.0911959	-3.52402866	0.00016719	0.02361089	143.114128	63.6132001	77.2057975	12.8481939	3.67466001
Arhgap25	65.89184647	-3.418328905	5.18E-05	0.01136658	112.002361	87.9982601	110.040447	14.8248392	4.59332501
Galnt3	72.3441172	-3.055772643	0.00012178	0.02016391	81.927653	86.9380401	165.948094	16.8014844	10.105315
Gabrg1	80.59469442	-3.70498739	0.00018539	0.02517378	117.187656	151.61146	114.477562	18.7781296	0.918665
Gjb6	88.02921535	-6.677973321	8.61E-09	2.43E-05	124.447068	248.09148	64.7818761	0.98832261	1.83733
Zfp81	90.20142872	-3.467559426	3.64E-05	0.00901349	52.8900038	134.64794	237.829353	11.8598713	13.779975
Bbs10	95.850145	-3.182913934	7.90E-05	0.01572276	66.3717695	230.06774	149.97448	22.73142	10.105315
Mob3a	97.63553499	-3.323427345	3.02E-05	0.00852629	108.891184	76.3358401	272.438849	15.8131618	14.69864
Zfp97	110.9326625	-3.930467919	5.80E-06	0.00282331	296.598845	171.75564	63.0070302	17.789807	5.51199001
Deptor	111.3086648	-3.76686575	9.74E-08	0.00010579	249.931195	131.46728	149.087057	17.789807	8.26798501
Dchs1	111.4227901	-2.922542772	2.62E-05	0.00770115	199.115309	124.04574	189.021091	11.8598713	33.0719401
Hist2h2be	112.7256809	-2.592646167	2.02E-05	0.00618643	212.597074	127.2264	167.722939	26.6847105	29.39728
Fdxr	118.920813	-2.117002408	0.00052229	0.04758538	210.522956	150.55124	154.411595	27.6730331	51.4452401
Rbm15	123.1382689	-2.519183859	0.00055487	0.04928183	246.820018	148.4308	156.186441	51.3927757	12.86131
Slc44a1	123.479759	-2.536180919	1.27E-05	0.00460218	140.002951	191.89982	221.85574	29.6496783	33.9906051
Wdr20	126.8955375	-2.868354099	0.00036629	0.03803503	179.411189	103.90156	298.174115	8.89490349	44.0959201
Zfp939	128.4693598	-3.34342047	7.43E-07	0.00063698	155.558835	276.71742	170.385208	28.6613557	11.02398
Rps6ka3	150.8229768	-1.766957322	0.00053303	0.04807678	209.485898	242.79038	178.372015	57.3227114	66.1438801
Acad11	152.7014396	-2.326963975	7.12E-05	0.01435993	255.116489	260.81412	157.961287	59.2993566	30.315945
Tmem55a	159.1364322	-2.212920934	3.75E-05	0.00913549	217.782369	247.03126	230.72997	65.2292923	34.9092701
1600014C10Rik	176.6686748	-2.530066436	7.16E-06	0.00306285	263.41296	336.08974	192.570782	30.6380009	60.6318901
Mgat5	179.4377034	-2.075564394	0.00012917	0.0205222	264.450019	243.8506	266.226888	84.9957445	37.6652651
Klf10	179.8037629	-2.974476197	2.84E-08	5.74E-05	183.559425	315.94556	329.233918	32.6146461	37.6652651
Gm5512	181.3282913	-6.708293459	2.59E-14	3.66E-10	157.632953	220.52576	522.692124	3.95329044	1.83733
Iqcc	182.94403	-2.164401682	8.37E-06	0.00330309	261.338842	227.9473	307.048344	50.4044531	67.9812101
Hsd3b7	204.9178822	-2.046813378	0.00051571	0.04758538	357.78532	213.10422	311.485459	36.5679366	105.646475
Zfp788	215.9724716	-2.570482552	0.00012279	0.02016391	434.527678	242.79038	293.737	18.7781296	90.0291701
Smim15	218.1182067	-1.785964493	3.41E-05	0.00876503	275.857667	315.94556	322.134535	82.0307767	94.6224951
Moap1	273.5944161	-2.962106743	1.54E-06	0.00121097	169.0406	595.84364	495.182012	71.1592279	36.7466001
Fcho2	282.7660598	-1.961732086	0.00031339	0.03484792	456.305915	190.8396	559.963888	117.610391	89.1105051
Hps5	282.7923979	-1.921286626	2.98E-05	0.00852629	428.305325	458.01504	315.922574	136.38852	75.3305301
Tbrg1	292.7158177	-1.912779088	7.87E-06	0.00326883	311.11767	525.86912	406.439716	112.668778	107.483805
Fam118b	297.5851163	-1.740193773	3.56E-05	0.0089725	381.637675	467.55702	391.353525	94.8789706	152.49839
Chm	314.6656176	-1.366815106	0.00021254	0.02753623	385.78591	430.44932	433.949828	153.190005	169.953025
Trib2	318.2605085	-1.502838141	0.00010781	0.01903062	402.378853	371.077	514.705317	156.154972	146.9864
Pms2	319.0641089	-1.507011191	0.00013079	0.0205222	488.454741	357.29414	446.373749	169.991489	133.206425
Tmem200a	334.6015683	-1.822913248	4.67E-06	0.00263784	429.342384	411.36536	567.063272	113.6571	151.579725
Cnot2	344.0409933	-1.437733953	0.00039985	0.04066389	578.678865	340.33062	461.459939	167.026521	172.70902
Zfp35	347.0174743	-2.282593457	6.37E-06	0.00290253	487.417682	714.588281	323.90938	139.353488	69.8185401
Tspyl3	353.1000492	-1.585644753	8.33E-05	0.0163464	386.822969	577.8199	480.095821	132.43523	188.326325
Ilk	365.3208417	-1.436407096	0.00032874	0.03517001	403.415912	550.25418	512.043048	141.330133	219.560935
Glce	386.4785961	-1.404206238	0.0004876	0.04666658	654.384165	375.31788	513.817894	212.489361	176.38368
Ankrd23	386.9827302	-2.025318664	5.69E-05	0.01165308	511.270037	629.770681	521.804701	67.2059375	204.862295
Ss18l1	393.3806996	-1.362942132	0.0003661	0.03803503	568.308276	542.83264	450.810864	245.104007	159.84771
Ppp1r15b	422.5960912	-1.259413315	0.00047238	0.04664403	457.342974	670.059041	525.354392	234.232459	225.99159
Dscr3	499.5149249	-1.743066918	4.99E-06	0.00270928	812.017118	719.889381	551.089658	160.108263	254.470205
Snupn	587.5738454	-1.175023906	0.00052183	0.04758538	674.088284	944.656021	649.593607	310.3333	359.198016
Zfp948	601.3860182	-1.536789616	5.51E-05	0.01162073	982.094777	552.37462	910.495957	322.193171	239.771565
Serp1	641.9963132	-1.182605066	0.00024232	0.03001754	890.833594	742.154001	848.37635	301.438396	427.179226
Ap3m1	664.7737491	-1.71934679	3.33E-05	0.00871014	781.942409	835.453361	1146.55046	384.457495	175.465015
Bhlhb9	713.072395	-1.417938305	0.00010244	0.01869502	1187.43244	806.827421	859.025425	444.745175	267.331515
Arpp19	715.9456155	-1.189689969	0.00032719	0.03517001	1176.02479	759.117521	835.065006	380.504205	429.016556
Galnt11	725.2481224	-1.406541386	2.58E-06	0.00165577	1033.94772	1034.77472	828.853045	393.352399	335.312726
Txndc16	728.7983043	-1.766573719	4.54E-05	0.01049966	874.240651	966.920641	1206.0078	165.049876	431.772551
Prpf39	801.4855063	-1.213661744	0.00010124	0.01869502	1272.47127	879.982601	960.191643	482.301434	412.480586
Pmpca	902.5721725	-1.458198462	1.65E-06	0.0012262	1160.46891	1181.08508	1290.31298	351.842849	529.151041
Ptcd3	913.7819809	-1.029431919	8.67E-05	0.01677643	1151.13538	1142.91716	1150.10016	525.787629	598.969581
Sgcb	928.8034895	-1.122160125	0.00026709	0.03200816	1123.13479	1354.96116	1077.33147	442.768529	645.821496
Gnl2	999.9229151	-1.043735977	9.55E-05	0.01798335	1346.10245	1162.00112	1269.90226	557.413952	664.194796
Slc25a51	1008.25602	-1.020928152	0.00032142	0.03517001	1381.36245	1102.6288	1310.72371	546.542404	700.022731
Usp33	1049.180719	-1.02115636	5.33E-05	0.01140424	1355.43598	1248.93916	1344.44578	624.61989	672.462781
Lrpprc	1219.915604	-1.257614471	5.58E-06	0.00282331	1565.95894	1349.66006	1853.82656	715.54557	614.586886
Ivns1abp	1336.312238	-1.622871597	1.33E-09	6.26E-06	1901.96602	1519.29526	2071.24519	603.865115	585.189606
Dgkb	1336.854473	-0.996789958	0.00011862	0.02016391	1572.18129	1881.8905	1556.53987	766.938346	906.722356
Tug1	1351.983161	-1.110697547	1.78E-05	0.00573834	1814.85307	1735.58014	1615.10979	697.755763	896.617041
Wdr47	1374.951298	-0.924991982	0.00038941	0.04014085	1795.14895	1452.5014	1840.51522	831.179315	955.411601
Neurod6	1486.669832	-1.111341311	0.00036145	0.03803503	1321.21304	2157.5477	2201.69636	822.284412	930.607646
Sphkap	1743.041567	-1.10632564	0.00026745	0.03200816	2892.35727	2105.59692	1656.81867	1027.85551	1032.57946
Zfp263	2140.992584	-1.093099185	5.60E-06	0.00282331	2851.91197	2380.1939	2924.05865	1179.06887	1369.72952
Cdh8	2150.649262	-0.817006587	0.00031641	0.03490854	2730.57608	2455.46952	2615.23546	1372.78011	1579.18514
Zfp207	2367.42114	-0.934350039	0.00024741	0.03038145	2924.50609	3091.60152	2759.88541	1266.04126	1795.07141
Plk2	3989.235544	-1.194227786	0.00015451	0.02296805	3338.29259	5535.40862	6572.25444	2041.87451	2458.34754
Ddx5	7031.914129	-0.843418624	0.00017585	0.02458811	8437.5112	7836.08603	9361.42481	4181.59296	5342.95565

Supplementary Table 1. RNA-seq analysis of neuronal nuclei from the hippocampus of young (3 months) and aged (18 months) mice. Differentially expressed genes in aged hippocampal neurons relative to young after filter application for BaseMean ≥10; p-adj. ≤ 0.05; Log_2FC ≥0.5 for the increased (top panel) and ≤ 0.5 for the decreased (bottom panel) genes.

Supplementary Table 2.

Protein Name	Median FC Y1	Median FC Y2	Median FC Y3	Median FC A1	Median FC A2	Median FC A3	t Test	-LOG10 P value	log2FoldChange
Hyaluronan and proteoglycan link protein 2	0.040026949	-0.055103997	0.019141285	2.095103693	2.050422915	1.784373108	4.06189E-05	4.391272234	1.976633238
Ig mu chain C region	-0.02048959	-0.12728621	0.118563058	1.483040305	2.127912478	1.207321232	0.004588454	2.338333623	1.606091338
Ig kappa chain C region	0.064625616	-0.117662129	0.039648154	1.154591165	1.637825352	0.925648391	0.004628442	2.334565182	1.239354969
Cytochrome b-245 heavy chain	-0.189634762	-0.324395645	0.405490821	1.178513771	1.099652852	0.931058342	0.009379078	2.027839832	1.069741655
Immunoglobulin J chain	0.006557875	0.088561283	-0.1064121	0.838247092	1.383754191	0.87141509	0.005049978	2.296710509	1.031138791
Ferritin light chain 1	-0.303875595	0.172981848	0.095913195	0.938493516	0.926142922	1.086813747	0.003134746	2.503797605	0.983816728
Serine protease HTRA1	0.061694631	-0.057349906	0.022282296	0.95496598	0.966300856	0.759499607	0.000306607	3.513418271	0.893588814
Complement C4-B	0.138406523	-0.299632743	0.083043635	0.785169826	0.898897525	0.754019836	0.004391361	2.357400836	0.812695729
Interferon-induced protein with tetratricopeptide repeats 3	-0.025624769	0.011498478	0.04517932	0.835481102	0.69017365	0.713276329	0.000119188	3.923765704	0.74631036
Myeloid differentiation primary response protein MyD88	0.011336323	0.010233592	-0.05589937	0.724136102	0.722532238	0.763407385	8.6015E-06	5.065425892	0.736691909
Lymphocyte-specific protein 1	-0.050996238	0.077380287	-0.03712809	0.817661887	0.670597326	0.680671998	0.000312665	3.504920315	0.72297707
Beta-1,4-mannosyl-glycoprotein 4-beta-N-acetylglucosaminyltransferase	0.293612505	-0.232543795	-0.11534493	0.594624448	0.762636198	0.653734254	0.014555917	1.836960421	0.670331633
Exocyst complex component 3-like protein 4	-0.153778904	0.036781393	0.104164181	0.560647635	0.645397025	0.795206891	0.002890184	2.539074493	0.66708385
Catenin alpha-3	-0.060160175	0.062964705	-0.0054461	0.627265142	0.76589753	0.524241062	0.001234787	2.908407975	0.639134578
Heat shock 70 kDa protein 1B	-0.132436275	0.258751907	-0.16607003	0.351156087	0.883971268	0.679623718	0.034391091	1.46355405	0.638250358
Protein S100-A4	-0.032077864	0.000502607	0.026232808	0.562260418	0.605803155	0.689527428	0.000110552	3.95643284	0.619197
Gap junction beta-6 protein	-0.237301672	0.076165375	0.127563479	0.453147771	0.475521798	0.836966831	0.023706938	1.625124528	0.588545467
Uncharacterized protein C19orf43 homolog	0.058211757	-0.002870427	-0.05803789	0.654958921	0.683195008	0.419216428	0.002879114	2.540741099	0.585790119
Ig gamma-2A chain C region secreted form	0.206789345	-0.194099226	-0.03641487	0.634819013	0.587682475	0.514309453	0.008524583	2.069326873	0.57893698
Laforin	-0.19927862	0.059922759	0.115421348	0.498101887	0.499253139	0.717719208	0.008807318	2.055156311	0.571691411
GATS-like protein 3	-0.167291867	0.098202668	0.055262598	0.511045798	0.560454086	0.640583692	0.003141489	2.502864488	0.570694525
Glutamate-rich protein 5	0.037670039	-0.042541298	0.014496105	0.552603027	0.616603048	0.540124342	7.2533E-05	4.139464613	0.569776805
Inter-alpha-trypsin inhibitor heavy chain H3	-0.130757972	0.034358991	0.087526439	0.596389383	0.561687486	0.543374226	0.001078385	2.967226056	0.567150365
Zinc finger CCCH-type antiviral protein 1	0.002442031	0.013421541	-0.01932444	0.563526059	0.593608312	0.538436784	6.96973E-06	5.156783812	0.565190385
Inter-alpha-trypsin inhibitor heavy chain H1	0.033346759	-0.095070663	0.057124708	0.619291839	0.477636708	0.559502949	0.00090279	3.044413296	0.552143832
Versican core protein	0.045679461	-0.04402252	-0.01354116	0.595134552	0.550470724	0.502509922	0.000123212	3.909347923	0.549371733
E3 ubiquitin-protein ligase RNF19A	-0.074329929	-0.085768409	0.147872364	0.575281252	0.571638768	0.464439344	0.003024556	2.519338374	0.537119788

Protein Name	Median FC Y1	Median FC Y2	Median FC Y3	Median FC A1	Median FC A2	Median FC A3	t Test	-LOG10 P value	log2FoldChange
Apolipoprotein M	0.005862201	-0.016845504	0.01083264	-0.545907627	-0.326923831	-0.637098763	0.00552591	2.257596464	-0.503310074
MARCKS-related protein	-0.114189727	0.042553789	0.012510629	-0.516453563	-0.553337613	-0.475663443	0.00072976	3.136822394	-0.51515154
Keratin, type II cytoskeletal 2 oral	0.216407675	-0.252328107	-0.086222366	-0.439590874	-0.673548255	-0.436202886	0.03959315	1.402379895	-0.516447339
Ubiquitin carboxyl-terminal hydrolase 43	-0.115465199	0.060148632	0.048713594	-0.548130553	-0.442223184	-0.568419126	0.00168407	2.773639124	-0.519590954
Protein FAM98A	0.064667274	0.098503268	-0.178707485	-0.402271024	-0.64114735	-0.592548868	0.00897723	2.04685772	-0.545322414
Tumor necrosis factor receptor superfamily member 19L	0.19836675	-0.228545307	-0.001289942	-0.539903018	-0.6060124	-0.528274636	0.01209101	1.917537513	-0.558063351
Alpha-1-antitrypsin 1-5	-0.052834628	0.057355648	-0.006647441	-0.524963954	-0.761466924	-0.440459748	0.00474554	2.323714524	-0.575630209
Insulin-degrading enzyme	-0.129703575	0.045431413	0.08872509	-0.576799721	-0.478510229	-0.702664911	0.00322482	2.491494477	-0.58599162
Mitogen-activated protein kinase 11	-0.146080677	0.034054338	0.100859734	-0.503540375	-0.72883001	-0.55259082	0.00418496	2.378308593	-0.594987069
Ras-related protein R-Ras2	0.093393625	-0.074106087	-0.035512208	-0.502478116	-0.623335609	-0.668191571	0.00111468	2.952849199	-0.598001766
Tetratricopeptide repeat protein 33	0.043830003	0.124033114	-0.185447885	-0.528103386	-0.643093471	-0.654906487	0.00398144	2.399959325	-0.608701114
Ryanodine receptor 1	-0.013224915	0.122938818	-0.073506487	-0.367769126	-1.033111019	-0.487407095	0.03940496	1.404449101	-0.62942908
Serine/threonine-protein kinase PLK4	0.007022932	0.187691541	-0.224013337	-0.435935209	-0.960393307	-0.536144446	0.03383754	1.470601231	-0.644157654
Plasma alpha-L-fucosidase	0.143217106	0.18234527	-0.394206149	-0.743400653	-0.446488987	-0.769023092	0.04155268	1.381401003	-0.652970911
High mobility group protein B1	-0.013209735	0.152152937	-0.15539263	-0.445708713	-0.498593635	-1.057530675	0.0369747	1.43209537	-0.667277674
Phospholipid phosphatase 4	-0.079756344	0.052323615	-0.053160806	-0.71837358	-0.72900112	-0.711564392	6.9515E-05	4.157920344	-0.719646364
GPI ethanolamine phosphate transferase 1	0.03232525	0.056566568	-0.093335379	-0.470758185	-0.716862244	-1.044308532	0.01260187	1.899564935	-0.74397632
Prominin-1	0.122397266	-0.087487502	-0.01087787	-0.793235709	-0.732374134	-0.719641322	0.000319	3.496205682	-0.748417055
Uncharacterized protein KIAA2013	0.220179358	0.159001998	-0.476724916	-1.058740957	-1.179520862	-1.2108707	0.00799222	2.097332498	-1.14971084

Supplementary Table 2. Proteomics analysis of hippocampal and cortical synaptosomes from young (3 months) and aged (18-20 months) mice. Differentially expressed proteins in the aged relative to young synaptosomes after filtering for p-nominal ≤ 0.05; $Log_2FC \geq 0.5$ for the increased (top panel) and ≤ 0.5 for the decreased (bottom panel) proteins.

Supplementary Table 3.

	Y maze				Novel Object		Watermaze				Average
ID	% Time - Novel	Z score	% Entries - Novel	Z score	% Time - Novel	Z score	Block 10 Errors	Z Score	Block 5	Z score	Z-cores
Young	39.1	-0.0494	41.30	0.1501	52.05	-0.6653	0.00	1.2561	1.67	0.5833	0.2549
Young	28.8	-0.8536	23.53	-2.0738	87.34	1.5244	2.67	-1.0029	3.33	-0.5914	-0.5995
Young	44.4	0.3718	36.36	-0.4680	51.83	-0.6789	1.00	0.4089	1.67	0.5833	0.0434
Young	43.3	0.2798	40.54	0.0546	57.61	-0.3202	0.67	0.6913	2.33	0.1134	0.1638
Young	53.4	1.0729	44.44	0.5430	40.68	-1.3707	1.00	0.4089	0.67	1.2882	0.3885
Young	21.2	-1.4495	27.27	-1.6055	86.41	1.4665	0.33	0.9737	3.33	-0.5914	-0.2413
Young	50.2	0.8208	41.67	0.1955	65.27	0.1550	1.00	0.4089	2.00	0.3484	0.3857
Young	65.6	2.0271	50.00	1.2381	66.67	0.2416	2.67	-1.0029	2.33	0.1134	0.5235
Young	39.1	-0.0443	46.67	0.8210	42.87	-1.2349	0.67	0.6913	1.67	0.5833	0.1633
Young	60.2	1.6035	55.56	1.9332	56.86	-0.3671	0.33	0.9737	0.67	1.2882	1.0863
Young	35.6	-0.3236	30.77	-1.1680	37.13	-1.5908	0.33	0.9737	2.67	-0.1215	-0.4460
Young	42.6	0.2294	40.74	0.0796	88.78	1.6134	0.67	0.6913	1.67	0.5833	0.6394
Young	47.7	0.6272	46.67	0.8210	58.50	-0.2653	0.67	0.6913	1.33	0.8183	0.5385
Young	48.5	0.6883	48.15	1.0064	60.83	-0.1205	0.00	1.2561	1.67	0.5833	0.6827
Young	38.5	-0.0949	38.46	-0.2056	66.36	0.2227	0.33	0.9737	0.67	1.2882	0.4368
Aged	44.8	0.3984	39.29	-0.1024	89.23	1.6416	2.67	-1.0029	6.00	-2.4710	-0.3073
Aged	34.1	-0.4348	37.50	-0.3259	55.41	-0.4571	0.67	0.6913	4.33	-1.2963	-0.3645
Aged	34.5	-0.4085	41.67	0.1955	44.28	-1.1475	2.67	-1.0029	5.67	-2.2361	-0.9199
Aged	37.1	-0.2039	46.67	0.8210	54.79	-0.4953	2.00	-0.4382	2.33	0.1134	-0.0406
Aged	45.0	0.4177	46.43	0.7913	71.94	0.5688	2.67	-1.0029	1.67	0.5833	0.2716
Aged	49.7	0.7828	41.67	0.1955	68.84	0.3765	4.00	-2.1324	4.33	-1.2963	-0.4148
Aged	25.4	-1.1240	29.03	-1.3853	70.08	0.4532	1.00	0.4089	1.67	0.5833	-0.2128
Aged	16.9	-1.7841	35.29	-0.6019	46.77	-0.9926	2.67	-1.0029	3.67	-0.8264	-1.0416
Aged	43.7	0.3163	44.44	0.5430	69.08	0.3916	3.00	-1.2853	1.67	0.5833	0.1098
Aged	39.4	-0.0202	34.78	-0.6659	62.34	-0.0270	2.67	-1.0029	4.33	-1.2963	-0.6024
Aged	11.5	-2.2118	33.33	-0.8472	54.46	-0.5160	1.67	-0.1558	3.33	-0.5914	-0.8644
Aged	15.0	-1.9347	25.00	-1.8898	43.06	-1.2230	3.67	-1.8500	3.00	-0.3565	-1.4508
Aged	46.2	0.5125	42.86	0.3444	100.00	2.3098	1.00	0.4089	1.00	1.0532	0.9258
Aged	49.8	0.7889	52.94	1.6061	70.94	0.5070	0.33	0.9737	1.67	0.5833	0.8918

Supplementary Table 3. Age-related changes in hippocampal FTL1 protein levels and cognitive performance. Young and aged mice were cognitively tested for hippocampus-dependent learning and memory by Y maze, Novel Object Recognition (NOR) and Radial Arm Water Maze (RAWM). The percent interaction time with the novel object, percent interaction time with the novel arm, and the number of errors committed in finding a hidden platform was quantified for each animal. A z-score was calculated for each behavioral test, and the average z-score across behavioral tests was used as a readout for overall cognitive performance. N=15 young and N=14 aged mice.

Supplementary Table 4.

Gene	baseMean	log2FoldChange	pvalue	padj	Control1	Control2	Control3	Control4	Control5	OE1	OE2	OE3	OE4	OE5	OE6	OE7
Dtx4	495.5201059	0.680976344	0.00025546	0.03875737	424.274899	279.050906	413.938458	373.240334	340.925679	734.019383	592.917335	490.456216	519.993252	585.372907	550.067873	641.98403
Bace1	1361.301128	0.756164827	1.42E-05	0.01167381	979.689313	741.267385	1112.50743	941.214756	1079.59798	2059.84741	1263.53553	1563.76228	1708.17345	1842.1057	1487.35951	1556.55279
Kansl3	824.6632287	0.778472252	0.00010721	0.02884962	783.531047	647.84065	527.178553	407.048335	542.698019	1046.70634	1252.77695	896.988213	943.529072	930.147628	822.475291	1095.03864
Fezf2	392.2310389	0.792630386	3.37E-05	0.01691645	250.156889	280.280205	309.879991	221.780488	310.775789	550.293713	539.124431	458.888391	519.993252	434.895691	473.523639	357.179987
Susd6	1385.795617	0.809856845	6.74E-06	0.01167381	1038.09599	1100.22274	1024.51681	952.033316	699.245525	1848.73955	1701.05114	1642.29687	1359.17292	1974.39337	1961.63359	1328.14558
4930402H24	836.3967156	0.84966725	0.00034902	0.04266775	678.839839	583.917095	642.714056	355.660173	586.763243	1354.97681	845.146282	915.46694	883.023955	701.95141	1167.67477	1320.62601
Efr3b	882.2405505	0.955635195	2.87E-05	0.01691645	812.183378	556.872513	537.890454	377.297294	564.730631	1570.50116	1028.04215	992.461637	798.842922	1119.48435	1127.90179	1100.67833
Ppp1r15b	680.935858	0.98351347	0.00027052	0.03875737	670.023737	518.76424	414.703593	260.99777	303.818122	841.781554	612.0437	933.945668	982.988931	708.565793	1159.42	764.177183
Ralgds	1533.270702	1.062018798	0.00011019	0.02884962	1785.26061	829.776924	883.731827	590.963862	599.518966	1632.33191	2313.09484	1903.3089	2214.13653	2030.61562	2052.43606	1564.07236
Pcdhga3	228.783055	1.364189325	1.50E-05	0.01167381	191.750214	103.261128	64.2714056	123.061125	114.801504	311.803661	328.734409	391.133058	347.246759	334.853146	259.650046	174.830204

Gene	baseMean	log2FoldChange	pvalue	padj	Control1	Control2	Control3	Control4	Control5	OE1	OE2	OE3	OE4	OE5	OE6	OE7
Snrpe	474.909703	-0.544259667	0.00054758	0.04824349	533.374159	506.471248	518.76206	682.921626	666.776413	442.531541	412.412259	383.433588	398.106133	381.153828	389.475069	383.498513
Ppdpf	847.264622	-0.650950216	0.00039727	0.04587923	919.078613	894.929779	1117.86338	1126.4826	1316.15866	875.346821	684.962969	580.540011	745.352891	750.732486	571.080015	584.647242
Ndufb3	1257.52701	-0.651146208	0.00045553	0.04706886	1430.41252	1252.65583	1312.20786	2223.21416	1761.44934	1067.02216	878.617421	977.062698	1115.39868	1166.61183	1007.08197	898.589652
Mrpl27	1147.03649	-0.693084901	0.00048764	0.04706886	1228.74419	1346.08257	1161.47612	1955.45479	1684.915	749.918719	1232.45519	782.266116	968.081873	945.856788	839.735265	869.451285
H2afv	824.288952	-0.710471614	0.00055841	0.04853616	1083.27851	760.936172	873.785062	1240.07749	1374.13922	695.154337	496.090109	667.544018	740.091577	551.474194	781.951874	626.944872
BC002163	2242.70164	-0.738823276	0.00010531	0.02884962	2854.21296	2152.50281	2640.48358	3925.78511	3062.53305	1445.07306	1796.68297	1479.06812	2119.43287	2183.57323	1615.68367	1637.38826
Atpif1	2689.26999	-0.768203747	0.000116	0.02884962	2782.58213	2665.12055	3189.08594	4627.63922	4447.10877	1862.8723	2570.10538	1623.81815	2337.77742	2011.59927	1898.59716	2254.93366
Cox17	386.714751	-0.77239913	0.00051852	0.04706886	414.356785	435.171898	470.558505	722.138907	507.909685	327.702997	372.96413	217.125044	278.84967	333.19955	270.156118	290.443726
Rps27a	1313.56964	-0.787976258	0.00010054	0.02884962	1464.57491	1268.63672	1575.41457	2609.9777	1786.96079	898.31253	1075.85807	1070.22628	1174.15003	1077.31765	859.996974	901.409494
Gng3	1915.79712	-0.81939973	0.00049638	0.04706886	2029.90744	1969.33723	1885.29456	3392.97101	3542.61207	1020.20745	1502.6151	1358.18644	1802.87711	1852.02728	1210.44949	1423.08026
Atp5h	4874.95858	-0.831586229	0.00046124	0.04706886	5972.90897	3492.43887	5646.70207	9460.83108	8169.46057	3377.72577	4151.61674	3003.56311	4396.70517	4423.36872	3266.63772	3137.5442
Rpl23a	910.594986	-0.831620694	0.00050354	0.04706886	994.015478	960.082633	1071.95523	1793.17639	1296.44527	535.277672	992.180217	590.549322	826.903267	724.274953	620.608637	521.670771
Cox6b1	5053.1755	-0.850159248	0.00016603	0.03084596	5787.77083	4032.1012	6030.03509	9479.76356	8801.44865	3451.92268	4706.28134	3028.97136	4116.97862	4424.19552	3276.39336	3502.24377
1810058I24Rik	307.436985	-0.855789894	4.57E-05	0.01894594	328.399792	374.93624	446.839296	486.835218	444.131071	243.789831	175.723484	203.265999	235.005382	260.441336	289.667393	200.208782
Cox6a1	3829.73643	-0.857681798	0.0004259	0.04607269	4368.37844	3161.7574	4157.74783	7967.86974	6271.17711	2252.40605	3457.09059	2484.61885	3393.54787	3546.96296	2389.38077	2505.89959
Rps26	523.051765	-0.858116148	9.77E-05	0.02884962	652.391533	496.636855	672.554352	979.079717	740.991526	309.153771	389.6997	328.767354	389.337275	467.140809	356.455988	494.412298
Rps25	1593.89637	-0.858212935	0.00023588	0.03875737	1661.83519	1443.1972	1746.80499	3131.97324	2808.57821	933.644389	1367.53514	998.621213	1417.92427	1202.99093	1083.6262	1330.02548
Cox7c	1540.01806	-0.869622923	0.00012063	0.02884962	1736.77205	1443.1972	1853.15886	3038.66316	2391.1182	1210.99949	1383.07532	796.125161	1198.70283	1179.84059	914.778631	1333.78527
Chchd1	478.504856	-0.876629251	0.00045137	0.04706886	444.111128	497.866154	560.079392	1080.50372	676.053302	431.048687	361.010151	290.270006	363.030702	317.49039	354.955121	365.639513
Sdf2l1	1225.7255	-0.886112486	0.00052874	0.04706886	1772.03646	1171.52209	1347.40411	2411.18665	1667.52084	646.57303	1018.47897	947.804713	1073.30816	1322.04983	718.915446	611.905715
Ndufc1	648.014413	-0.888602797	3.73E-08	0.00031893	833.12162	781.834257	848.535581	858.723233	1106.26904	450.481209	448.274195	422.700883	500.701766	558.088577	531.307031	436.135563
Atp5l	2247.64871	-0.889616342	0.00028834	0.03875737	2487.24272	1632.50927	2673.38442	4508.63505	4060.95826	1733.02771	2070.42908	1363.57607	1635.39193	1819.78216	1538.389	1448.45884
Rpl34	456.69087	-0.894396056	0.00034949	0.04266775	463.947357	442.547693	504.989616	874.951073	839.558474	370.984525	392.090496	273.331172	363.907588	349.735508	253.646577	350.600356
Snrpc	360.757174	-0.902954986	0.00051948	0.04706886	493.701701	387.229231	508.050159	766.765469	320.052678	201.3916	291.677076	264.091809	249.035554	356.349891	234.135302	256.605622
Coa3	2557.19462	-0.905679724	0.000403	0.04592105	2605.15808	2126.68752	2749.898	5247.0018	4834.41889	1861.989	1801.46456	1777.03759	2230.79736	2424.17142	1689.22617	1338.485
Snrpg	458.739482	-0.916108169	0.0005153	0.04706886	479.375535	478.197368	398.635742	1037.22948	767.662583	355.968485	352.642366	288.730112	319.186415	339.813933	300.173464	387.258302
Mrpl33	457.138265	-0.921682475	0.00024983	0.03875737	581.862719	432.7133	547.072084	1071.03748	521.825019	296.78762	417.19385	316.448202	330.585929	323.277976	285.915224	360.939777
Cox5b	4726.7424	-0.932120501	0.00029478	0.03875737	4865.38618	3558.82103	5413.33565	9571.72132	9307.03911	3027.94037	4321.36324	2900.39021	3924.94064	3977.72466	2988.22683	2864.01953
Ndufb4	1775.12557	-0.93304092	0.00012766	0.02884962	2073.98795	1427.21631	1928.14217	3218.52172	3642.33863	1268.41376	1433.28202	1092.55474	1461.76855	1560.16762	1130.15309	1064.96033
Tesc	1436.39049	-0.941315109	0.00041832	0.04607269	1633.18286	1660.78315	1280.07216	3140.08716	2255.44369	804.683102	1318.52383	1222.67578	982.988931	1405.55642	827.728327	704.960501
Ubl5	1032.05542	-0.945171747	3.85E-05	0.01729545	1228.74419	929.350155	1257.11809	2239.442	1516.77139	693.387744	757.882238	709.891101	936.513986	848.294637	631.865142	635.404398
Rpl28	1396.28588	-0.946066418	8.12E-05	0.02670112	1710.32375	1300.5985	1462.93961	2869.62315	2360.96831	877.99671	1111.72	952.424395	1132.05951	1326.18382	718.165012	932.427756
Cox8a	7647.64284	-0.953234202	0.00028619	0.03875737	8499.82415	6086.26007	8133.39335	16116.9504	14424.4031	4866.08036	7186.73189	4071.47955	6248.68789	6909.54998	4559.63492	4668.71841
2010107E04Rik	1305.54958	-0.957232032	8.27E-06	0.01167381	1438.12661	1234.21634	1655.75383	2480.15497	2294.87047	879.763303	1059.1225	747.618502	1050.50913	1056.64771	979.315926	790.495708
Ndufb2	1339.2052	-0.960318903	0.00052086	0.04706886	1538.40976	963.770531	1298.43542	2939.94379	2605.64626	881.529896	1124.86938	804.594578	1089.96899	1091.37322	753.435394	978.485175
Chchd10	3700.85371	-0.961335475	0.00043262	0.04621429	4951.34317	2390.98684	4854.0214	7651.42685	5987.07238	1928.23624	2758.97824	2091.9459	3637.32211	3545.30936	2146.24027	2467.36175
Mt3	3893.69552	-0.970988183	3.33E-05	0.01691645	4430.09115	3213.38797	4703.28965	6952.27738	7958.41134	2334.55262	2821.13893	2453.05103	3402.31673	3398.13933	2766.09847	2291.5916
Mif	3926.42095	-0.977012716	0.00016484	0.03084596	4900.65059	3603.0758	3659.64444	8132.85279	7238.29281	1956.50172	3013.59799	2647.07766	3575.06322	3721.41731	2143.23853	2525.63849
Ndufa1	1661.24265	-0.994648276	0.00051754	0.04706886	1736.77205	1241.59214	2570.09109	2751.9713	3408.09718	688.971262	1123.67398	862.3406	1691.51262	1463.43227	1311.75804	1084.69922
Tceb2	3248.06995	-0.997393972	0.00013841	0.02884962	4146.87388	2570.46452	3679.53797	7301.17596	5212.45213	2483.82973	2847.43768	1710.05221	2671.87089	2814.42002	1660.70969	1878.01477
Uqcr10	2679.65156	-1.013518828	3.27E-05	0.01691645	3198.04093	2186.92318	3074.31557	5083.37107	5445.53397	1699.46245	2144.54375	1398.22369	2307.08642	2191.84121	1669.71489	1756.76157
Rab33a	599.124696	-1.040272299	0.00017019	0.03094494	770.306895	575.312	527.943689	1448.33477	956.679201	481.396587	444.688001	374.964172	420.028276	501.86632	402.232442	285.74399
Ndufa3	1353.61471	-1.04331189	0.00015437	0.03032302	1542.81782	939.184548	1521.08993	2910.19275	2759.87454	952.193615	945.559701	663.694283	1054.89356	1135.19351	890.014321	928.667967
Rplp2	1311.39877	-1.044601693	0.00023719	0.03875737	1644.20299	1031.38198	1244.87592	3169.8382	2284.43397	882.413193	1169.0991	814.603888	982.112045	1124.44513	673.138993	716.239869
Cox7a2	2835.5024	-1.045836147	0.00012733	0.02884962	2982.04643	2109.47734	3175.31349	6278.822	5730.79832	1881.42152	2473.27816	1604.56947	2191.3375	2324.95567	1581.16372	1692.84515
Gpx1	1640.68168	-1.05349823	0.00025205	0.03875737	1778.64854	1274.78321	2025.31441	3783.7915	2895.54905	859.447484	1036.40994	917.776781	1576.64059	1620.52387	871.253479	1048.04128
Atox1	675.01153	-1.053989059	4.66E-05	0.01894594	879.406155	490.490359	1172.18802	1284.70405	1011.18092	442.531541	395.676689	418.851149	680.463346	508.480703	411.988079	404.177354
Rpl27	1330.40426	-1.058223429	0.00056226	0.04853616	1583.59229	925.662257	1228.80806	3273.96684	2534.90998	804.683102	1139.21415	744.538714	1100.49162	1173.22621	756.437129	699.320817
Rpl39	392.475781	-1.059317433	0.00028277	0.03875737	495.905726	295.031795	511.110702	923.634595	591.401688	365.684747	313.194237	221.744726	287.618527	272.016506	189.859716	242.506412
Mrpl54	683.777359	-1.059368747	0.00057644	0.04926295	709.696195	504.01265	635.827834	1538.94022	1520.25022	521.144929	511.630281	440.409664	552.438025	520.055874	417.241115	333.681304
Tomm6	608.215146	-1.06669835	8.10E-05	0.02670112	852.957849	516.305641	681.735981	1140.0058	1183.96299	310.920364	461.423571	422.700883	556.822454	530.804246	327.939509	313.002462
Hint1	3025.63028	-1.071469236	0.00039172	0.04587923	3471.34008	1894.34998	3136.29157	8115.27263	5174.18496	1970.63447	2427.85304	1790.89664	2405.29762	2505.19761	1524.13076	1892.11398
Tmem256	521.862212	-1.074767775	0.00027162	0.03875737	660.105622	347.891658	537.890454	1173.8138	1042.49043	401.899903	400.458281	278.720801	385.829732	368.75186	301.674331	362.819671
Tmem258	1086.21824	-1.077503814	0.00029477	0.03875737	1202.29588	812.566736	1035.22871	2655.95658	2130.20569	686.321372	1022.06516	641.365821	791.827836	810.261934	572.580882	673.942239
Sst	2859.76097	-1.080060183	0.00013	0.02884962	2456.38636	3381.80195	2518.06186	8165.30847	4124.73687	1878.77163	2370.47394	1707.74237	1930.02555	2191.84121	1818.30076	1773.68062
Uqcr11	4747.72778	-1.083574339	0.00013795	0.02884962	6004.86734	3214.61727	5096.56944	10258.6999	9733.77601	3580.00067	3892.21541	2304.45126	3931.07884	3857.01216	2477.93194	2621.51312
Naa38	1032.70036	-1.084735832	0.00047787	0.04706886	1081.07448	711.764206	1088.78822	2576.1697	2007.2869	588.275462	897.743787	517.40436	848.82541	837.546264	559.82351	677.702028
Rps23	1328.69532	-1.09543198	0.00015428	0.03032302	1323.51728	1353.45836	1440.75068	3363.21997	2152.2383	522.028225	1177.46688	768.40707	1146.08968	1059.9549	826.977893	810.234603
Fau	2108.78039	-1.106417774	0.00034852	0.04266775	2615.0762	1443.1972	2381.86769	5053.62003	3840.63214	934.527686	2025.00396	1122.58267	1757.27905	1866.08284	1186.43562	1079.05954
Snrpd2	1312.56625	-1.117998489	0.0004731	0.04706886	1532.8997	957.624035	1211.97508	3410.55117	2461.85448	688.087965	1257.55854	739.919033	1074.18505	1071.53007	686.646799	657.963134
Polr2i	648.642615	-1.118858882	0.00040949	0.04604582	698.676068	630.630462	565.435342	1474.02886	1364.86233	514.078557	369.377936	326.457513	518.239481	665.572303	330.19081	326.161725
Usmg5	2527.82161	-1.125600281	1.25E-05	0.01167381	2897.19145	2051.70028	2911.34165	5736.54166	4880.80334	1571.38445	2282.0145	1318.91915	1798.49268	1703.20366	1490.36125	1691.9052
Rpl35	1482.36896	-1.138172634	3.00E-05	0.01691645	1769.83244	1075.63675	1832.5002	3440.30221	2754.07649	949.543726	1340.04099	770.716911	1127.67508	1015.30781	821.724857	891.070073
Ndufs5	682.706363	-1.139045682	9.71E-06	0.01167381	856.263887	588.834291	866.89884	1505.13222	1192.08026	323.286515	469.791356	432.710194	564.714426	558.088577	461.516701	373.159092
Atp5k	891.004516	-1.143545236	0.00013602	0.02884962	886.018231	709.305607	1020.69113	2374.67401	1553.87894	508.778778	835.583099	498.155686	617.327571	646.555951	463.017568	578.067611
Polr2f	799.909845	-1.148531837	1.50E-05	0.01167381	1019.36177	656.445744	858.482346	1862.14471	1487.78111	575.026014	511.630281	493.536004	598.036085	653.997132	436.001956	446.474984
Tmsb10	221.535311	-1.165068329	7.96E-05	0.02670112	276.605194	285.197402	209.647204	490.892179	374.554402	89.2129454	174.528086	136.280613	141.178606	158.745195	126.072855	195.509046
Uqcrh	2001.62453	-1.170761661	0.00026013	0.03875737	2127.98657	1461.63669	2181.40211	5019.81203	4019.21225	792.316951	1648.45364	1210.35663	1905.47274	1585.79836	1060.36276	1006.6836
Siva1	617.004048	-1.173116956	0.00024465	0.03875737	642.473419	695.783317	570.791293	1448.33477	1211.79365	244.673128	319.171227	379.583853	536.654082	639.941568	407.485477	307.362778
Rps15	1834.21098	-1.173447792	0.00052419	0.04706886	1906.48201	1171.52209	1614.4365	4664.15186	4224.46343	1035.22349	1573.14357	1070.22628	1236.40891	1670.13174	844.237867	1000.10396
Atp5j2	2397.43323	-1.196063255	5.65E-05	0.02098501	2629.40236	1968.10793	2463.73722	6702.09817	4094.58698	1323.17814	1764.40723	1229.6053	2113.29467	1885.92599	1277.98852	1316.86622
Scand1	635.178681	-1.206888333	0.0001993	0.03475963	751.572678	560.560411	583.033465	1702.57094	1146.85543	406.316385	563.032388	306.438892	481.410279	496.905533	240.889205	382.558565
Rgs10	319.845747	-1.20778588	0.00028929	0.03875737	293.135385	499.095453	364.969768	613.953303	619.232355	98.9292068	174.528086	154.75934	281.480327	167.013174	306.927367	264.125201
Dpm3	461.041834	-1.214552208	0.00032623	0.04161074	489.29365	346.662359	451.430111	1311.75045	852.314197	318.870033	290.481678	211.735415	415.643848	306.742018	312.930836	224.647413
Rps21	579.776407	-1.224233279	0.00010771	0.02884962	710.798208	358.955351	667.963537	1399.65125	1212.95327	360.384968	462.618969	304.898998	431.427791	415.052542	264.152648	368.459355
Gm1673	458.980592	-1.237267467	0.0004223	0.04607269	563.128502	355.267453	390.984384	1292.81797	853.473808	347.13552	411.216861	217.125044	331.462815	325.758369	189.109282	230.287097
Rpl38	640.115615	-1.242635436	1.02E-05	0.01167381	808.87734	587.604992	766.666053	1639.0119	1023.93665	529.977894	469.791356	261.781968	432.304677	424.974116	318.183872	418.276564
Atp5e	815.761125	-1.248240119	1.18E-06	0.0042899	1003.93359	737.579488	970.192171	1798.58567	1648.96706	514.961853	622.802281	421.930936	650.64923	548.167002	446.508028	424.856195
Rpl36	1430.11289	-1.250718484	0.00024517	0.03875737	1530.69568	864.1973	1467.53043	4296.3208	2646.23265	982.225696	981.421637	683.712904	1140.82837	1100.46799	604.84953	862.871653
Rps20	555.826553	-1.28622635	0.00051765	0.04706886	508.027866	366.331146	605.222403	1705.27558	1052.92693	181.959077	442.297205	342.626399	423.535819	419.186531	322.686474	299.8432
Rpl41	3300.98657	-1.299257784	0.00011628	0.02884962	3624.51985	2351.64927	2847.83538	10082.8983	6341.91339	1907.03712	2824.72512	1604.56947	2582.42855	2327.43606	1500.11689	1616.70942
Uqcc2	1655.48424	-1.306750232	0.00030779	0.03985401	1914.1961	1071.94886	1326.74544	5077.96179	3295.6149	947.777133	1496.63811	793.045373	1239.91646	1231.10206	815.721388	655.143292
Ppp1r35	261.383202	-1.309789105	0.00039062	0.04587923	331.70583	190.541368	208.116932	686.978586	587.922854	214.641047	131.493764	138.590454	185.022894	209.179867	124.571988	127.832838
Rpl37	1101.13462	-1.321436152	0.00017733	0.03157297	1093.19662	837.152719	977.078393	3273.96684	2287.9128	506.128888	924.04254	456.57855	770.782578	892.941723	505.792287	688.041449
Bola2	337.840061	-1.328857241	3.77E-05	0.01729545	450.723205	344.203761	298.402955	920.929955	589.082465	209.341268	285.700087	140.130347	228.867182	248.866165	177.102344	160.730994
Rplp1	1856.37507	-1.336175919	0.00039377	0.04587923	2007.86718	951.477539	1789.65259	5952.91287	3628.4233	885.063082	1738.10848	985.532114	1270.60746	1423.74597	792.457945	850.652338
Rpl37a	2239.88705	-1.372758999	0.00015612	0.03032302	2330.75691	1376.81504	1905.1881	7716.33821	4117.7792	1400.02494	1874.38383	1005.55074	1420.55492	1514.69374	1030.34541	1186.21354
Rps29	1098.75615	-1.378827225	1.51E-06	0.0042899	1142.7872	1005.5667	1394.84253	2811.47339	2216.01691	587.392165	841.560088	457.348497	774.290121	746.598496	497.537516	709.660238
Rps28	298.006615	-1.445584015	5.38E-05	0.02091473	339.419919	249.547727	277.744289	841.143072	654.02069	154.576886	255.81514	120.881673	165.731408	217.447846	138.079793	161.670942
Rps27rt	1263.38067	-1.651554587	1.76E-05	0.01250615	1206.70393	858.050804	1182.13478	4780.45138	2460.69487	758.751684	491.308517	510.474837	851.456068	939.242405	491.534047	629.764714

Supplementary Table 4. RNA-seq analysis of neuronal nuclei from the hippocampus of young (3 months) following viral-mediated Ftl1 overexpression. Differentially expressed genes in Ftl1 OE hippocampal neurons relative to control after filter application for BaseMean $\geq$10; p-adj. $\leq$ 0.05; Log_2FC $\geq$0 for the increased (top panel) and $\leq$ 0 for the decreased (bottom panel) genes.

Supplementary Table 5

Gene	baseMean	log2FoldChange	pvalue	padj	control1	control2	control3	control4	shFtl1	shFtl3	shFtl4	shFtl5
Tuba1a	435.5990818	1.738263845	3.42E-24	1.69E-20	241.606537	216.60354	207.845209	141.290451	616.561799	686.342565	664.920557	709.621996
Tubb2b	88.34996422	2.013614797	8.84E-15	1.08E-11	42.4698991	28.2350984	59.9193395	19.228878	138.079159	131.67676	140.270912	146.919668
Tubb5	110.2053546	1.538043698	1.03E-14	1.08E-11	59.4578587	58.0836309	69.2817363	43.473985	145.750223	162.341211	156.666213	186.587978
Dpysl3	38.29091741	2.201869613	2.63E-13	2.17E-10	13.2128575	13.7141906	24.3422317	8.36038174	55.6152167	58.6232151	61.9378053	70.5214406
Eef1a1	168.6368877	1.309688895	2.43E-12	1.72E-09	93.433778	104.873222	123.583638	72.317302	237.802996	260.647833	240.464421	215.971912
Stmn2	62.91807829	1.708517639	3.32E-11	2.05E-08	45.3012257	26.2183056	31.8321491	18.8108589	102.600486	97.4047266	78.3331067	102.843768
Stmn1	52.49102238	1.575447686	6.49E-11	3.57E-08	31.1445927	23.3947958	26.214711	25.4991643	68.0806963	75.7592318	72.8680062	96.9669809
Tubb2a	172.9792308	1.12557002	1.95E-10	8.77E-08	113.253064	125.444508	110.476282	85.6939128	227.255282	220.964426	256.859722	243.886649
Actg1	242.8434902	1.256591884	5.28E-10	2.18E-07	146.285208	130.68817	202.227771	101.996657	271.363902	320.172944	388.022133	381.991137
Ftl1	80.91980501	1.488661738	8.09E-10	3.08E-07	49.0763278	45.5795159	56.1743808	24.245107	116.983732	106.423683	109.302009	139.573685
Tubb3	79.10693526	1.399788618	1.02E-09	3.59E-07	40.582348	31.8653253	67.409257	41.3838896	116.024849	102.8161	102.015209	130.758505
Atp5b	175.5781815	1.024866204	4.01E-09	1.24E-06	114.19684	129.478094	129.201076	91.9641991	223.41975	223.670113	253.216322	239.479059
Actb	273.2326181	0.989217637	7.04E-09	1.83E-06	178.373576	209.746445	200.355292	145.888661	352.868961	337.308961	327.906028	433.413021
Fabp7	22.45451918	2.48302509	8.58E-09	2.12E-06	14.156633	8.47052951	3.74495872	1.25405726	31.6431406	36.9777203	43.7208037	39.6683104
Dbi	22.77610162	2.024106012	1.09E-08	2.57E-06	11.3253064	6.05037822	13.1073555	8.36038174	36.4375558	35.1739291	29.1472025	42.6067037
Ptprz1	26.93044129	1.829944566	2.41E-08	5.19E-06	15.1004086	7.66381241	11.2348762	13.7946299	32.6020236	46.8985721	45.5425039	42.6067037
Ywhaz	143.9626322	0.999011752	2.35E-08	5.19E-06	94.3775535	92.772466	119.838679	83.1857983	174.516715	210.141679	178.526615	198.341552
Pdha1	16.12650651	2.283571263	3.80E-08	7.83E-06	6.60642874	5.24366112	7.48991744	4.18019087	32.6020236	21.6454948	21.8604019	29.3839336
Marcksl1	24.37622648	2.104517478	5.32E-08	1.05E-05	11.3253064	9.68060515	16.8523142	2.92613361	31.6431406	38.7815115	38.2557033	45.5450971
Mapre2	18.17953245	2.14813303	1.18E-07	2.15E-05	6.60642874	4.84030258	14.9798349	4.59820996	29.7253745	26.1549729	29.1472025	29.3839336
Dner	52.53869034	1.410425904	1.35E-07	2.31E-05	26.425715	28.6384569	46.811984	18.8108589	64.2451642	86.5819792	85.6199073	63.1754572
Hsp90aa1	64.15792535	1.245036959	1.50E-07	2.48E-05	45.3012257	39.1257791	41.1945459	28.0072788	80.546176	73.053545	92.9067079	113.128144
Gap43	26.99652578	1.848552816	2.13E-07	3.35E-05	22.6506128	12.504115	3.74495872	7.52434356	51.7796845	44.1928852	30.9689026	42.6067037
Acat1	17.51923004	2.101795325	2.71E-07	3.83E-05	8.49397981	4.84030258	9.3623968	5.43424813	23.0131931	19.8417036	30.9689026	38.1991137
Serpine2	24.92873476	1.67087269	3.21E-07	4.18E-05	15.1004086	12.1007564	9.3623968	10.4504772	33.5609066	32.4682422	36.4340031	49.9526871
Slc1a3	15.45291477	2.188990675	3.41E-07	4.32E-05	9.43775535	4.03358548	3.74495872	5.01622904	19.1776609	20.7435992	29.1472025	32.322327
Dynlt1b	12.3771318	2.860116086	5.10E-07	6.15E-05	3.77510214	0	3.74495872	5.01622904	18.2187779	17.1360167	36.4340031	14.6919668
Oxct1	35.75604339	1.497671662	8.84E-07	9.28E-05	19.8192862	13.7141906	33.7046285	13.7946299	59.4507489	44.1928852	45.5425039	55.8294738
Napb	15.01836411	2.110839346	1.45E-06	0.000137926	11.3253064	4.03358548	1.87247936	5.01622904	24.9309592	22.5473904	32.7906028	17.6303602
Ywhab	41.80242643	1.287860976	2.46E-06	0.000217155	26.425715	20.9746445	37.4495872	17.5568016	50.8208015	60.4270063	54.6510047	66.1138506
Mtpn	19.56150936	1.954126738	5.92E-06	0.000443862	15.1004086	3.22686838	13.1073555	4.59820996	32.6020236	23.449286	29.1472025	35.2607203
Map1b	96.7677199	1.029391958	6.11E-06	0.000444265	89.6586758	60.5037822	67.409257	41.3838896	127.531445	144.303299	111.12371	132.227701
Gstm1	22.46624942	1.609937853	6.35E-06	0.000455219	14.156633	14.1175492	11.2348762	5.85226722	39.3142049	32.4682422	27.3255023	35.2607203
Ywhaq	38.60098448	1.46104577	7.05E-06	0.000497827	19.8192862	24.2015129	33.7046285	8.77840082	53.6974506	49.6042589	45.5425039	73.459834
Slc6a1	43.78099514	1.213557646	7.42E-06	0.000517026	28.313266	23.3947958	37.4495872	19.6468971	50.8208015	48.7023633	72.8680062	69.052244
Hspd1	12.48377837	1.986620383	7.68E-06	0.000527386	5.66265321	5.64701967	1.87247936	5.43424813	18.2187779	19.8417036	18.2170016	24.9763436
Idh1	10.81018347	2.170058723	9.37E-06	0.000609635	6.60642874	2.42015129	3.74495872	3.76217178	18.2187779	16.2341211	16.3953014	19.0995568
Cct8	21.11593038	1.709910373	1.30E-05	0.00079654	14.156633	9.2772466	14.9798349	4.59820996	44.1086202	26.1549729	21.8604019	33.7915236
Phyhipl	16.47019551	1.726070244	1.39E-05	0.000827588	8.49397981	4.84030258	7.48991744	10.0324581	22.0543101	27.9587641	20.0387017	30.8531303
Ddah1	12.00549953	2.005318641	1.84E-05	0.0010219	3.77510214	6.45373677	5.61743808	3.3441527	23.9720762	12.6265386	18.2170016	22.0379502
Nrep	19.79558909	1.531366147	2.14E-05	0.001158717	15.1004086	7.66381241	11.2348762	8.36038174	25.8898423	29.7625554	30.9689026	29.3839336
Hspa8	254.2872367	0.894739838	2.38E-05	0.001250715	184.980005	214.183389	217.207606	99.4885427	319.308055	334.603274	351.58813	312.938893
Cs	28.3318209	1.291518945	2.91E-05	0.001478298	14.156633	19.7645688	22.4697523	11.2865153	41.231971	40.5853028	41.8991036	35.2607203
Slc25a3	54.05766698	1.065603992	3.84E-05	0.001867937	37.7510214	44.7727988	35.5771078	22.1550116	65.2040472	72.1516494	63.7595055	91.0901942
Stx7	10.85649348	2.127856951	4.72E-05	0.002222933	1.88755107	4.43694403	11.2348762	1.67207635	15.3421288	17.1360167	14.5736012	20.5687535
4833439L19Rik	15.93834145	1.581165249	5.07E-05	0.002344384	4.71887767	10.0839637	7.48991744	8.77840082	24.9309592	27.9587641	20.0387017	23.5071469
Cdc42	29.39400381	1.257701265	5.03E-05	0.002344384	11.3253064	17.7477761	28.0871904	15.0486871	34.5197897	42.389094	34.612303	51.4218838
Fdps	19.63991991	1.453920966	5.28E-05	0.002417526	11.3253064	9.2772466	9.3623968	11.2865153	23.9720762	27.9587641	41.8991036	22.0379502
L1cam	20.15109276	1.479223632	5.83E-05	0.002596245	13.2128575	11.6973979	9.3623968	7.94236265	26.8487253	20.7435992	27.3255023	44.0759004
Rac3	12.91244743	1.914040452	6.25E-05	0.002758851	5.66265321	4.03358548	13.1073555	2.92613361	22.0543101	22.5473904	10.9302009	22.0379502
Dpysl5	40.03811155	2.514504366	6.61E-05	0.002844336	17.9317352	11.2940393	18.7247936	1.67207635	49.8619184	61.328902	80.1548069	79.3366207
Ldha	43.34921928	0.992960294	7.14E-05	0.003012499	25.4819394	30.6552496	37.4495872	24.6631261	52.7385676	61.328902	60.1161051	54.3602772
Arf4	12.28916213	1.782580083	7.54E-05	0.00310718	5.66265321	4.43694403	1.87247936	8.36038174	18.2187779	18.0379123	18.2170016	23.5071469
Cdh13	11.01179637	1.910089122	7.62E-05	0.003114909	3.77510214	6.85709531	5.61743808	2.50811452	21.095427	15.3322255	18.2170016	14.6919668
Mapt	43.19473647	1.13403529	9.00E-05	0.003561666	38.6947969	14.9242663	35.5771078	23.827088	58.4918659	61.328902	51.0076044	61.7062606
Gpm6b	25.34236799	1.352676162	0.000101901	0.003906224	20.7630618	12.9074735	3.74495872	17.1387826	36.4375558	35.1739291	23.682102	52.8910805
Vdac1	36.21241677	1.101494795	0.000101705	0.003906224	17.9317352	27.4283813	31.8321491	17.1387826	41.231971	56.8194239	47.3642041	49.9526871
Atp5f1	19.67032331	1.514763849	0.000120847	0.004493156	7.55020428	16.941059	3.74495872	9.19641991	31.6431406	21.6454948	25.5038022	41.137507
Vcp	38.63923901	1.107377615	0.00013112	0.004767568	20.7630618	33.0754009	28.0871904	16.7207635	60.409632	53.2118414	60.1161051	36.729917
Mllt11	27.86184732	1.152141095	0.00014107	0.005055023	19.8192862	14.9242663	22.4697523	15.0486871	41.231971	40.5853028	29.1472025	39.6683104
Nnat	21.88982676	1.440988925	0.000147378	0.005205603	8.49397981	16.1343419	18.7247936	5.43424813	23.9720762	30.664451	36.4340031	35.2607203
Aco2	52.3801039	1.0283745	0.000178298	0.005959534	33.9759193	34.6888351	56.1743808	19.228878	74.7928777	68.5440669	72.8680062	58.7678672
Pla2g7	16.44465636	1.737028601	0.000181993	0.006039981	7.55020428	4.43694403	20.597273	3.3441527	24.9309592	23.449286	16.3953014	30.8531303
Slc25a5	23.95727017	1.238600684	0.00020542	0.006555968	9.43775535	16.1343419	20.597273	11.7045344	30.6842575	27.0568685	43.7208037	32.322327
Nudt19	10.40833016	1.791356885	0.000213532	0.006632938	4.71887767	4.43694403	5.61743808	4.18019087	10.5477135	15.3322255	16.3953014	22.0379502
Ppia	143.8531944	0.732707903	0.000215012	0.006632938	69.8393896	114.553828	140.435952	110.357039	165.886767	189.39808	176.704915	183.649585
Slc25a4	48.39158749	0.984719696	0.00021857	0.006632938	27.3694905	23.7981543	54.3019014	29.6793552	55.6152167	55.9175283	72.8680062	67.5830473
Fdft1	16.21343194	1.616940716	0.000237236	0.007025066	6.60642874	14.9242663	5.61743808	3.76217178	32.6020236	27.0568685	20.0387017	19.0995568
Gdi2	11.19560707	1.931931	0.000263985	0.00763394	4.71887767	7.66381241	3.74495872	1.67207635	15.3421288	8.11706055	21.8604019	26.4455402
Inpp5f	11.89408285	1.696269984	0.000280376	0.008060815	6.60642874	4.03358548	11.2348762	3.76217178	21.095427	18.0379123	12.7519011	17.6303602
Sept3	19.1003643	1.367334017	0.000321626	0.008682452	18.8755107	10.4873222	7.48991744	6.2702863	23.9720762	29.7625554	30.9689026	24.9763436
Tcaf1	12.55037802	1.696099675	0.000319898	0.008682452	12.269082	3.22686838	1.87247936	5.85226722	13.4243627	18.0379123	23.682102	22.0379502
Tspan3	43.33674728	0.913459328	0.000318724	0.008682452	30.2008171	28.2350984	33.7046285	29.2613361	48.9030354	55.9175283	45.5425039	74.9290307

Pgrmc1	23.50033895	1.31954653	0.000332709	0.008751316	19.8192862	8.87388805	20.597273	7.94236265	27.8076084	26.1549729	40.0774034	36.729917
Canx	16.75571554	1.416797695	0.00033586	0.00875887	10.3815309	6.85709531	13.1073555	8.36038174	34.5197897	18.0379123	23.682102	19.0995568
Atp5a1	86.36998579	0.807604	0.000381026	0.009813395	50.9638789	52.4366112	101.113885	54.3424813	93.0116556	119.952117	107.480309	111.658948
Gng2	10.5608101	1.741554214	0.000385768	0.009884043	6.60642874	2.82350984	7.48991744	4.18019087	15.3421288	14.4303299	21.8604019	11.7535734
Fabp5	14.43159071	1.503538228	0.000410961	0.010368386	11.3253064	6.85709531	5.61743808	5.85226722	13.4243627	17.1360167	27.3255023	27.9147369
Gdi1	51.74257294	0.867210843	0.000409209	0.010368386	28.313266	29.8485325	41.1945459	46.8181377	65.2040472	59.5251107	71.0463061	71.9906373
Gad1	23.62949611	1.37873292	0.000415312	0.010424952	16.9879596	19.7645688	11.2348762	4.18019087	27.8076084	33.3701378	41.8991036	33.7915236
Crmp1	76.74076331	1.561438109	0.000423115	0.010520312	28.313266	21.7813616	84.2615712	26.3352025	107.394901	109.12937	114.76711	121.943324
Hspa9	15.8888249	1.549350837	0.000425493	0.010520312	12.269082	8.06717096	11.2348762	3.3441527	28.7664914	21.6454948	10.9302009	30.8531303
Ndufa10	17.11595536	1.32787501	0.000516734	0.012404119	6.60642874	12.1007564	7.48991744	10.8684963	21.095427	21.6454948	21.8604019	35.2607203
Caprin1	10.42926787	1.876555825	0.000521914	0.012467956	9.43775535	2.01679274	5.61743808	2.50811452	14.3832457	9.92085179	14.5736012	24.9763436
Rps4x	14.03639191	1.520260667	0.000580885	0.013405501	10.3815309	7.26045386	11.2348762	2.92613361	23.0131931	23.449286	16.3953014	17.6303602
Pkia	12.6652098	1.58124458	0.000671187	0.015040346	6.60642874	2.42015129	7.48991744	9.614439	24.9309592	16.2341211	16.3953014	17.6303602
Rab1	13.22925099	1.437435064	0.000700238	0.015597642	7.55020428	7.26045386	9.3623968	5.43424813	13.4243627	22.5473904	18.2170016	22.0379502
Prdx6	17.80518368	1.291198169	0.000753307	0.016629921	9.43775535	11.2940393	14.9798349	7.10632448	23.0131931	19.8417036	20.0387017	36.729917
Sqle	18.81608098	1.216845002	0.000759085	0.016683012	15.1004086	8.06717096	13.1073555	10.8684963	27.8076084	26.1549729	20.0387017	29.3839336
Dpysl2	46.24197542	0.96244263	0.000814742	0.017224161	35.8634703	45.1761574	22.4697523	19.6468971	51.7796845	57.7213195	56.4727048	80.8058174
Cct6a	16.25667398	1.279273542	0.000835515	0.017369312	7.55020428	9.2772466	13.1073555	8.77840082	21.095427	21.6454948	30.9689026	17.6303602
Prdx1	15.68095944	1.437858225	0.00083405	0.017369312	3.77510214	9.2772466	18.7247936	5.43424813	18.2187779	29.7625554	18.2170016	22.0379502
Idh3a	13.83960252	1.353974924	0.000870389	0.017859227	9.43775535	6.45373677	7.48991744	7.94236265	17.2598948	18.939808	18.2170016	24.9763436
Tcp1	17.38523067	1.276171162	0.000957906	0.019255473	14.156633	8.47052951	11.2348762	8.77840082	35.4786727	24.3511817	14.5736012	22.0379502
Ncan	49.6008451	0.81479897	0.00108133	0.020794226	49.0763278	37.512345	28.0871904	29.2613361	73.8339946	58.6232151	52.8293045	67.5830473
Uqcrc2	19.04088578	1.16896739	0.001084916	0.020794226	12.269082	8.87388805	13.1073555	12.9585917	19.1776609	27.0568685	30.9689026	27.9147369
Cd200	12.77436739	1.429216199	0.001152745	0.021674244	8.49397981	5.64701967	7.48991744	6.68830539	12.4654796	20.7435992	12.7519011	27.9147369
Asns	24.64882965	1.129677832	0.001180647	0.022114772	23.5943884	18.1511347	5.61743808	12.9585917	44.1086202	29.7625554	21.8604019	41.137507
Rab11a	11.80420342	1.594067732	0.001275479	0.023447005	0.94377553	3.22686838	13.1073555	8.36038174	18.2187779	19.8417036	14.5736012	16.1611635
Dnaja1	17.30391535	1.234163395	0.001348656	0.024081824	13.2128575	8.47052951	14.9798349	7.10632448	26.8487253	18.0379123	21.8604019	27.9147369
Hspa5	26.98541379	0.983140813	0.001413488	0.024786166	20.7630618	16.1343419	14.9798349	20.4829353	40.273088	41.4871984	38.2557033	23.5071469
Cntn1	15.25915676	1.308867659	0.00145263	0.025204403	9.43775535	12.504115	3.74495872	7.52434356	26.8487253	16.2341211	16.3953014	29.3839336
Rps2	41.59050401	0.790291049	0.001499589	0.025748146	28.313266	28.6384569	37.4495872	30.0973743	54.6563337	57.7213195	47.3642041	48.4834904
Morf4l2	18.48525926	1.224468068	0.001511246	0.025769342	18.8755107	11.2940393	0	11.7045344	29.7253745	26.1549729	23.682102	26.4455402
Tmed2	11.0677752	1.476767899	0.001607355	0.026672377	8.49397981	4.84030258	5.61743808	4.59820996	13.4243627	11.724643	23.682102	16.1611635
Nars	18.46759973	1.113347162	0.001666975	0.027115756	11.3253064	9.68060515	13.1073555	12.9585917	21.095427	26.1549729	25.5038022	27.9147369
Atp5o	22.64772575	1.06885057	0.001795107	0.028653418	8.49397981	16.1343419	14.9798349	17.9748207	23.0131931	40.5853028	29.1472025	30.8531303
Mdh1	63.22054432	0.690893854	0.001805594	0.02870022	60.4016342	40.3358548	48.6844634	46.4001186	83.4228251	82.0725011	78.3331067	66.1138506
Rtn1	149.7335971	0.557958625	0.001933998	0.030004521	125.522146	115.763903	132.946035	112.447134	166.84565	163.243107	154.844513	226.256289
Rac1	20.93827705	1.042848432	0.002062391	0.031463104	16.9879596	11.6973979	13.1073555	12.9585917	22.0543101	27.0568685	32.7906028	30.8531303
Stx1a	11.7259965	1.453361939	0.002094668	0.031579672	5.66265321	4.43694403	1.87247936	11.2865153	23.0131931	15.3322255	14.5736012	17.6303602
Oaz2	17.17260913	1.228627775	0.002255857	0.03339884	8.49397981	12.9074735	16.8523142	5.01622904	19.1776609	28.8606597	25.5038022	20.5687535
Ccnd2	15.22394135	1.335951736	0.00242061	0.035102398	12.269082	9.2772466	11.2348762	3.3441527	14.3832457	20.7435992	18.2170016	32.322327
H3f3a	13.13105333	1.358013499	0.002475519	0.035379891	8.49397981	4.03358548	9.3623968	8.36038174	9.58883047	19.8417036	21.8604019	23.5071469
Csdc2	14.75219079	1.223157621	0.002590573	0.036601096	10.3815309	7.26045386	13.1073555	6.68830539	22.0543101	15.3322255	18.2170016	24.9763436
Stard7	17.30983635	1.219658457	0.002624326	0.036972341	13.2128575	10.0839637	7.48991744	9.19641991	12.4654796	18.0379123	40.0774034	27.9147369
Stxbp1	72.03763826	0.65428303	0.002675946	0.037274796	63.2329608	49.6131014	69.2817363	45.1460614	76.7106438	80.2687099	96.5501083	95.4977842
Usmg5	11.02014934	1.404070965	0.002727472	0.037885806	2.8313266	6.85709531	9.3623968	5.43424813	16.3010118	10.8227474	21.8604019	14.6919668
Abhd4	10.87083898	1.40844805	0.002748586	0.037965807	2.8313266	5.64701967	7.48991744	7.52434356	9.58883047	18.0379123	18.2170016	17.6303602
Dnajc5	24.86600936	1.004204426	0.002774406	0.038215704	14.156633	22.5880787	5.61743808	19.228878	28.7664914	32.4682422	36.4340031	39.6683104
Zfand5	10.60414595	1.628865499	0.002911826	0.039449266	5.66265321	2.82350984	13.1073555	2.50811452	7.67106438	15.3322255	12.7519011	24.9763436
Atp6v1h	10.6778887	1.45268493	0.003047074	0.040666594	6.60642874	4.84030258	11.2348762	2.92613361	19.1776609	11.724643	12.7519011	16.1611635
Rtn3	66.42408842	0.698170607	0.003103326	0.040928692	67.9518385	50.823177	33.7046285	46.4001186	68.0806963	69.4459625	96.5501083	98.4361776
Rpsa	42.52489643	0.725350536	0.003344749	0.043259745	31.1445927	30.2518911	35.5771078	32.1874697	44.1086202	58.6232151	51.0076044	57.2986705
Ldhb	44.66219154	0.805061052	0.003597014	0.045338476	22.6506128	41.5459304	22.4697523	38.8757751	53.6974506	62.2307976	74.6897064	41.137507
Rnf4	11.15721918	1.296224203	0.003612408	0.045338476	6.60642874	7.66381241	5.61743808	5.43424813	15.3421288	15.3322255	20.0387017	13.2227701
Mff	14.56184785	1.235547621	0.003632147	0.045470805	8.49397981	4.03358548	14.9798349	9.614439	18.2187779	19.8417036	23.682102	17.6303602
2900097C17Rik	30.0803899	0.974685847	0.003713514	0.045679914	35.8634703	16.941059	13.1073555	15.0486871	46.9852693	25.2530773	41.8991036	45.5450971
Serinc1	25.87768843	0.961171869	0.003845521	0.046608089	26.425715	15.3276248	18.7247936	11.7045344	35.4786727	27.9587641	27.3255023	44.0759004
Vps35	11.86228351	1.36821612	0.003869993	0.046790018	13.2128575	3.63022693	7.48991744	4.18019087	17.2598948	18.0379123	16.3953014	14.6919668
Zcchc12	16.14943656	1.169725044	0.004019899	0.04813982	12.269082	13.3108321	9.3623968	5.43424813	21.095427	27.0568685	12.7519011	27.9147369
Hsbp1	23.72139392	0.942847562	0.004074124	0.048364438	18.8755107	15.3276248	22.4697523	10.8684963	29.7253745	28.8606597	32.7906028	30.8531303
Atp6ap2	20.17771515	1.152174793	0.004223695	0.049493301	21.7068373	10.0839637	13.1073555	7.10632448	17.2598948	34.2720334	18.2170016	39.6683104

Supplementary Table 5. RNA-seq analysis of neuronal nuclei from the hippocampus of aged (18 months) following viral-mediated knockdown of Ftl1 (Ftl1 KD). Differentially expressed genes in Ftl1 KD hippocampal neurons relative to control after filter application for BaseMean ≥10; p-adj. ≤ 0.05; Log_2FC ≥0 for the increased (top panel) and ≤ 0 for the decreased (bottom panel) genes.

Supplementary Table 6.

Gene
Ftl1
Ndufa10
Atp5c1
Psmd8
Tmsb4x
Akr1a1
Rps6
Cisd1
Psmb1
Prdx2
Fdps
Sdhb
Rpl7
Pcp4
Atp5j
Atp5o
Rplp0
Ppia
Rps9
Dynlrb1
Atpif1
Chchd2
Cox4i1
Psmb5
Rps2
Stmn3
Gng3
Cox6b1
Cfl1
Rpl32
Uba52
Rps14

Supplementary Table 6. List of 32 shared genes across neuronal RNA-seq datasets from young mice overexpressing Ftl1 (Ftl1 OE), aged mice with hippocampal Ftl1 abrogation (Ftl1 KD), and single nucleus RNA-seq from aged Rosa26-fl-stop-fl-Cas9-eGFP transgenic mice with loss of neuronal Ftl1 (Ftl1 cKO). Down-regulated genes from Ftl1 OE RNA-seq analysis were compared to up-regulated genes from the Ftl1 KD and Ftl1 cKO RNA-seq analysis.
