# Supplementary material for: Targeting iron-associated protein Ftl1 in the brain of old mice improves age-related cognitive impairment
Source: Nat Aging. 2025 Aug 19;5(10):1957–69. doi: 10.1038/s43587-025-00940-z (PMC12532579; doi:10.1038/s43587-025-00940-z)
Supplement: Supplementary file 2 — Reporting Summary [file 43587_2025_940_MOESM2_ESM.pdf]

nature portfolio

Corresponding author(s): Saul Villeda and Laura Remesal Gomez

Last updated by author(s): Mar 14, 2025

Reporting Summary

Nature Portfolio wishes to improve the reproducibility of the work that we publish. This form provides structure for consistency and transparency in reporting. For further information on Nature Portfolio policies, see our Editorial Policies and the Editorial Policy Checklist.

Statistics

For all statistical analyses, confirm that the following items are present in the figure legend, table legend, main text, or Methods section.

n/a	Confirmed	
☐	☒	The exact sample size (*n*) for each experimental group/condition, given as a discrete number and unit of measurement
☐	☒	A statement on whether measurements were taken from distinct samples or whether the same sample was measured repeatedly
☐	☒	The statistical test(s) used AND whether they are one- or two-sided<br>*Only common tests should be described solely by name; describe more complex techniques in the Methods section.*
☐	☒	A description of all covariates tested
☐	☒	A description of any assumptions or corrections, such as tests of normality and adjustment for multiple comparisons
☐	☒	A full description of the statistical parameters including central tendency (e.g. means) or other basic estimates (e.g. regression coefficient) AND variation (e.g. standard deviation) or associated estimates of uncertainty (e.g. confidence intervals)
☐	☒	For null hypothesis testing, the test statistic (e.g. *F*, *t*, *r*) with confidence intervals, effect sizes, degrees of freedom and *P* value noted<br>*Give P values as exact values whenever suitable.*
☒	☐	For Bayesian analysis, information on the choice of priors and Markov chain Monte Carlo settings
☒	☐	For hierarchical and complex designs, identification of the appropriate level for tests and full reporting of outcomes
☐	☒	Estimates of effect sizes (e.g. Cohen's *d*, Pearson's *r*), indicating how they were calculated

Our web collection on statistics for biologists contains articles on many of the points above.

Software and code

Policy information about availability of computer code

Data collection	Illumina HiSEq 2500 (paired reads 2 × 100 bp); CFX384 Real Time System (Bio-Rad); Zeiss LSM800 confocal microscope; Zeiss LSM900 confocal microscope; ChemiDoc System (BioRad); Cytation 5 (BioTek); NovaSeq 6000 S2; BD FACSAria II, Smart Video Tracking Software (Panlab; Harvard Apparatus); 10x Chromium (10x Genomics); Orbitrap Fusion Lumos (Thermo Scientific) equipped with a NanoAcquity UPLC (Waters), FreezeScan video tracking system (Cleversys, Inc);Seahorse XFe24 Analyzer
Data analysis	Statistical analysis was performed with Prism 8.0 or 9.0 or 10 software (GraphPad Software).<br>ImageJ software (Version 2.0.0);<br>For bulk RNA-sequencing alignment of sequencing reads to the mouse mm10 transcriptome was performed using STAR v2.7.3a39 following ENCODE standard options, read counts were generated using RSEM v1.3.1, and differential expression analysis was performed in R v3.6.1 using the DESeq2 package v1.38.040 (detailed pipeline v2.0.1 and options available on https://github.com/emc2cube/Bioinformatics/).<br>For single nucleus RNA sequencing, fastq files were converted to count matrices using CellRanger 7.0.1 (10x Genomics). Data was further processed using Seurat v4 (Satija lab).<br>For mass spectrometry data was analyzed using Proteome Discoverer 2.2 and Protein Prospector (v5.21.1)

For manuscripts utilizing custom algorithms or software that are central to the research but not yet described in published literature, software must be made available to editors and reviewers. We strongly encourage code deposition in a community repository (e.g. GitHub). See the Nature Portfolio guidelines for submitting code & software for further information.

Data

Policy information about availability of data

All manuscripts must include a data availability statement. This statement should provide the following information, where applicable:

- Accession codes, unique identifiers, or web links for publicly available datasets
- A description of any restrictions on data availability
- For clinical datasets or third party data, please ensure that the statement adheres to our policy

All data needed to understand and assess the conclusions of this study are included in the text, figures, and supplementary materials. All bulk RNA-sequencing data that support the findings of this study are available in GEO with the accession number GSE249105 and GSE249504. All proteomic data that support the findings of this study are available in MassIVE with dataset ID: MSV000093450.

Research involving human participants, their data, or biological material

Policy information about studies with human participants or human data. See also policy information about sex, gender (identity/presentation), and sexual orientation and race, ethnicity and racism.

Reporting on sex and gender	no human data was used in this study
Reporting on race, ethnicity, or other socially relevant groupings	no human data was used in this study
Population characteristics	no human data was used in this study
Recruitment	no human data was used in this study
Ethics oversight	no human data was used in this study

Note that full information on the approval of the study protocol must also be provided in the manuscript.

Field-specific reporting

Please select the one below that is the best fit for your research. If you are not sure, read the appropriate sections before making your selection.

☒ Life sciences ☐ Behavioural & social sciences ☐ Ecological, evolutionary & environmental sciences

For a reference copy of the document with all sections, see nature.com/documents/nr-reporting-summary-flat.pdf

Life sciences study design

All studies must disclose on these points even when the disclosure is negative.

Sample size	The numbers of samples used were found to be sufficient to result in statistically significant differences using standard power calculations with alpha = 0.05 and a power of 0.8. We use an online tool (http://www.bu.edu/orccommittees/iacuc/policies-and-guidelines/sample-sizecalculations/) to calculate power and sample size based on experience with the respective tests, variability of the assays, and inter-individual differences between groups.
Data exclusions	All exclusion criteria were pre-established. For novel object recognition, mice that did not explore both objects during the training phase were excluded from analysis.
Replication	Results shown are representative of several independently performed experiments.
Randomization	All experiments were randomized and blinded by an independent researcher
Blinding	Researchers remained blinded throughout histological, biochemical and behavioral assessments. Groups were un-blinded at the end of each experiment upon statistical analysis.

Reporting for specific materials, systems and methods

We require information from authors about some types of materials, experimental systems and methods used in many studies. Here, indicate whether each material, system or method listed is relevant to your study. If you are not sure if a list item applies to your research, read the appropriate section before selecting a response.

Materials & experimental systems

n/a	Involved in the study	
☐	☒	Antibodies
☐	☒	Eukaryotic cell lines
☒	☐	Palaeontology and archaeology
☐	☒	Animals and other organisms
☒	☐	Clinical data
☒	☐	Dual use research of concern
☒	☐	Plants

Methods

n/a	Involved in the study	
☒	☐	ChIP-seq
☐	☒	Flow cytometry
☒	☐	MRI-based neuroimaging

Antibodies

Antibodies used	For immunohistochemistry: anti-MAP2 antibody 1:500 (Sigma-Aldrich, Cat# M1406, RRID:AB_477171), anti-GFP antibody 1:500 (Aves labs, Cat# GFP-1020), turbo-GFP antibody 1:500 (Thermo Fisher, Cat# PA5–22688, RRID:AB_2540616), AlexaFluor 555 Donkey anti-mouse 1:500 (Invitrogen Cat# A31570), AlexaFluor 488 Donkey anti-rabbit 1:500 (Invitrogen Cat# A21206)<br>For neuronal nuclei isolation: anti-NeuN-AlexaFluor488 antibody 1:250 (Millipore, Cat#MAB377X, RRID:AB_2149209)<br>For Western blot analysis: anti-GAPDH 1:5000 (Abcam, Cat# ab8245), anti-B-tubulin 1:1000 (Covance, Cat#MMS-435P), anti-NR2A 1:1000 (Sigma Millipore, Cat# 07-632,), anti-Synapsin 1:1000 (Abcam, ab18814,), anti-AMPA (Abcam. Cat#ab109450, 1:3000), anti-Ftl1 1:1000 (Abcam Cat# ab69090), anti-mouse HRP-linked 1:5000 (Cell signaling Cat#7076S) and anti-rabbit HRP-linked 1:5000 (Cell signaling Cat#7074S).
Validation	All antibodies are from commercially available sources and have been validated by the supplier. Manufacturer's website contains validation and publications supporting the antibodies use for each assay.

Eukaryotic cell lines

Policy information about cell lines and Sex and Gender in Research

Cell line source(s)	American Type Culture Collection (ATCC)
Authentication	Authentication was performed by ATCC prior to procurement.
Mycoplasma contamination	All cell lines tested negative for mycoplasma.
Commonly misidentified lines<br>(See ICLAC register)	*Name any commonly misidentified cell lines used in the study and provide a rationale for their use.*

Animals and other research organisms

Policy information about studies involving animals; ARRIVE guidelines recommended for reporting animal research, and Sex and Gender in Research

Laboratory animals	The C57BL/6 mouse line was used for all experiments (The Jackson Laboratory and National Institutes of Aging). All studies were performed with either young (2.5-3 months), or aged (18-24 months) male mice.
Wild animals	no wild animals were used in the study.
Reporting on sex	findings are specific to male mice
Field-collected samples	no field collected samples were used in the study.
Ethics oversight	This study was approved by the University of California San Francisco Institutional Animal Care and Use Committee (IACUC)

Note that full information on the approval of the study protocol must also be provided in the manuscript.

Plants

Seed stocks | no seeds were used in this study

Novel plant genotypes | no plants were used in this study

Authentication | *Describe any authentication procedures for each seed stock used or novel genotype generated. Describe any experiments used to assess the effect of a mutation and, where applicable, how potential secondary effects (e.g. second site T-DNA insertions, mosiacism, off-target gene editing) were examined.*

Flow Cytometry

Plots

Confirm that:

- [ ] The axis labels state the marker and fluorochrome used (e.g. CD4-FITC).
- [ ] The axis scales are clearly visible. Include numbers along axes only for bottom left plot of group (a 'group' is an analysis of identical markers).
- [ ] All plots are contour plots with outliers or pseudocolor plots.
- [ ] A numerical value for number of cells or percentage (with statistics) is provided.

Methodology

Sample preparation | *Describe the sample preparation, detailing the biological source of the cells and any tissue processing steps used.*

Instrument | *Identify the instrument used for data collection, specifying make and model number.*

Software | *Describe the software used to collect and analyze the flow cytometry data. For custom code that has been deposited into a community repository, provide accession details.*

Cell population abundance | *Describe the abundance of the relevant cell populations within post-sort fractions, providing details on the purity of the samples and how it was determined.*

Gating strategy | *Describe the gating strategy used for all relevant experiments, specifying the preliminary FSC/SSC gates of the starting cell population, indicating where boundaries between "positive" and "negative" staining cell populations are defined.*

- [ ] Tick this box to confirm that a figure exemplifying the gating strategy is provided in the Supplementary Information.
