# Supplementary figures and images for: Targeting iron-associated protein Ftl1 in the brain of old mice improves age-related cognitive impairment
Source: Nat Aging. 2025 Aug 19;5(10):1957–69. doi: 10.1038/s43587-025-00940-z (PMC12532579; doi:10.1038/s43587-025-00940-z)

Figure 1g

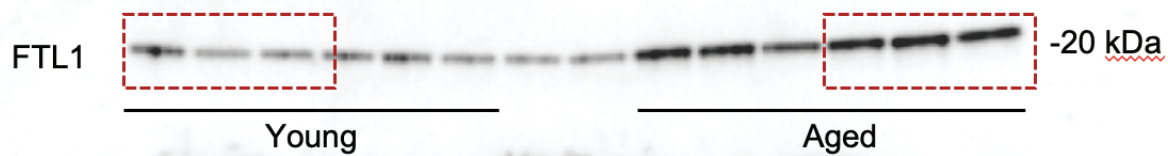

Figure 1g

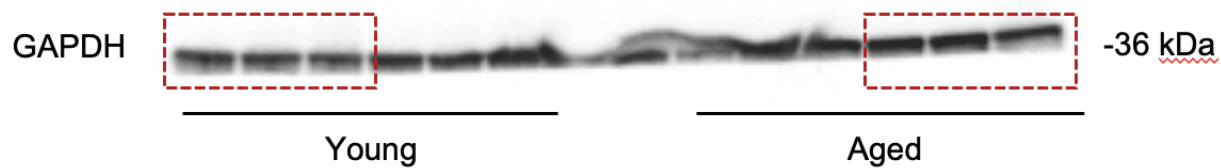

Figure 2b

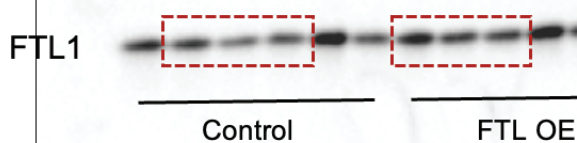

Figure 3b

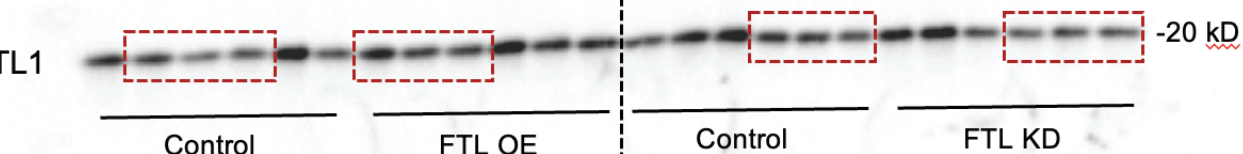

Figure 3h

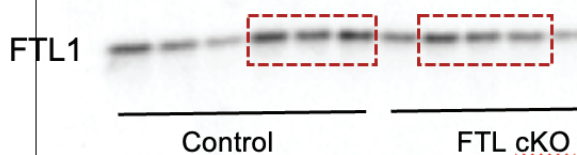

Figure 2b

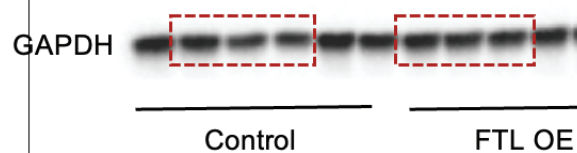

Figure 3b

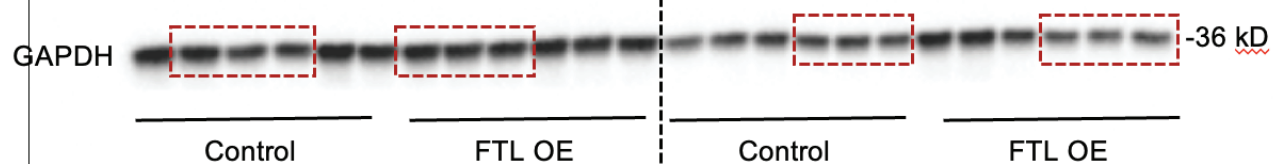

Figure 3h

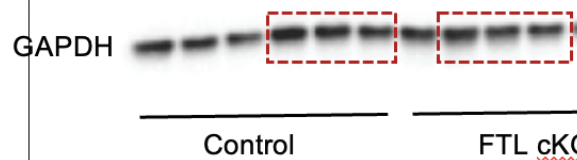

Supplement: Supplementary file 3 — Unprocessed western blots for Figs. 1–3. [file 43587_2025_940_MOESM3_ESM.pdf]
