# Supplementary material for: Targeting iron-associated protein Ftl1 in the brain of old mice improves age-related cognitive impairment
Source: Nat Aging. 2025 Aug 19;5(10):1957–69. doi: 10.1038/s43587-025-00940-z (PMC12532579; doi:10.1038/s43587-025-00940-z)

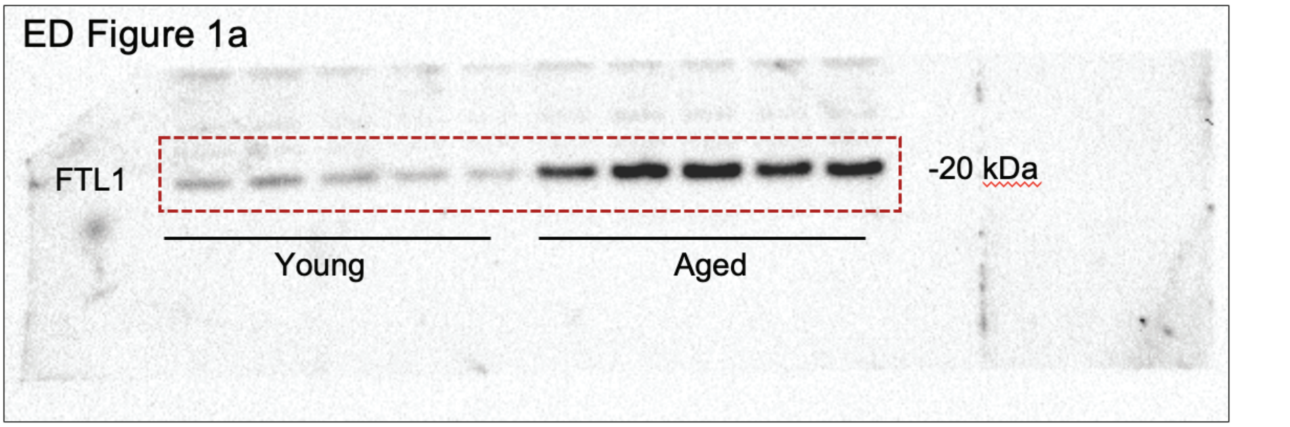
ED Figure 1a
FTL1
-20 kDa
Young
Aged

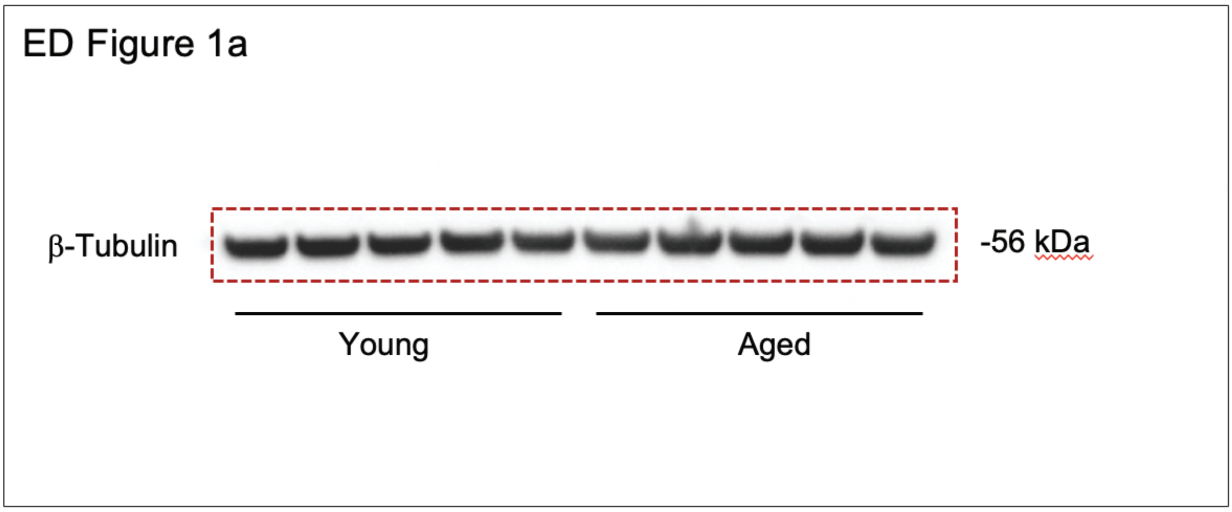
ED Figure 1a
β-Tubulin
-56 kDa
Young
Aged

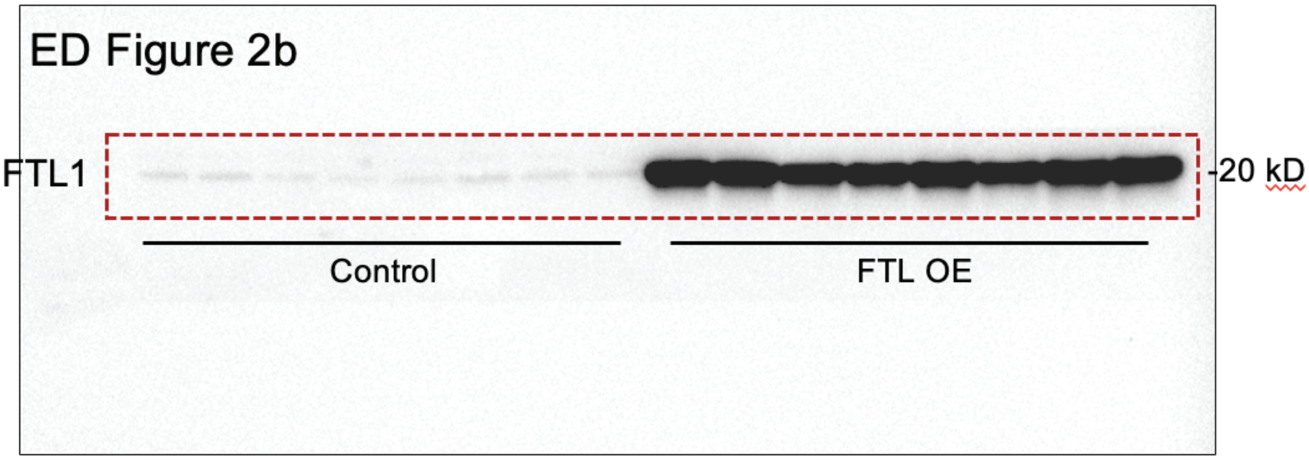
ED Figure 2b
FTL1
-20 kD
Control
FTL OE

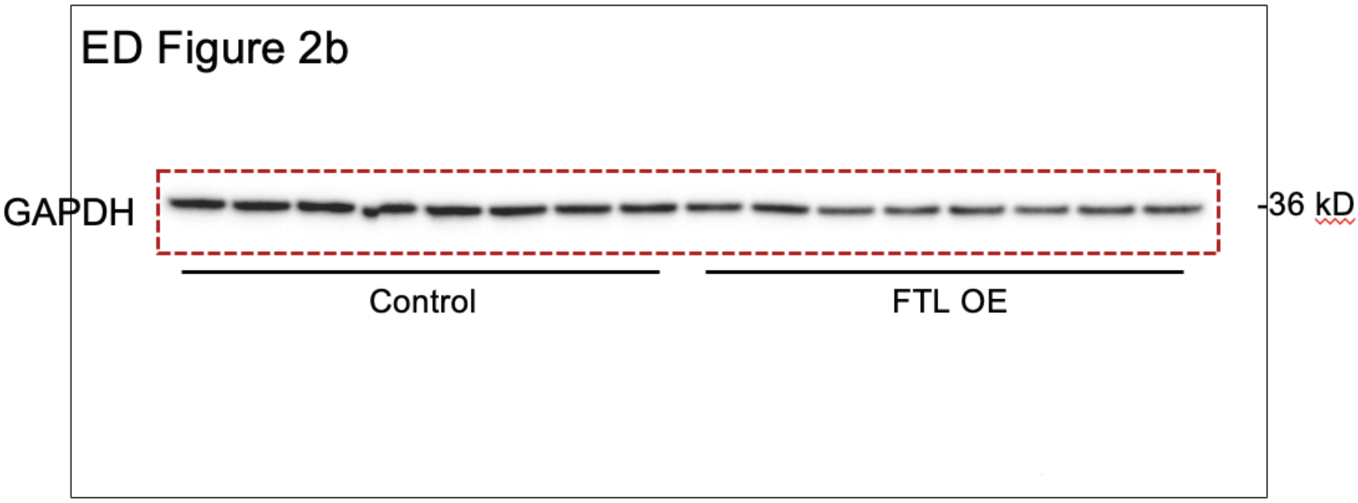
ED Figure 2b
GAPDH
-36 kD
Control
FTL OE

ED Figure 3c

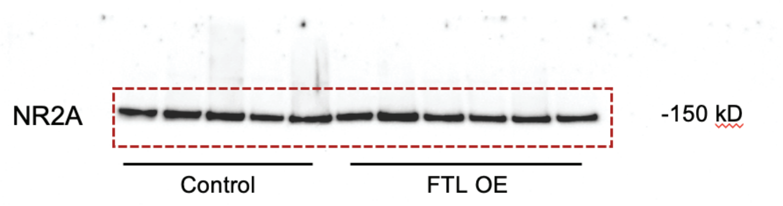

ED Figure 3c

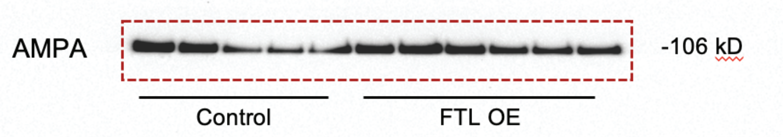

ED Figure 3c

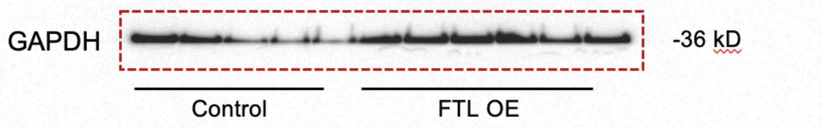

ED Figure 3c

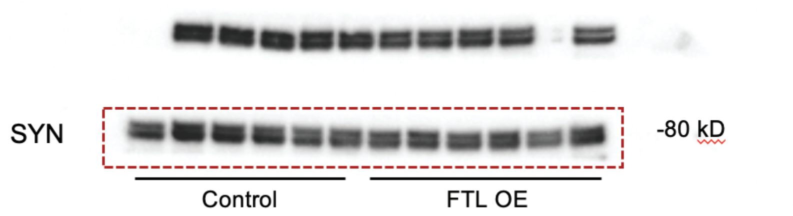

ED Figure 3c

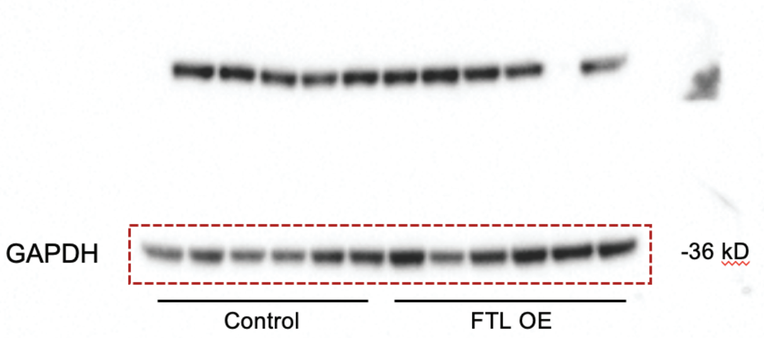

ED Figure 4c

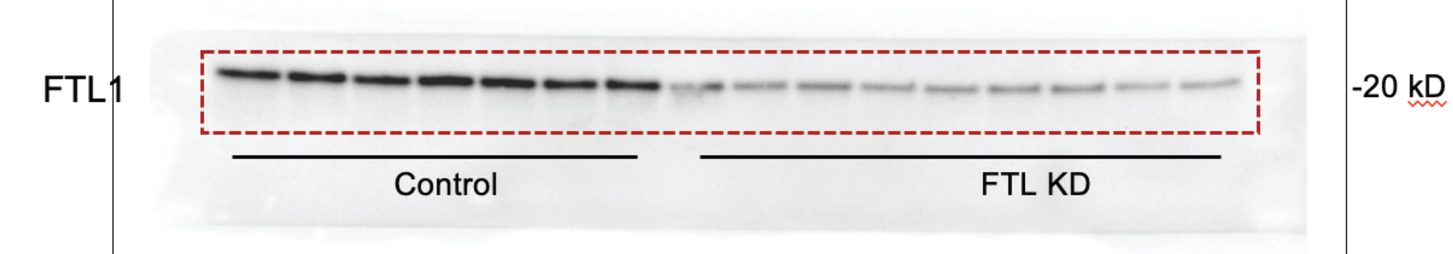

ED Figure 4c

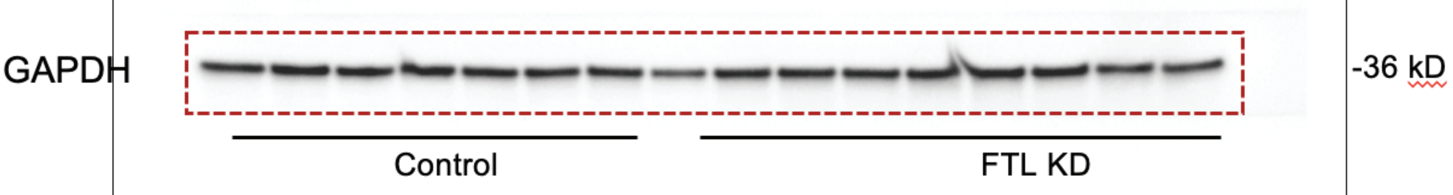

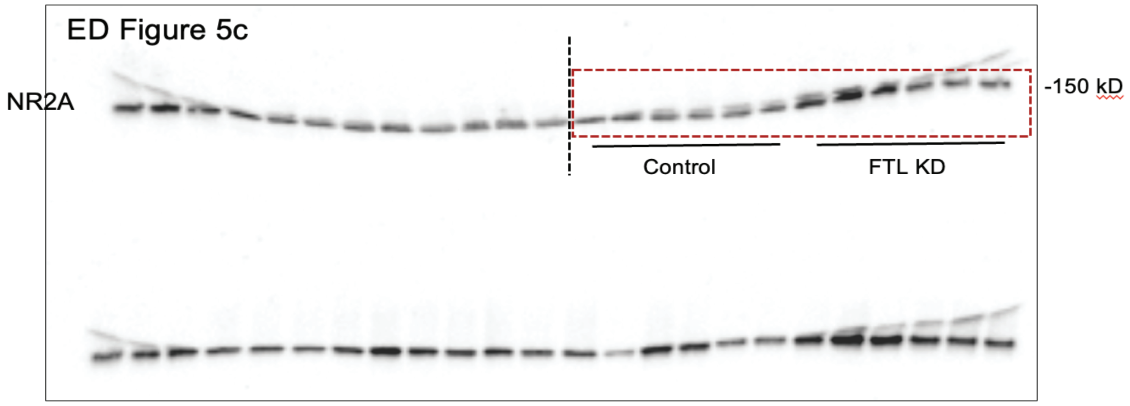
ED Figure 5c
NR2A
-150 kD
Control
FTL KD

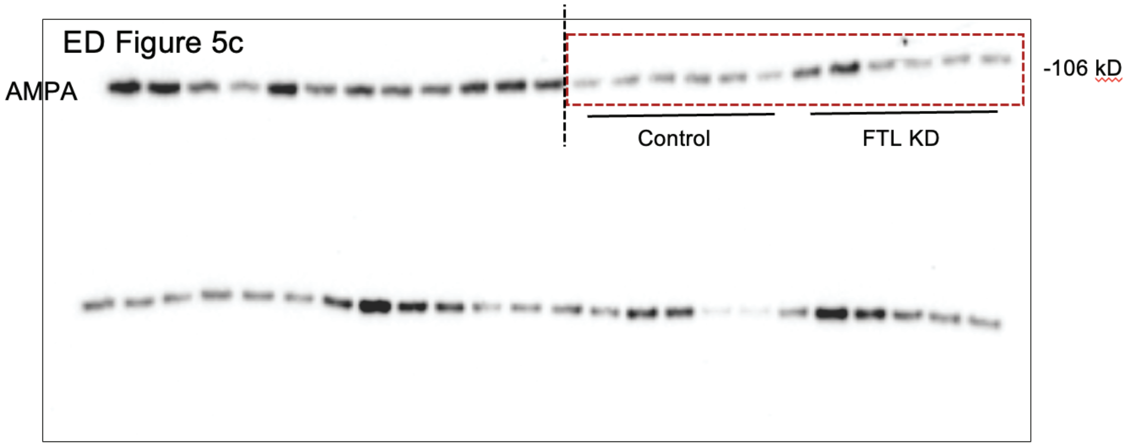
ED Figure 5c
AMPA
-106 kD
Control
FTL KD

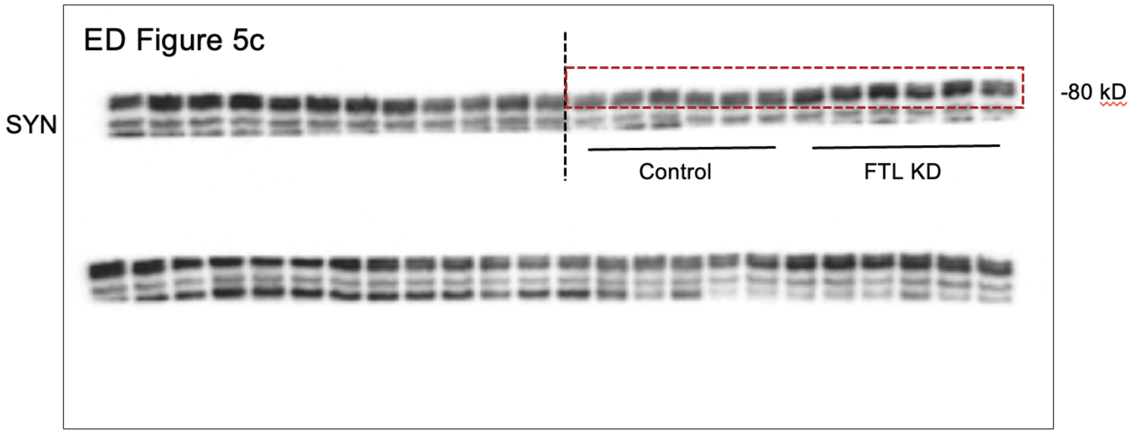
ED Figure 5c
SYN
-80 kD
Control
FTL KD

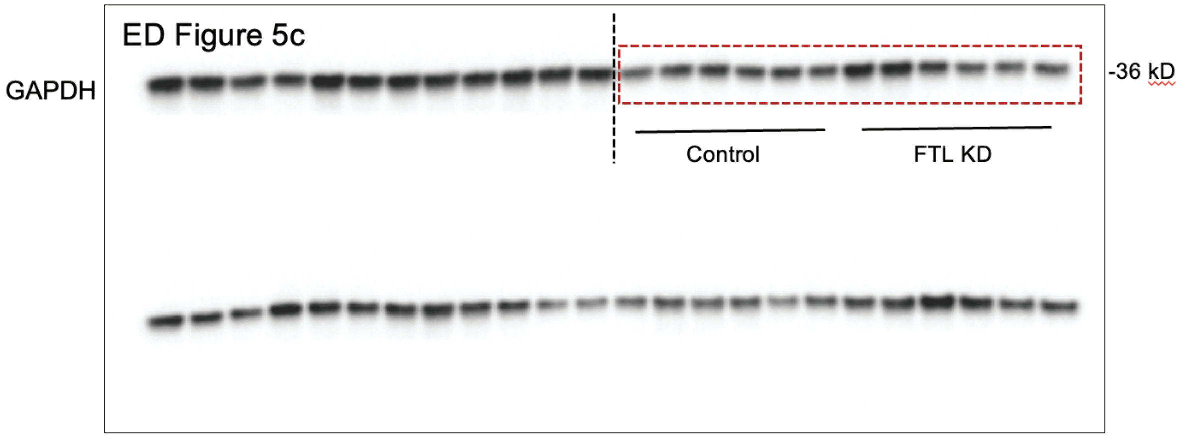
ED Figure 5c
GAPDH
-36 kD
Control
FTL KD

ED Figure 6c

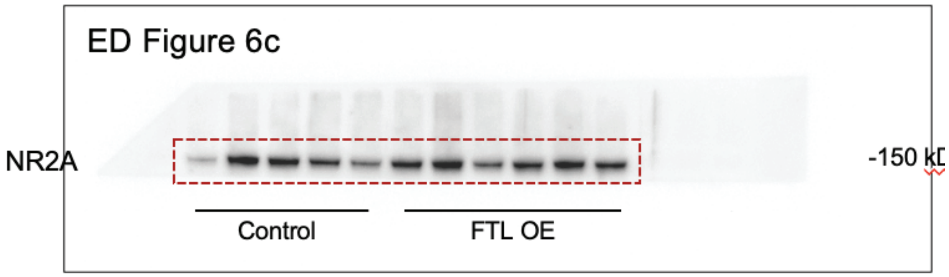

ED Figure 6c

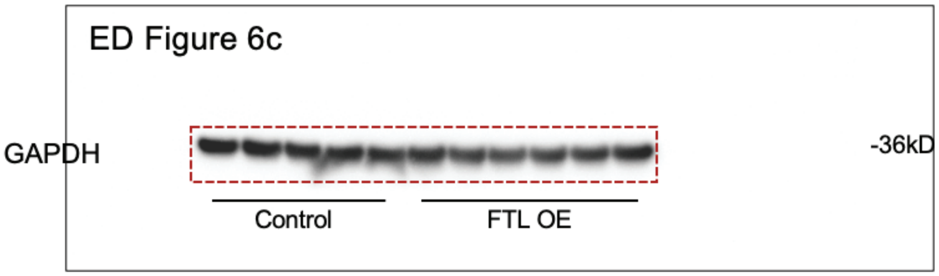

ED Figure 6c

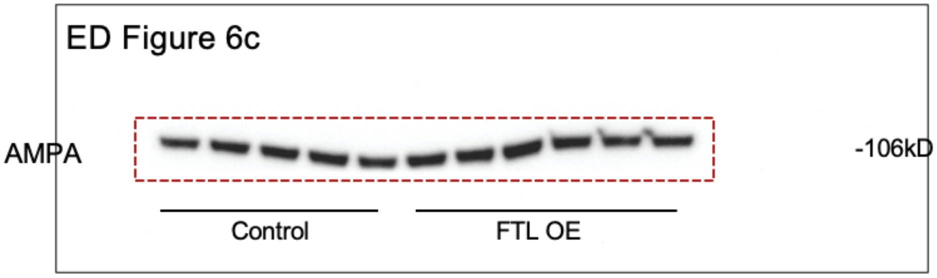

ED Figure 6c

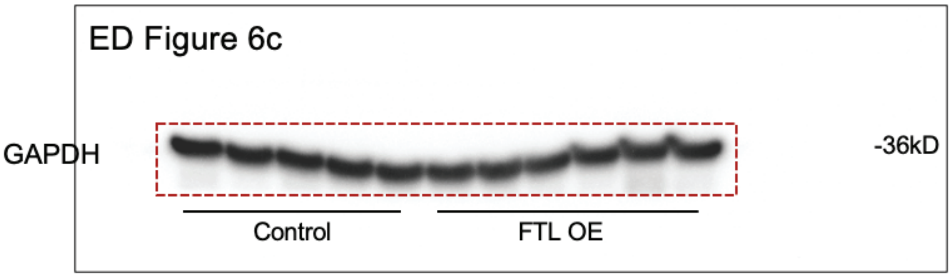

ED Figure 6c

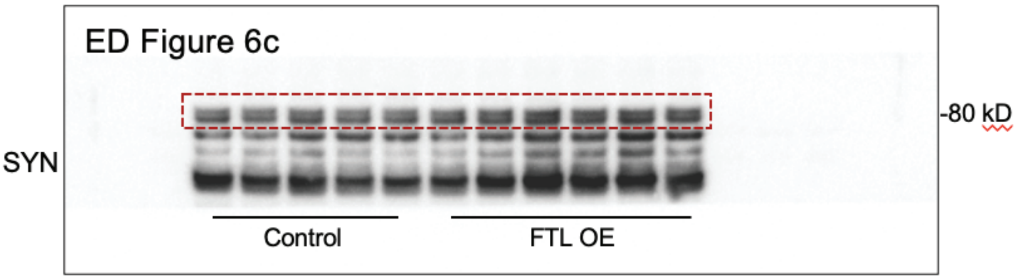

ED Figure 6c

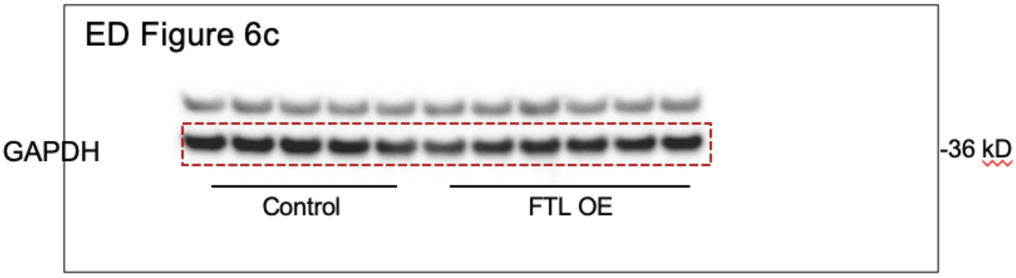

Supplement: Supplementary file 4 — Unprocessed western blots for Extended Data Figs. 1–6. [file 43587_2025_940_MOESM4_ESM.pdf]
